# A Multicentre Randomized Controlled Trial of the Efficacy and Safety of Single-Dose Praziquantel at 40 mg/kg vs. 60 mg/kg for Treating Intestinal Schistosomiasis in the Philippines, Mauritania, Tanzania and Brazil

**DOI:** 10.1371/journal.pntd.0001165

**Published:** 2011-06-14

**Authors:** Piero L. Olliaro, Michel T. Vaillant, Vincente J. Belizario, Nicholas J. S. Lwambo, Mohamed Ouldabdallahi, Otavio S. Pieri, Maria L. Amarillo, Godfrey M. Kaatano, Mamadou Diaw, AnaLucia C. Domingues, Tereza C. Favre, Olivier Lapujade, Fabiana Alves, Lester Chitsulo

**Affiliations:** 1 UNICEF/UNDP/World Bank/WHO Special Programme on Research and Training in Tropical Diseases (TDR), World Health Organization, Geneva, Switzerland; 2 Methodology and Statistical Unit, Center for Health Studies, Centre de Recherche – Santé, Strassen, Luxembourg; 3 National Institutes of Health, University of the Philippines Manila, Manila, Philippines; 4 National Institute for Medical Research, Mwanza Medical Research Centre, Mwanza, Tanzania; 5 Nutrition et Actes Médicaux, Institut National de Recherches en Santé Publique (INRSP), Nouakchott, Mauritania; 6 Laboratorio de Eco-Epidemiologia e Controle da Esquistossomose e Geohelmintoses, Instituto Oswaldo Cruz, FIOCRUZ, Rio de Janeiro, Brazil; 7 Department of Clinical Epidemiology, College of Medicine, University of the Philippines, Manila, Philippines; 8 Ministère de la Santé/P.E.V., Nouakchott, Mauritania; 9 Departamento de Medicina Clinica, Centro de Ciencias da Saude, UFPE, Hospital das Clinicas, Recife, Brazil; 10 Latin America Regional Office, Drugs for Neglected Diseases Initiative, Rio de Janeiro, Brazil; 11 Preventive Chemotherapy and Transmission Control Unit, Control of Neglected Tropical Diseases, World Health Organization, Geneva, Switzerland; Queensland Institute for Medical Research, Australia

## Abstract

**Background:**

Praziquantel at 40 mg/kg in a single dose is the WHO recommended treatment for all forms of schistosomiasis, but 60 mg/kg is also deployed nationally.

**Methodology/Principal Findings:**

Four trial sites in the Philippines, Mauritania, Tanzania and Brazil enrolled 856 patients using a common protocol, who were randomised to receive praziquantel 40 mg/kg (n = 428) or 60 mg/kg (n = 428). While the sites differed for transmission and infection intensities (highest in Tanzania and lowest in Mauritania), no bias or heterogeneity across sites was detected for the main efficacy outcomes. The primary efficacy analysis was the comparison of cure rates on Day 21 in the intent-to-treat population for the pooled data using a logistic model to calculate Odd Ratios allowing for baseline characteristics and study site. Both doses were highly effective: the Day 21 cure rates were 91.7% (86.6%–98% at individual sites) with 40 mg/kg and 92.8% (88%–97%) with 60 mg/kg. Secondary parameters were eggs reduction rates (ERR), change in intensity of infection and reinfection rates at 6 and 12 months. On Day 21 the pooled estimate of the ERR was 91% in both arms. The Hazard Ratio for reinfections was only significant in Brazil, and in favour of 60 mg/kg on the pooled estimate (40 mg/kg: 34.3%, 60 mg/kg: 23.9%, HR = 0.78, 95%CI = [0.63;0.96]). Analysis of safety could not distinguish between disease- and drug-related events. 666 patients (78%) reported 1327 adverse events (AE) 4 h post-dosing. The risk of having at least one AE was higher in the 60 than in the 40 mg/kg group (83% vs. 73%, p<0.001). At 24 h post-dosing, 456 patients (54%) had 918 AEs with no difference between arms. The most frequent AE was abdominal pain at both 4 h and 24 h (40% and 24%).

**Conclusion:**

A higher dose of 60 mg/kg of praziquantel offers no significant efficacy advantage over standard 40 mg/kg for treating intestinal schistosomiasis caused by either *S. mansoni* or *S. japonicum*. The results of this study support WHO recommendation and should be used to inform policy decisions in the countries.

**Trial Registration:**

Controlled-Trials.com ISRCTN29273316 ClinicalTrials.gov NCT00403611

## Author Summary

Control of urinary and intestinal schistosomiasis is based on mass administration of praziquantel at the World Health Organization (WHO) recommended dose of 40 mg/kg, though some countries use 60 mg/kg. This multi-country randomized clinical trial compared the efficacy (cure and egg reduction rates three weeks post-treatment) and safety of these two doses for treating intestinal schistosomiasis in 856 patients in Brazil, Mauritania and Tanzania (*Schistosoma mansoni*), and The Philippines (*S. japonicum*). Transmission and infection intensities varied across the sites, but there was no bias or heterogeneity in efficacy outcomes. The two doses are equally effective in curing intestinal schistosomiasis; the higher dose may be less well tolerated, though effects are generally mild and transient. In endemic areas people can be re-infected; one year post-treatment patients on 60 mg/kg had fewer re-infections but this finding is difficult to explain. This study was conducted to respond to the demand for evidence about the dose of praziquantel when deployed in endemic countries. The results, along with those of systematic reviews, support the current WHO recommendation for using praziquantel at 40 mg/kg and should inform policy decisions in countries. The Philippines has already changed from 60 to 40 mg/kg after this study.

## Introduction

Schistosomiasis is a parasitic infection caused by blood flukes (flatworms) of the class Trematoda: *Schistosoma haematobium* (causing urinary schistosomiasis), *S. mansoni*, *S. japonicum*, *S. intercalatum* and *S. mekongi* (causing intestinal schistosomiasis). A recent systematic review of evidence estimates that ∼207 million people (97% in Africa) are affected and ∼779 million people are at risk (85% in Africa) in 76 countries (46 in Africa). With respect to the mid 1990s, there has been an increase of ∼7.3% and 10.9% in infections and population at risk (mostly accounted for by Africa) but a concomitant (slight) decrease from 29.6% to 26.6% of the ratio between people infected and people at risk, primarily as the combined result of socio-economic development and interventions such as sanitation and broader distribution of praziquantel [1].

Traditionally, schistosomiasis has been attributed a low burden of disease as quantified by DALY (disability adjusted life years) lost (1,760,000 estimated in 2002 [2]). However, the real impact of schistosomiasis infection on people's health and performance is more difficult to quantitate. A systematic review and meta-analysis of disability-related effects of schistosomiasis indicates that the disability weight of schistosomiasis (2–15%) is much greater than the previous estimation of 0.5% [3]. Specifically for *S. japonicum*, a disability rate of 13% has been calculated (7–46 times greater than current estimates) [4].

Today praziquantel is the mainstay of schistosomiasis control. However, while the World Health Organization (WHO) currently recommends that it should be used in a single dose of 40 mg/kg for the treatment of both urinary and intestinal schistosomiasis [5], different doses of praziquantel are being deployed by national control programmes.

Studies of the dose-response of praziquantel in urinary and intestinal schistosomiasis are few and incomplete. In particular, no obvious dose-effect was apparent in a Cochrane systematic review and meta-analysis of randomised controlled trials of praziquantel between the doses of 20 to 40 mg/kg for urinary schistosomiasis [6]. A Cochrane systematic review and meta-analysis of treatments for intestinal schistosomiasis is under way. Here, contrary to urinary schistosomiasis, preliminary results (which were not available when this study was being planned and conducted) show a dose-response effect between the doses of 20, 30 and 40 mg/kg with no further gain with doses >40 mg/kg.

This study was set up to assess whether using praziquantel at 60 mg/kg for intestinal schistosomiasis (caused by either *S. japonicum* or *S. mansoni*) offers advantages over 40 mg/kg. Four trial sites in the Philippines, Mauritania, Tanzania and Brazil enrolled patients using a common protocol. The study at each site was powered to show a difference in cure rates between dosage groups. The analytical plan was prospectively designed to allow for reporting the results both for each study site and combined by individual patients' data meta-analysis (IPD).

## Materials and Methods

### Study sites

The studies were conducted in:

- the province of Agusan del Sur in Mindanao Island, Philippines (rural, *S. japonicum* with year round transmission during June to September, prevalence 7–11%)
- the Mwanza region, Tanzania (rural, *S. mansoni* (year-round) and *S. haematobium* (February to March), prevalence 34 to 65%)
- the Trarza region, Mauritania (rural, *S. mansoni*, prevalence 18.7%, *S.haematobium* prevalence 30.9%, Coinfection 7.3% and total prevalence 57%)
- Municipality of São Lourenço da Mata, Pernambuco state, North-eastern Brazil (urban, *S. mansoni* (year round), prevalence 25%).

The prevalences above are from historical data. The actual point prevalence found on screening for this study for each site is reported in the Results section.

### Patients

#### Inclusion criteria

Aged 10–19 years; confirmed *Schistosoma* infection with ≥100 eggs per gram of faeces (epg) using Kato Katz technique (*S. japonicum* in the Philippines; *S. mansoni* in the others, with mixed infections with *S. haematobium* allowed in Mauritania); written informed consent; able and willing to be examined on follow-up visits and to provide stool samples.

#### Exclusion criteria

Pregnant or lactating; previous history of adverse reaction associated with praziquantel; history of acute or chronic severe disease including hepato-splenic schistosomiasis; use of praziquantel within the last 30 days or any other medication that may affect the results of trial (e.g. antibiotics) within the past week; symptomatic malaria; mixed *S. mansoni* and *S. haematobium* infections in Tanzania.

### Parasitological diagnosis

At baseline, Day 21, 6 months and 12 months, stool samples were taken on two consecutive days and each tested twice. The mean egg counts are reported.

### Study drug

Praziquantel (Distocide® by Shin-Poong, Korea) was procured by WHO and administered with food after weighing the participants on a scale by the nearest half tablet according to the randomization schedule.

### Study outcomes

Efficacy. (i) Primary: cure rates (“complete cure” defined as negative stools for *Schistosoma* eggs) and egg reduction rates (ERR, “partial cure”) with the two regimens on Day 21; (ii) Secondary: reinfection rates at 6 months and 12 months after treatment. Safety and tolerability: prevalence and intensity of adverse events (AEs).

The choice of Day 21 as the main efficacy outcome is justified by the fact that, as praziquantel is not active on immature schistosomes of less than 24 days, by Day 21 immature *S. mansoni* worms will not have yet matured into patent egg-producing infections. The evaluation of praziquantel efficacy, using duplicate Kato-Katz slides from two different stool samples, at 3 weeks showed the highest cure and egg-reduction rates [7].

### Recruitment procedures

Screening occurred in the village in Brazil and Mauritania and at school in the Philippines and Tanzania.

### Randomization

Computer generated randomization list with blocks of 4 in a ratio 1∶1 for each regimen.

### Allocation concealment

Sealed and numbered envelopes were kept in a locked cabinet by one responsible person; two different people preparing treatment and evaluating patients; stool specimens read by a technician blinded as to the treatment.

### Sample size calculation

The sample size required at each site was computed at 91 patients per treatment group using 60% and 80% as the cure rates of praziquantel 40 mg/kg and 60 mg/kg respectively, with 80% power and 95% confidence. The sample size was adjusted to 109 individuals per treatment group for a total of 218 participants in consideration of the anticipated 20% of participants who might be lost to follow-up.

### Assessment of risk of bias

The results were expressed as Odds Ratios (OR and 95% confidence intervals (CI)) for dichotomous outcomes. Adjusted outcomes (such as cure rates and hazard ratios [HR]) were analysed by using an inverse variance methodology. Continuous outcomes were expressed as means and standard deviations.

Study bias was examined through the use of a funnel plot of the log-transformed OR/HR [log(OR)/Log(HR)] of individual studies against the precision (1/SE, standard error). Funnel plot asymmetry was further tested by using the Egger's method.

For the assessment of heterogeneity, the Cochran's Q and I^2^ test of heterogeneity were performed to detect non-homogeneity between the estimates of individual studies and by using fixed effects models. The Mantel-Haenszel method was used to analyse cure rates. ORs of adjusted cure rates allowing for age, gender, baseline diarrhoea and nausea were obtained by the generic inverse variance method. An inverse variance method was also used to analyse the difference between groups in the mean of the difference between eggs-per-gram (epg) at Day 21 and Day 0.

The Mantel-Haenszel method was used to analyse the reinfection rates at day 180 and day 360. The HRs of reinfection provided by a Cox proportional hazard model were aggregated by using the generic inverse variance method.

### Analyses

While the data were also analysed for each individual study, in this paper we present specifically the results of the prospectively defined individual patient data meta-analysis whereby the analysis was performed by using the same methods as for each study site (see below) adjusted for the site.

#### Population

Efficacy and safety analyses were conducted on the Intent-to-Treat (ITT) population. Missing or incomplete data were considered as missing.

The same analyses were also conducted on per-protocol populations (PP1 = All patients randomized and evaluable at the Day 21 visit with no major protocol violations; PP2 = All patients randomized and evaluable at the 12 months visit with no major protocol deviations) - not reported in this paper as results are comparable with the ITT analysis.

#### Study profile and baseline characteristics

Patient attrition throughout the study was reported according to the Consort guidelines, accounting for drop-outs and exclusions (in diagrammatic form).

The baseline characteristics of the patients enrolled are reported, including basic demography (sex and age) and pre-treatment values for egg counts and intensities of infection by treatment group. Continuous variables are presented as mean +/− standard deviation.

While allocation to treatment was by computer-generated random assignment, we checked for possible imbalance between the groups in terms of baseline conditions and drop-out rates. Group comparison was carried out using the Student t-test. In the case of a non-significant Kolmogorov-Smirnov test for normal data or non-homogeneity of the variances (folded F-test) between groups, a logarithmic transformation was used. A non-parametric Wilcoxon sign rank test for matched pairs was performed if the student t-test applying conditions were still not respected. The Chi-square test was used to compare frequencies.

### Analyses of efficacy

#### Data presentation

Continuous data are presented as mean and standard deviation. Dichotomous or multiple categories data are presented as frequencies and %.

Cure rates are presented as % cured with 95% confidence intervals (CIs).

Egg count reduction was estimated as: [1−(epg2/epg1)×100] where epg1 and epg2 are the geometric mean of log10 transformed (x+1) numbers of eggs per gram of faeces at pre-treatment (D0) and Day 21 post-treatment, respectively.

A logarithmic transformation of the egg count values at Day 0 and Day 21 was done. A value of 1 was added to each egg count value so that the logarithm of zero egg count (for negative result) could be computed.

#### Cure rates

A Chi-square test (with correction for continuity) was used to compare the cure rates at day 21 with 60 mg/kg vs. 40 mg/kg of praziquantel. The relative rates on Day 21 between the two groups are presented as OR with 95% CIs. A logistic model was used to evaluate the adjusted OR and 95%CIs on baseline characteristics such as sex, age, egg counts, intensity of infection and highly frequent signs, symptoms or positive physical examination.

For the IPD meta-analysis, the study site was included in the model. A generalized linear model using a logit link function was used if heterogeneity was not found; otherwise, a generalized linear mixed model including random effects was used. In the latter, treatment effect and the interaction of treatment and study site were tested as random effects in the model.

#### Egg counts

The mean log egg counts at Day 0 and Day 21 were compared using the analysis of variance for repeat measures to determine if there was a difference between the two treatment regimens at baseline (D0) and Day 21. An ANCOVA model of the D21 and D0 difference with the D0 value as covariate and treatment group as factor was used as a sensitivity analysis of the repeated measures ANOVA. The egg reduction rates (ERR) at Day 21 were calculated and presented descriptively for the two arms.

For the IPD meta-analysis a general linear model including study site effect was used. In case of heterogeneity, a model with random effects was used.

#### Intensity of infection

The intensity of infection at Day 0 and Day 21 of patients given praziquantel 60 mg/kg or 40 mg/kg was compared using the ordered logistic regression analysis. The categories used were: light (1–100 eggs per gram of faeces); moderate (101–400 epg); heavy (>400 epg).

For the IPD meta-analysis the intensity of infection at Day 0 and Day 21 of patients given praziquantel 60 mg/kg single dose regimen and praziquantel 40 mg/kg single dose regimen was compared using a linear model for polytomous dependent variable using a logit link function and mixed effects if heterogeneity was found between study sites and adjusted for the study site.

#### Reinfections

Two approaches were used.

The time to reinfection post-Day 21 was investigated by using Kaplan-Meier product limit estimates. The median infection-free survival time was calculated with 95%CIs. Hazard ratios (HR) of reinfection adjusted by baseline characteristics such as sex, age, egg counts, intensity of infection and frequently reported signs, symptoms or positive physical examination was examined with a Cox proportional hazard model (adjusted for the study site for the pooled estimate).

We also calculated the rate of infections on Day 180 and on Day 360 at each site and on the pooled data and compared treatment arms using the Mantel-Haenszel fixed effect OR with 95% CIs.

In addition we calculated the intensity of infection occurring during follow-up as described above.

### Analysis of safety

Safety data were gathered through a questionnaire enquiring on the occurrence of adverse events (AEs) on Day 0 at 4 hours, Day 1 and Day 21 post-dosing. An AE was defined as any unfavourable and unintended sign (including an abnormal laboratory finding), symptom, or disease temporally associated with the use of a medicinal or investigational product, whether or not related to that product. It is clear that this approach does not allow to distinguish between disease-related and drug-related events, but only allows comparing arms for frequency of events, independent of causality.

Recording of the AEs included: (1) date and time of onset, (2) duration, (3) severity, (4) severity and (5) relationship to treatment. The report also includes a probable explanation from the investigator as to the cause of the AE.

The prevalence and intensity of the following signs and symptoms are assessed: abdominal discomfort, nausea, vomiting, diarrhoea, anorexia, fever (using an oral thermometer), headache, dizziness and allergic reaction. The severity of the signs and symptoms was categorized as “mild”, “moderate”, “severe” and “life-threatening”. The relationship of the signs and symptoms to treatment was categorized as “not related”, “unlikely”, “possible”, “probable” and “most probable”. For the purpose of this study, the cumulative prevalence of AEs is defined as the proportion of those followed-up reporting one or more AEs.

The cumulative prevalence of AEs, defined as the presence of at least one AE in a patient, was determined at 4 hours on Day 0, Day 1 and Day 21 post-treatment.

AEs, signs and symptoms were classified according to the WHO Adverse Reaction Terminology dictionary.

### Ethics

Patients were explained the scope of the study and signed a written informed consent (if under 18 years of age, written informed consent from parents/guardians and individual verbal assent) before inclusion in the study. The studies were conducted according to the Helsinki declaration and were approved by the local (University of the Philippines Manila - College of Medicine Institutional Review Board, ethics committee of the Institut National de Recherches en Santé Publique in Mauritania, National Medical Research Coordinating Committee in Tanzania, Research Ethics Committee of the Aggeu Magalhaes Research Centre, Oswaldo Cruz Foundation (Fiocruz) in Brazil) as well as the WHO ethics committees. All sites except Tanzania were independently monitored.

## Results

**Figure 1:**
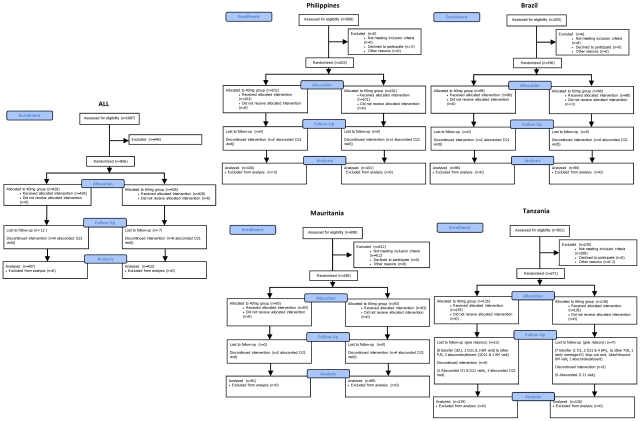
Patient attrition. Consort flow diagram of patient attrition * One protocol violation at Day 21 had no data.

First Patient First Visit and Last Patient Last Visit were August 2005–August 2006 in the Philippines, August 2005–December 2006 in Mauritania, August 2005–September 2006 in Tanzania and March 2006–December 2007 in Brazil. A total of 856 patients were randomised at the four sites to receive praziquantel 40 mg/kg (n = 428) or 60 mg/kg (n = 428). The details by site along with patients' attrition and baseline characteristics are presented in Figure 1 and Table 1 respectively.

**Table 1:** Baseline characteristics.

		40 mg/kg		60 mg/kg		p-value
Age (mean ± SD)	Philippines	12.5	±2.0	12.4	±2.0	0.64
	Brazil	15.2	±2.8	15.0	±2.5	0.57
	Mauritania	12.6	±2.0	12.6	±2.1	1.00
	Tanzania	12.3	±1.8	12.7	±2.1	0.07
	ALL	13.1	±2.4	13.1	±2.4	0.71*
Gender N (%)	Philippines	52.0	(51.0%)	53.0	(52.5%)	0.83
(Males)	Brazil	55.0	(56.1%)	53.0	(54.1%)	0.96
	Mauritania	40.0	(43.0%)	45.0	(48.4%)	0.46
	Tanzania	50.0	(37.0%)	67.0	(49.3%)	0.04
	ALL	197.0	(46.0%)	218.0	(50.9%)	0.15*
Weight (mean ± SD)	Philippines	32.2	±8.8	31.4	±9.6	0.53
	Brazil	49.0	±12.8	48.2	±12.1	0.67
	Mauritania	32.7	±9.4	32.6	±9.0	0.89
	Tanzania	33.3	±6.9	33.3	±7.7	1.00
	ALL	36.5	±11.7	36.1	±11.6	0.53*
Epg (mean ± SD)	Philippines	339.6	±381.0	337.2	±345.6	0.91
	Brazil	441.6	±455.8	476.6	±475.1	0.72
	Mauritania	278.3	±273.0	378.4	±510.2	0.08
	Tanzania	813.5	±713.0	838.8	±789.1	0.83
	ALL	488.4	±542.7	531.3	±610.1	0.59*
Intensity of infection						
Philippines	moderate	77	(76.2%)	78	(76.5%)	0.97
	heavy	24	(23.8%)	24	(23.5%)	
Brazil	light	0.0	(0.0%)	1.0	(1.0%)	0.83
	moderate	65.0	(66.3%)	63.0	(64.3%)	
	heavy	33.0	(33.7%)	34.0	(34.7%)	
Mauritania	light	0.0	(0.0%)	0.0	(0.0%)	0.38
	moderate	75.0	(80.6%)	70.0	(75.3%)	
	heavy	18.0	(19.4%)	23.0	(24.7%)	
Tanzania	light	0.0	(0.0%)	0.0	(0.0%)	0.48
	moderate	48.0	(35.6%)	54.0	(39.7%)	
	heavy	87.0	(64.4%)	82.0	(60.3%)	
ALL	light	0.0	(0.0%)	1.0	(0.2%)	0.94*
	moderate	266.0	(62.1%)	264.0	(61.7%)	
	heavy	162.0	(37.9%)	163.0	(38.1%)	

*General linear mixed models were used to account for the country.

The point prevalence of intestinal schistosomiasis as derived from the screening logs was: in Brazil 309/636 (48%); in Tanzania 466/501 (93%); in Mauritania 112/598 (18.7%) for *S. mansoni* pure infection (*S. haematobium* was 185/598 (30.9%) and co-infections were 44/598 (7.3%) for an overall prevalence of schistosomiasis of 341/598 (57%); in the Philippines 588/1848 (32%).

The age distribution in each treatment group and gender category showed that patients aged 10–15 years were over-represented with respect to a normal distribution in the Philippines, Mauritania and Tanzania whereas in Brazil the age distribution was more homogeneous across all ages (10–19 years). There was no significant difference in age distribution between treatment groups.

No gender imbalance was apparent except in Tanzania where males were more prevalent in the 60 mg/kg group (borderline significance). Fewer males were recruited in Mauritania than in the other countries (40–45% vs. 50–55%).

Weight was not significantly different between treatment groups. Patients in Brazil were 3 years older and 15 kg heavier than in the other countries.

Pre-treatment eggs of schistosoma per gram (epg) of faeces and intensity of infection were similar between the treatment groups in the four countries and between the pooled estimates (see below).

### Analysis of bias and heterogeneity

While there are only four points to draw the funnel plot, the cure rates are symmetrical and their distribution is narrow - that is, a low risk of bias. The funnel plot of the adjusted Day 21 cure rates is presented as an annex (Figure S1). Similar results were obtained with the crude rates, the epg, and the hazard ratios of reinfection (not shown). As for the reinfection rates on follow-up, there was no bias on Day 180 but bias was found on Day 360.

No substantial heterogeneity (defined as I^2^>50%) existed in the pooled analysis of the 4 study sites for the primary outcome: I^2^ for crude cure rates = 8%; adjusted cure rates = 47%; epg differences between Day 21 and Day 0 = 0%, HR of reinfection = 19%, reinfection rates at Day 180 = 23%. Instead, the Higgins I^2^ for reinfection rates at Day 360 was 76%. (see reinfection rates analysis below)

### Efficacy analysis - Day 21

#### Cure rates

The crude (unadjusted) cure rates were 91.7% (from 86.6% in Tanzania to 98% in Brazil) with 40 mg/kg and 92.8% (from 88% in Tanzania to 97% in the Philippines) with 60 mg/kg (p = 0.54 using a general linear mixed model to account for the study site effect) (Table 2).

**Table 2:** Crude (unadjusted) Day 21 cure rates and reduction rates between Day 0 and Day 21.

	40 mg/kg			60 mg/kg			
	%	95%CI		%	95%CI		p-value
Philippines	92.16%	(85.28%;	95.97%)	97.03%	(91.63%;	98.98%)	0.13
Brazil	97.96%	(92.86%;	99.44%)	94.90%	(88.61%;	97.80%)	0.25
Mauritania	91.40%	(83.93%;	95.58%)	92.47%	(85.27%;	96.31%)	0.79
Tanzania	86.55%	(79.27%;	91.55%)	88.00%	(81.14%;	92.59%)	0.73
ALL	91.75%	(88.69%;	94.03%)	92.81%	(89.92%;	94.91%)	0.54*

	Day 21		Egg	Day 21		Egg
	N	GeoMean	Reduction	N	GeoMean	Reduction
Philippines	101	1.09	90.1%	99	1.01	90.8%
Brazil	96	1.00	91.7%	94	1.01	91.8%
Mauritania	92	1.10	89.2%	93	1.13	89.6%
Tanzania	119	1.27	91.9%	125	1.20	92.3%
ALL	408	1.12	90.9%	411	1.09	91.3%

*General linear mixed models were used to account for the country.

ERR: eggs reduction rate.

**Figure 2:**
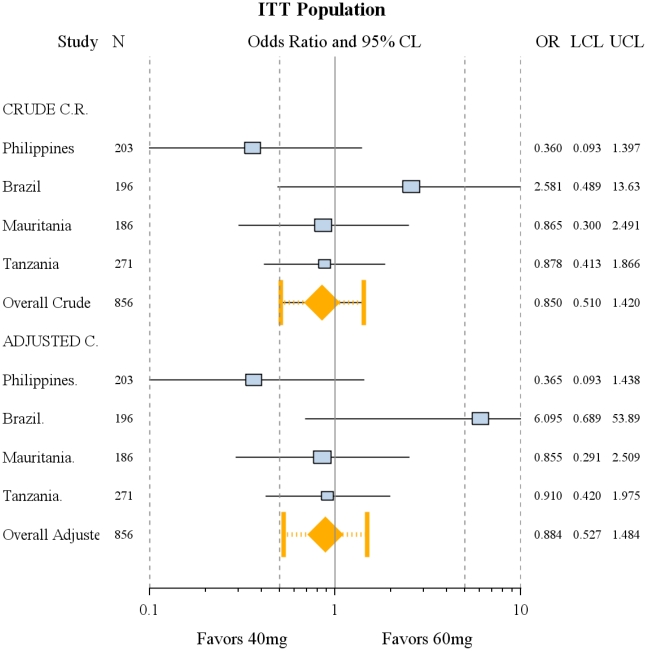
Day 21 crude and adjusted cure rates. Forest plot of the Day 21 crude and adjusted cure rates.

No significant difference was seen in the adjusted (for sex, age, pre-treatment diarrhoea and nausea) ORs of treatment efficacy on Day 21 (complete cure) between 60 mg/kg and 40 mg/kg both for the individual studies and for the pooled estimate. In all cases the 95%CI around each estimate crossed the null boundary (Figure 2). Similarly, no difference was seen with crude (unadjusted) rates in the ITT as well as the per-protocol populations (not shown).

#### Egg counts

**Figure 3:**
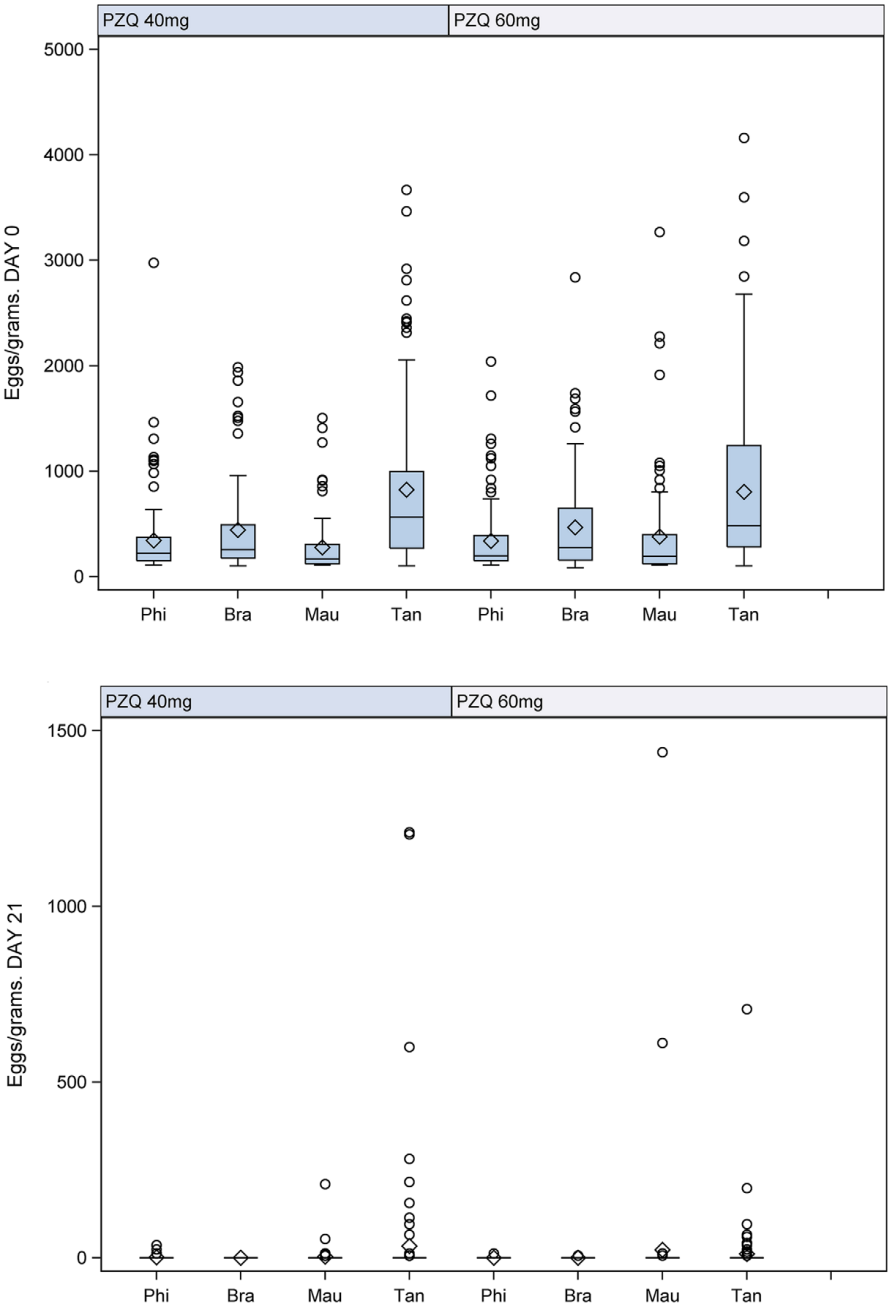
Egg counts between treatment groups at Day 0 and Day 21. Boxplot of egg counts (epg) between treatment groups at Day 0 and Day 21c.

(Figure 3) At Day 0 (pre-treatment) the mean eggs per gram (epg) of faeces ranged between 278±273 and 441±456 in the Philippines, Brazil and Mauritania for the 40 mg/kg arm and between 337±345 and 476±475 for the 60 mg/kg. The epg was highest in Tanzania with 813±713 and 838±789 respectively. Overall, the mean counts were slightly higher in the 60 mg/kg group in Brazil, Mauritania and Tanzania and similar in the Philippines.

At Day 21 post-treatment the counts decreased dramatically. For the 40 mg/kg arm the counts ranged 0 to 3±22 in the Philippines, Brazil and Mauritania but were higher in Tanzania (33±168). In the 60 mg/kg arm, epg in the Philippines and Brazil decreased to almost 0 (0.1±1 and 0.1±0.6, respectively), while in Mauritania and in Tanzania the counts reached 23±161 and 11±66 respectively.

#### Egg reduction rates (ERR)

(Table 2) The highest ERR on Day 21 as compared with Day 0 were 92% for the 40 and the 60 mg/kg treatment group in Brazil and Tanzania. In the other countries they were at least 89%. The pooled estimate was 91%.

#### Intensity of Infection

**Figure 4:**
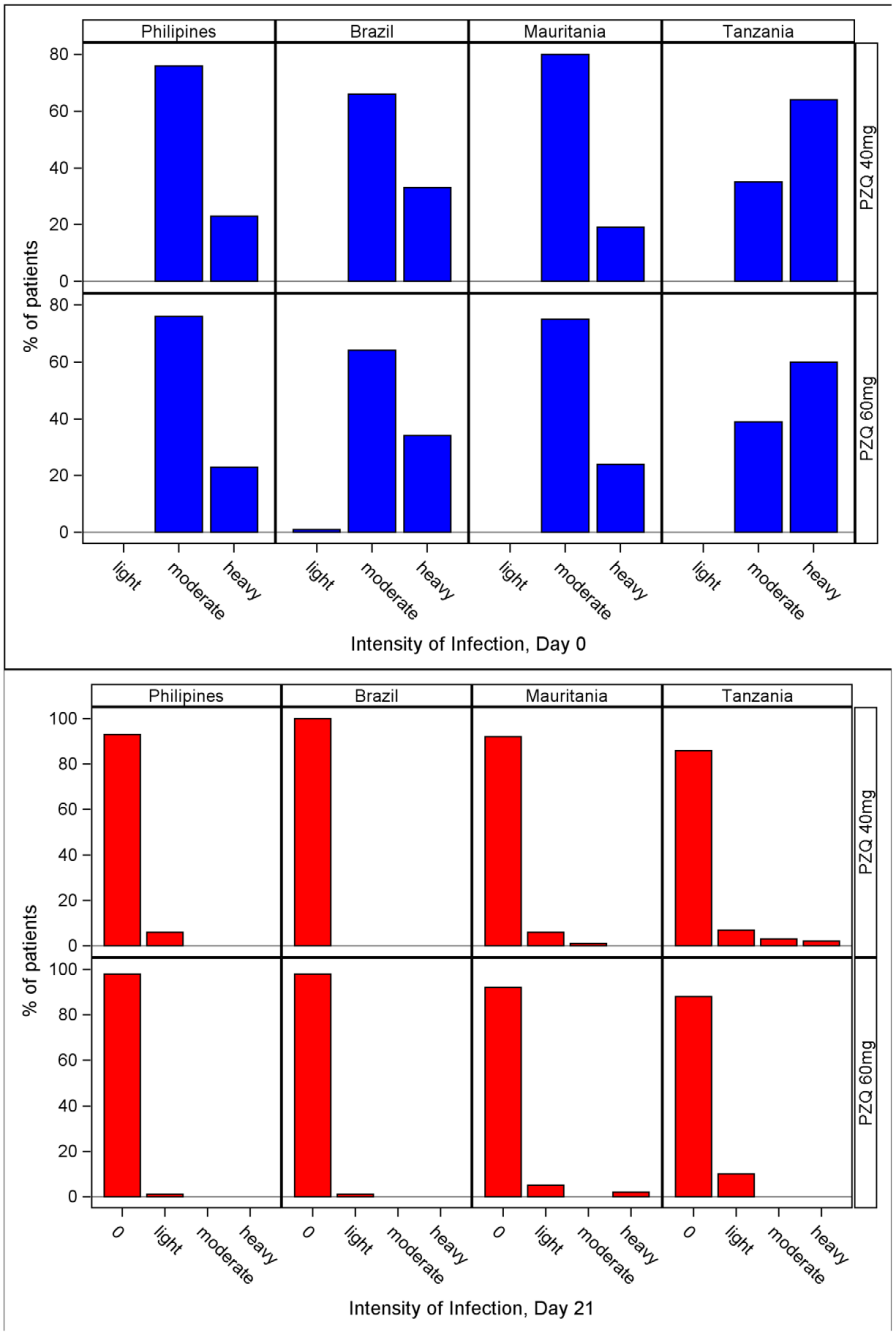
Intensity of infection at Day 0 (pre-treatment) and Day 21. Intensity of infection at Day 0 (pre-treatment) and Day 21 post-treatment by site and treatment group.

(Figure 4) At Day 0, the intensity of infection was mostly moderate in all countries (>60% in the Philippines, Brazil and Mauritania) except Tanzania which had >60% heavy infections. One case of light infection was recorded in Brazil. The pooled estimate has 62% for moderate and 38% for heavy intensity in both the 40 mg/kg and 60 mg/kg groups. There was no significant difference between the treatment groups.

At Day 21 in the Philippines there were 7 light infections in the 40 and 1 in the 60 mg/kg group; in Brazil 1 light infection on 60 mg/kg; in Mauritania the 40 mg/kg group had 6 light and 1 moderate infection, the 60 mg/kg group had 5 light and 2 heavy infections; in Tanzania the 40 mg/kg group had 9 light, 4 moderate and 3 heavy infections and the 60 mg/kg group 13, 1 and 1 respectively left. The pooled estimate has 5% for light in both the 40 mg/kg and 60 mg/kg groups and 1% (40 mg/kg) and 0.2% (60 mg/kg) for moderate intensity. There was no significant difference between the treatment groups.

### 6-month and 12-month follow-up reinfection rates

The product-limit estimate of the time to reinfection was calculated starting from Day 21 restricted to the patients who were free of parasites throughout Day 360 (12 months).

**Figure 5:**
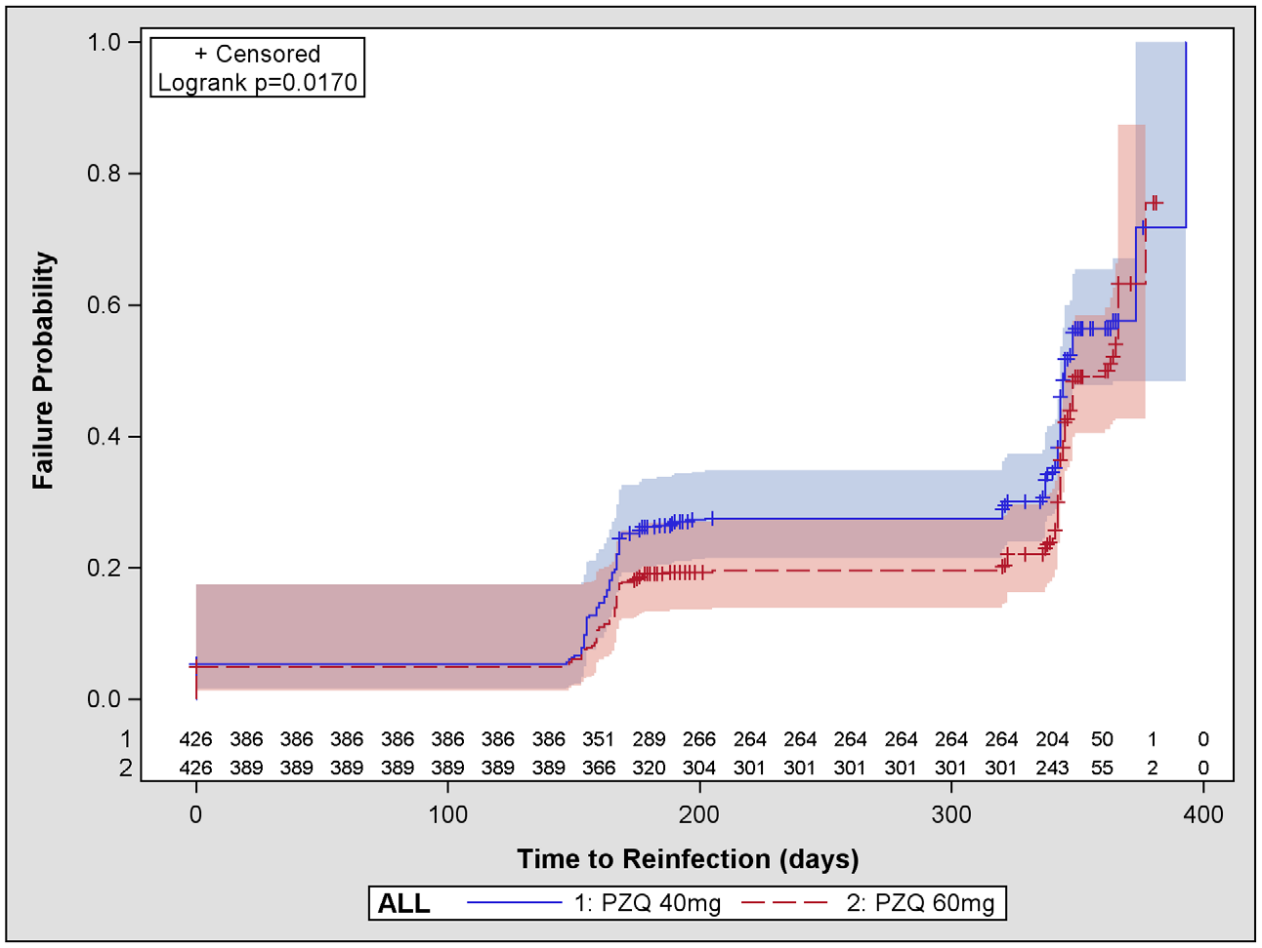
Infection-free survival curves. Kaplan-Meier infection-free survival curves for all sites combined.

Table S1 presents mean epg comparisons between groups and over time. The median infection-free survival and reinfection rates for each study site are presented as an annex (Table S2). The Kaplan-Meier curves for the global estimate are presented in Figure 5.

Reinfection rates were lowest in Mauritania (8% and 3% in the 40 and 60 mg/kg arms respectively) and highest in Tanzania (47% and 37% respectively). The median infection-free survival was similar between sites (though this may be an artefact as patients were seen only twice during follow-up: around Day 180 and Day 360). For the comparison between the two doses, the hazard ratio (HR) was not significant in all countries except Brazil in favour of 60 mg/kg, and was significant on the pooled estimates (pooled reinfection rates: 34.3% (95%CI = [29.8; 39.3]) in the 40 mg/kg arm and 23.9% (95%CI = [20.0; 28.4]) in the 60 mg/kg arm; HR = 0.78, 95%CI = [0.63;0.96])

**Figure 6:**
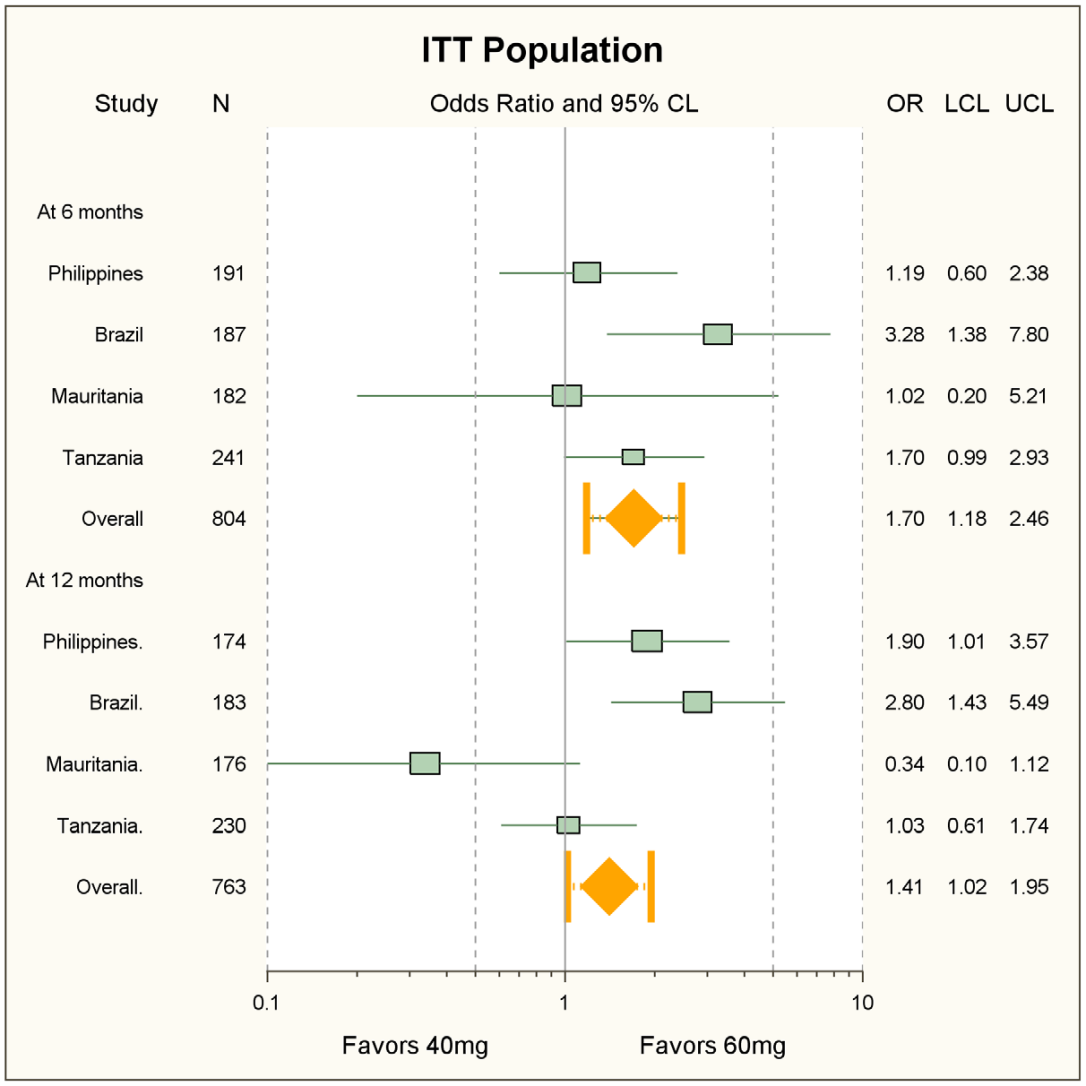
Reinfection rates at 180 and 360 days of follow-up. Forest plot of reinfection rates with 40 and 60 mg/kg at 180 and 360 days of follow-up.

In addition to the Kaplan-Meier estimates presented above, we also compared the reinfection rates occurring at 6 and 12 months of follow-up for each individual country as well as for the pooled data (Figure 6). The pooled OR(95%CIs) were 1.70 (1.18, 2.46, p = 0.0047) at 6 months and 1.41 (1.02, 1.95, p = 0.0037) at 12 months, both showing a difference in favour of the 60 mg/kg group. The 76% I^2^ on Day 360 is explained by the OR in Mauritania being in favour of the 40 mg/kg dose. Overall, 40% of the 388 total recrudescences occurred at 6 months. The difference was significant on both occasions in Brazil, and borderline in Tanzania at 6 months and the Philippines at 12 months.

Intensity of infection on day 180 raised again in all countries as intensity was at least light in >20–25% of the patients in the Philippines, Brazil and Tanzania. In Mauritania intensity was moderate or heavy in 3% of the patients. The pooled estimate had 22.8% for light, 3.4% moderate and 1.3% for heavy intensity in the 40 mg/kg group and 12.6% for light, 3.2% moderate and 1.6% for heavy intensity in the 60 mg/kg groups (Table S3).

At Day 360 in the Philippines the intensity of infection was more pronounced. While, compared to Day 180, the proportion of light intensity infections remained stable in all countries except in Tanzania (where it increased dramatically), there were more moderate intensity infection (9% vs 13% in the Philippines, 3% vs 6% in Brazil, 2% vs 3% in Mauritania and 8% vs 14% in Tanzania). The pooled estimate has 30% vs 23% for light intensity in the 40 mg/kg vs. 60 mg/kg groups, 8% vs. 7% for moderate intensity and 3% vs. 3% for heavy intensity.

There was no significant difference between the treatment groups in the intensity classes on either Day 180 or Day 360.

### Safety analysis

#### Frequency of AEs

**Figure 7:**
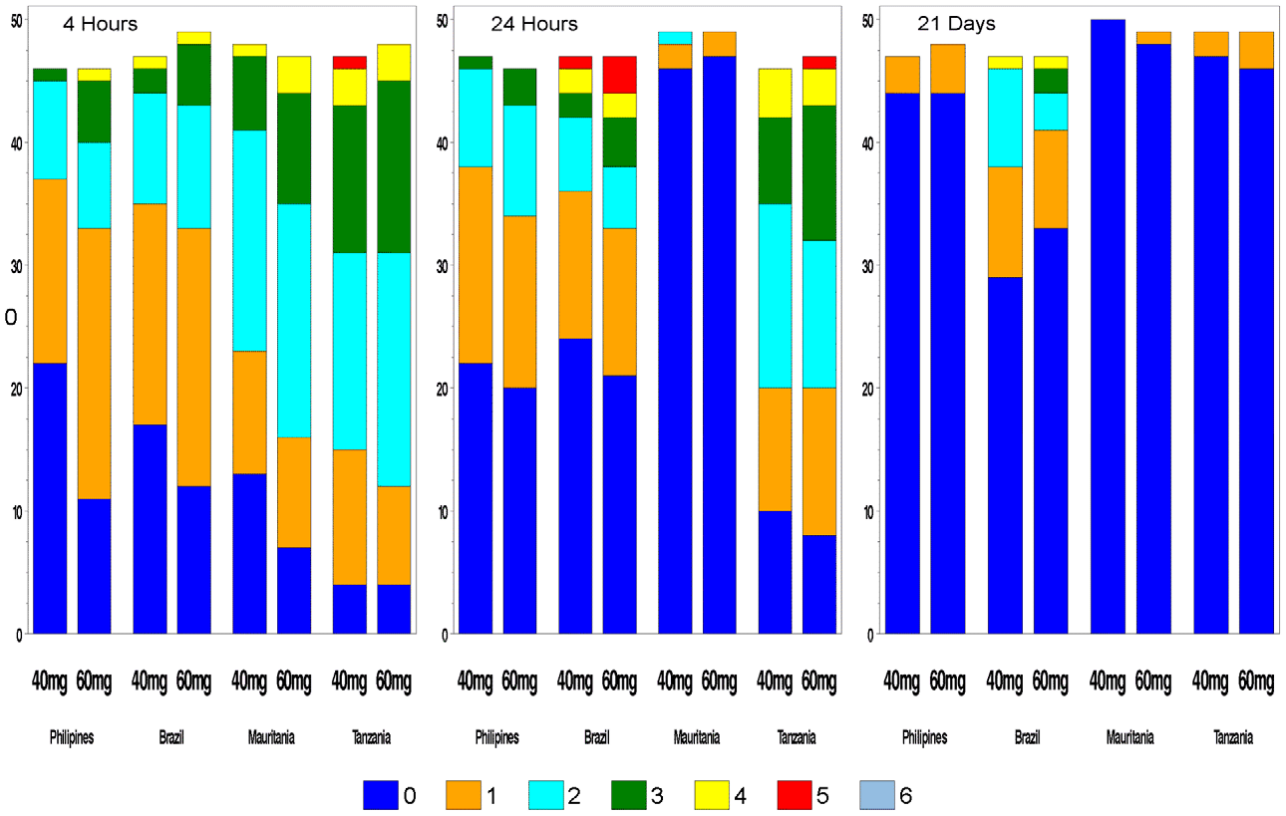
Adverse events at 4 hours, 24 hours and 21 days. Number of adverse events (AEs) by treatment group at 4 hours, 24 hours and 21 days post-dosing.

The number of patients with AEs and the number of AEs decreased over time (Figure 7). These results reflect the fact that the definition of AE includes also signs and symptoms of the disease itself.

Overall 666 patients (78%) reported a total of 1327 AEs 4 hours post-dosing (Table S4). The risk of having at least one AE was higher in the 60 than in the 40 mg/kg group (83% vs. 73%, OR 0.54, 95%CI 0.39–0.76, Chi-square p-value<0.001). The proportion of patients with AEs varied across sites from 66% in the Philippines to 92% in Tanzania. (Table 3)

**Table 3:** Most frequent AEs occurring with >2% frequency at any visit by treatment group and intensity.

		40 mg/kg						60 mg/kg						All					
		Mild		Moderate		Severe		Mild		Moderate		Severe				40		60	
		N	%	N	%	N	%	N	%	N	%	N	%	N	%	N	%	N	%
4 Hours	ABDOMINAL PAIN	170	(64.89%)	90	(34.35%)	2	(0.76%)	147	(53.65%)	125	(45.62%)	2	(0.73%)	536	(40.39%)	262	(41.65%)	274	(39.26%)
	DiARHOEA	83	(72.81%)	30	(26.32%)	1	(0.88%)	83	(70.34%)	30	(25.42%)	5	(4.24%)	232	(17.48%)	114	(18.12%)	118	(16.91%)
	HEADACHE	59	(80.82%)	14	(19.18%)			55	(79.71%)	14	(20.29%)			142	(10.70%)	73	(11.61%)	69	(9.89%)
	NAUSEA	49	(80.33%)	12	(19.67%)			56	(80.00%)	14	(20.00%)			131	(9.87%)	61	(9.70%)	70	(10.03%)
	VOMiTiNG	31	(77.50%)	8	(20.00%)	1	(2.50%)	61	(76.25%)	18	(22.50%)	1	(1.25%)	120	(9.04%)	40	(6.36%)	80	(11.46%)
	DiZZiNESS	45	(88.24%)	5	(9.80%)	1	(1.96%)	50	(90.91%)	5	(9.09%)			106	(7.99%)	51	(8.11%)	55	(7.88%)
	ALLERGY	9	(75.00%)	2	(16.67%)	1	(8.33%)	9	(64.29%)	4	(28.57%)	1	(7.14%)	26	(1.96%)	12	(1.91%)	14	(2.01%)
	ANOREXiA	2	(100.00%)					1	(50.00%)	1	(50.00%)			4	(0.30%)	2	(0.32%)	2	(0.29%)
	FEVER							3	(100.00%)					3	(0.23%)	0	(0.00%)	3	(0.43%)
	ALL	457		177		10		474		227		13		1327		629		698	
24 Hours	ABDOMINAL PAIN	84	(73.68%)	30	(26.32%)			78	(70.91%)	32	(29.09%)			224	(24.40%)	114	(35.63%)	110	(34.38%)
	DiARHOEA	29	(55.77%)	21	(40.38%)	2	(3.85%)	34	(51.52%)	27	(40.91%)	5	(7.58%)	118	(12.85%)	52	(16.25%)	66	(20.63%)
	HEADACHE	81	(80.20%)	20	(19.80%)			84	(82.35%)	18	(17.65%)			203	(22.11%)	101	(31.56%)	102	(31.88%)
	NAUSEA	28	(87.50%)	4	(12.50%)			25	(80.65%)	6	(19.35%)			63	(6.86%)	32	(10.00%)	31	(9.69%)
	VOMiTiNG	12	(85.71%)	2	(14.29%)			16	(66.67%)	8	(33.33%)			38	(4.14%)	14	(4.38%)	24	(7.50%)
	DiZZiNESS	36	(97.30%)	1	(2.70%)			38	(92.68%)	3	(7.32%)			78	(8.50%)	37	(11.56%)	41	(12.81%)
	ALLERGY	28	(73.68%)	8	(21.05%)	2	(5.26%)	22	(53.66%)	15	(36.59%)	4	(9.76%)	79	(8.61%)	38	(11.88%)	41	(12.81%)
	ANOREXiA	8	(50.00%)	7	(43.75%)	1	(6.25%)	7	(50.00%)	7	(50.00%)			30	(3.27%)	16	(5.00%)	14	(4.38%)
	FEVER	14	(93.33%)	1	(6.67%)			15	(93.75%)	1	(6.25%)			31	(3.38%)	15	(4.69%)	16	(5.00%)
	ALL	342		99		5		337		125		10		918		446		472	
21 Days	ABDOMINAL DISTENSION	1	(100.00%)											1	(0.53%)	1	(1.03%)	0	(0.00%)
	DiARHOEA	3	(60.00%)	2	(40.00%)			5	(83.33%)	1	(16.67%)			11	(5.82%)	5	(5.15%)	6	(6.52%)
	HEADACHE	13	(54.17%)	11	(45.83%)			13	(50.00%)	13	(50.00%)			50	(26.46%)	24	(24.74%)	26	(28.26%)
	NAUSEA	4	(100.00%)					5	(83.33%)	1	(16.67%)			10	(5.29%)	4	(4.12%)	6	(6.52%)
	VOMiTiNG	3	(75.00%)	1	(25.00%)					2	(100.00%)			6	(3.17%)	4	(4.12%)	2	(2.17%)
	DiZZiNESS	11	(91.67%)	1	(8.33%)			10	(100.00%)					22	(11.64%)	12	(12.37%)	10	(10.87%)
	ALLERGY	5	(100.00%)					1	(100.00%)					6	(3.17%)	5	(5.15%)	1	(1.09%)
	ANOREXiA	1	(10.00%)	9	(90.00%)			5	(35.71%)	9	(64.29%)			24	(12.70%)	10	(10.31%)	14	(15.22%)
	FEVER	3	(60.00%)	2	(40.00%)			5	(83.33%)	1	(16.67%)			11	(5.82%)	5	(5.15%)	6	(6.52%)
	ALL	70		27				59		33				189		97		92	

AE: adverse events.

At 21 hours post-dosing, 456 patients (54%) reported 918 AEs. There was no difference between the two doses (OR 0.92, 95%CI 0.68–1.17), p = 0.4).

At Day 21, 114 patients (13%) had 189 AEs with no difference between the groups.

#### Type and severity of AEs

The most frequent AEs (those occurring with >2% frequency at any visit between 4 hours, 24 hours and 21 days) are summarized in Table 3 by dose and severity. The most frequent AE was abdominal pain at both 4 and 24 hours (40% and 24% respectively of all AEs at either visit). Other AEs that contributed >10% on both visits were diarrhoea and headache.

At 4 hours, regarding the most prevalent AEs, respectively in the 40 and 60 mg/kg group, severe intensity was reported for abdominal pain (1% (n = 2) and 1% (n = 2)), allergy (8% (n = 1) and 7% (n = 1)), diarrhoea (1% (n = 1) and 4% (n = 5)), dizziness (2% (n = 1) and 0%), and vomiting (2% (n = 1) and 1% (n = 1)). Moderate intensity were also reported for abdominal pain (34% (n = 90) and 46% (n = 125)), allergy (17% (n = 2) and 29% (n = 4)), diarrhoea (26% (n = 30) and 25% (n = 30)), dizziness (10% (n = 5) and 9% (n = 5)), headache (19% (n = 14) and 20% (n = 14)), nausea (20% (n = 12) and 20% (n = 14)) and vomiting (20% (n = 8) and 22% (n = 18)).

At 24 hours, respectively in the 40 and 60 mg/kg, severe intensity was reported for allergy (5% (n = 2) and 10% (n = 4)), anorexia (6% (n = 1) and 0%), chest tightness (0% and 17% (n = 1/6)) and diarrhoea (4% (n = 2) and 8% (n = 5)). Moderate intensities were observed for abdominal pain (26% (n = 30) and 29% (n = 32)), allergy (21% (n = 8) and 37% (n = 15)), anorexia (44% (n = 7) and 50% (n = 7)) diarrhoea (40% (n = 21) and 41%(n = 27)), dizziness (3% (n = 1) and 7% (n = 3)), fever (7% (n = 1) and 6% (n = 1)) headache (20% (n = 20) and 18% (n = 18)), nausea (12% (n = 4) and 19% (n = 6)), and vomiting (14% (n = 2) and 33% (n = 8)).

At Day 21, there was no severe AE.

Only one event (abdominal pain in Mauritania at 4 hours) was reported as serious.

#### Drug-event relationship for AEs

At 4 hours post-dosing, of the most common AEs occurring in the 40 mg/kg group, the event was judged to be as “most probably” or “probably” related to the drug in 19% and 25% of cases for abdominal pain, 16% and 29% for diarrhoea, 10% and 24% for dizziness, 2% and 23% for headache, and 8% and 19% for vomiting. Most probable and probable relationships to treatment in the 60 mg/kg group were also reported for abdominal pain (21% and 25%), diarrhoea (12% and 34%), dizziness (5% and 31%), headache (1% and 21%), nausea (27% and 22%) and vomiting (18% and 36%).

At 24 hours, in the 40 mg/kg group events judged to be “most probably” and “probably” related to the drug were for abdominal pain 26% and 55%, diarrhoea 12% and 72%, dizziness 2% and 34%, headache 2% and 39% and vomiting 7% and 64%. Most probable and probable relationships to treatment in the 60 mg/kg group were also reported for abdominal pain (25% and 58%), diarrhoea (20% and 79%), dizziness (2% and 34%), headache (2% and 41%), nausea (19% and 55%) and vomiting (4% and 46%).

On Day 21 one case of dizziness in Brazil and three of chest tightness in Tanzania were judged to be probably related to treatment.

## Discussion

This study shows that a higher dose of 60 mg/kg of praziquantel offers no significant efficacy advantage over standard 40 mg/kg for treating intestinal schistosomiasis caused by either *S. mansoni* or *S. japonicum* when assessed three weeks (21 Days) post-dosing. With 40 mg/kg of praziquantel cure rates are >91% at all sites except Tanzania (87–88%) and egg reduction rates are >89% by Day 21. During long-term follow-up at 6 and 12 months, reinfection rates were higher in the group receiving 40 mg/kg. Patients treated with 60 mg/kg had a higher risk of adverse events occurring immediately post-dosing (4 hours), while no difference was seen 24 hours or 21 days later. The results presented are based on the intent-to-treat population and are confirmed by other analyses on per-protocol population as well as various sensitivity analyses. While the sites differed in terms of transmission and infection intensities, there was no bias or heterogeneity across sites for the main efficacy outcomes except reinfection rates at one year of follow-up.

Together, these results support the current WHO recommendation of deploying praziquantel at 40 mg/kg.

These studies were conducted to respond to the demand for evidence about the correct dose of praziquantel when used by control programmes in endemic countries. The plan was to have a multicentre study with a master protocol whereby each site will follow the same protocol and be powered to show a difference between expected cure rates, and to have results combined prospectively for an individual patient meta-analysis.

Prevalence of screening of intestinal schistosomiasis infection ranged 25% (Brazil)–57% (Mauritania) and the rate of reinfection one year post-treatment ranged from 9% (Mauritania) to 44% (Tanzania) between the two treatment arms. Of all sites Tanzania had the most intense infections, the lowest cure rates (though with high egg reduction rates), and the highest reinfection rates - all testifying high transmission intensity.

Reinfections were more frequent in the 40 than in the 60 mg/kg group at each site, but the difference was significant only for Brazil and the pooled Kaplan-Meier analysis. The difference between the two arms was already significant on aggregate and for Brazil by Day 180, when 40% of reinfections had occurred. However, there was no difference between the two groups in terms of intensity classes. It is difficult to explain how a single dose of praziquantel could affect the risk of reinfection in the following 12 months. Indeed the difference could be a random effect. However, the Kato-Katz method has been reported to overestimate cure rates [8] (although here two duplicates were done on consecutive days were used) and it is hence possible that it failed to identify low egg counts on Day 21; the cases identified would then result from a mixture of undetected failures and true reinfections.

Praziquantel treatment of either urinary or intestinal schistosomiasis is known to modify both humoral and cellular responses (like age does). (see for instance Mutapi et al, 2003 [9]). It remains to be investigated whether the higher dose could elicit a greater post-treatment antibody and cytokine shift.

The dose-plasma concentration relationship of praziquantel is poorly know. A study conducted in healthy adult volunteers with 5, 10, 20, 50 mg/kg showed with increasing doses (i) higher exposure (exponential increase of C_max_, AUC_0–24_); (ii) more rapid absorption (significant reduction of T_max_); (iii) slightly reduced t½ [10]. However, no study in the target population (infected school-age children) has produced pharmacokinetic and pharmacokinetic/pharmacodynamic information on dose-concentration and dose-effects.

Based on data from the above-mentioned study in volunteers the doses of 40 and 60 mg/kg (neither have data in the above study) would be expected to produce AUC_0–24_ (ng*h/mL) of 2900 and 4600 or 2100 and 8600, respectively depending on whether a linear or exponential relationship applies - meaning that the AUC obtained with a dose of 60 mg/kg (1.5 times higher) would be 1.6 or 4 times higher than with 40 mg/kg respectively. For the 40 mg/kg dose, this is coherent with other studies: for comparison, a dose of 40 mg/kg produced AUC_0–24_ ranging 2,110-4.098 ng*h/mL when given in fasting conditions to adult healthy volunteers, but was ∼4 times higher (15,928 ng*h/mL) in patients [11]. However, without drug levels this remains in the realm of speculation. In addition, bioavailability (and thus exposure) is known to have high inter-individual variability, and to change between an empty stomach and with food (and the type of food: higher with carbohydrates than fat [12]) as well as the brand of the product and between health and disease. Finally, there are no data at present to correlate exposure and effects for praziquantel. It is unfortunate that this study could not collect blood samples for drug level determination. In any case, the higher exposure expected with 60 mg/kg did not translate in a sizeable efficacy advantage in this study but may entail a higher risk of toxicity (at least when given as a single dose) although adverse events were generally mild and transient.

A limitation of this trial is that it was designed with a superiority hypothesis expecting the 60 and 40 mg/kg doses to be 80% and 60% effective at each site. This assumption (which was based on the recommendations made at the 1991 WHO expert committee [5]) was disproved by the finding that the cure rates ranged 88–97% (92.8% on aggregate) with 60 mg/kg and 87–98% (91.7% on aggregate) with 40 mg/kg. A non-inferiority trial design would have been more appropriate. The question is whether with such design the current trial would support the non-inferiority of 40 mg/kg with respect to 60 mg/kg. Assuming the cure rate of the reference treatment (60 mg/kg) to be 93% and accepting a difference (δ) of 6%, with a precision (α) = 0.015 and a power (1-ß) = 90% a study enrolling 428 patients per arm with cure rates of 91.7% and 92.8% (difference = 1.1, 95%CI −3.63, +5.75) would be within the 6% δ margins and thus support the non-inferiority of the 40 vs. 60 mg/kg dose.

Another issue is with the safety evaluation. Here, any event occurring after drug intake (starting 4 hours after administration) was conservatively reported as an adverse event (AE), and their incidence, type and severity compared between treatment groups. However, the presence and grading before drug administration was not recorded - it is thus not possible to describe the treatment-emergent signs and symptoms (i.e. those that were not present pre-treatment or worsened with the treatment), and to differentiate between the events related to the disease and those that may be caused by the drug. Tanzania reported more events than the other sites - which may be related also to the fact that the Tanzanian patients were more heavily infected than the others.

The results of this study, along with those of systematic reviews, should be used to inform policy decisions in the countries [5]. Single dose treatment ensures high population compliance with treatment which would not be possible with spaced doses of 40 mk/kg praziquantel [6]. The Philippines has already changed from 60 to 40 mg/kg after considering the local results [13].

Reliable, up-to-date evidence is needed for policy decisions. As mentioned above, a yet unpublished Cochrane systematic review of treatments of schistosomiasis mansoni found a dose-effect in the cure rates of praziquantel up to 40 mg/kg and no gain beyond this dose. In contrast, no dose-effect was detected in schistosomiasis haematobia [6]. Of note, only 3 of the 10 praziquantel studies of the urinary and 8 the 20 of the mansoni review had been conducted in the past 30 years - all the others were older. One single treatment policy is practical but may not fit all cases. For areas where both intestinal and urinary schistosomiasis coexist the dose of 40 mg/kg is expected to cure both. In an area of Egypt where schistosome parasites were thought to be less responsive to a 40 mg/kg dose of praziquantel, no increase in drug failures was noted following 10 years of drug pressure at this dosage [14]. With the lower dose vigilance is required to ensure that treatment failures and possible resistance can be detected early, particularly for special cases (such as areas where praziquantel efficacy is reportedly suboptimal). There is also a need for information on exposure post-dosing (drug levels) in target populations (esp. school-age children) and how it correlates with efficacy and safety.

## Supporting Information

Checklist S1 **Consort checklist.**
(DOC)

Figure S1 **Funnel plot of log[OR] of adjusted cure rates against SE(log[OR]).** Funnel plot of log[OR] of adjusted cure rates against SE(log[OR]).
(TIF)

Protocol S1 **Protocol of Tanzania.**
(PDF)

Protocol S2 **Protocol of the Philippines**
(PDF)

Protocol S3 **Protocol of Mauritania.**
(PDF)

Protocol S4 **Protocol of Brazil.**
(PDF)

Table S1 **Eggs per gram at Day 180 and Day 360.**
(DOC)

Table S2 **Post-day 21 reinfections.**
(DOC)

Table S3 **Intensity of infection at Day 180 and Day 360.**
(DOC)

Table S4 **Summary of safety findings at Day 0, 4 hours post-dosing.**
(DOC)
