# Supplementary figures and images for: A Multicentre Randomized Controlled Trial of the Efficacy and Safety of Single-Dose Praziquantel at 40 mg/kg vs. 60 mg/kg for Treating Intestinal Schistosomiasis in the Philippines, Mauritania, Tanzania and Brazil
Source: PLoS Negl Trop Dis. 2011 Jun 14;5(6):e1165. doi: 10.1371/journal.pntd.0001165 (PMC3114749; doi:10.1371/journal.pntd.0001165)

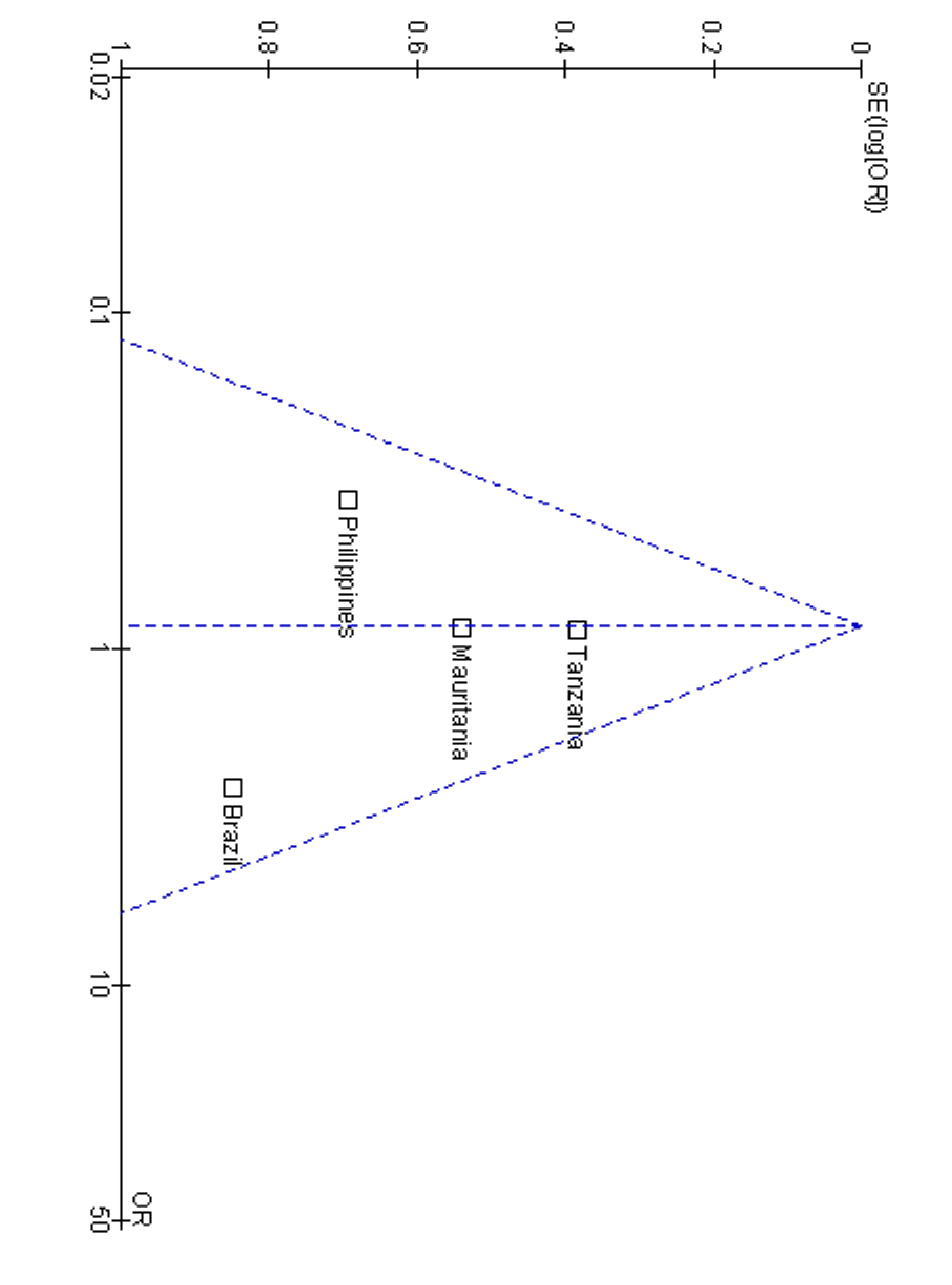

Supplement: Figure S1 — Funnel plot of log[OR] of adjusted cure rates against SE(log[OR]). Funnel plot of log[OR] of adjusted cure rates against SE(log[OR]). (TIF) [file pntd.0001165.s002.tif]
