# Supplementary material for: A Multicentre Randomized Controlled Trial of the Efficacy and Safety of Single-Dose Praziquantel at 40 mg/kg vs. 60 mg/kg for Treating Intestinal Schistosomiasis in the Philippines, Mauritania, Tanzania and Brazil
Source: PLoS Negl Trop Dis. 2011 Jun 14;5(6):e1165. doi: 10.1371/journal.pntd.0001165 (PMC3114749; doi:10.1371/journal.pntd.0001165)
Supplement: Protocol S1 — Protocol of Tanzania. (PDF) [file pntd.0001165.s003.pdf]

PROJECT DESCRIPTION

TITLE: Efficacy and safety of increased dosage of Praziquantel (60mg/Kg) in the treatment of schistosomiasis

PI: Nicholas LWAMBO

1. INTRODUCTION

Schistosomiasis is currently estimated to infect about 200 million people World-wide, and 85% of them are believed to be in sub-Saharan Africa. It is ranked second to malaria in terms of extent of endemic areas and number of infected people. Several control options have been previously advocated, however, our better understanding of the epidemiology of the infection and disease, the advent of safe, efficacious, single oral dose anti-schistosomal drugs point to the strategy of morbidity reduction by chemotherapy as the mainstay of schistosomiasis morbidity control (WHO, 2002). Praziquantel (PZQ) is currently a drug of choice for the treatment of schistosomiasis because of its high effectiveness against the adult stage of all human schistosomiasis species, its ease of administering (single oral dose) and its good safety profile with usually mild adverse side effects (Kumar, et al, 1994). Moreover, the price of PZQ has recently been substantially reduced (WHO, 2002) making it more available and affordable for most people in endemic countries.

Praziquantel is an isoquinoline-pyrazine derivative (2-cyclohexylcarbonyl-1,3,4,6,7,11b-hexahydro-2H-pyrazino (2,1-a)isoquinoline-4-one). It has a broad antiparasitic effect and first marketed as a veterinary taeniacide under the trade name Droncit (Bayer) (MacMahon &Kolstrup (1979). Clinical studies in man have shown that it is effective in human tapeworm infestation and all human schistosome infections. The recommended dosage of PZQ is 40mg/kg body weight given orally as a single dose (WHO, 1993), although higher doses of up to 75mg/kg have been given and found to be rapidly absorbed, metabolized and well tolerated. The mere fact that PZQ is given as a single dose improves compliance compared to earlier products (antischistosomal drugs such as amblihar and metrifonate) which required multiple administrations lasting over several days or weeks.

In Tanzania PZQ at 40mg/kg body weight as a single dose has been tested against infections by *S haematobium* (McMahon & Kolstrup, 1979) and *S mansoni* (Rugemalila et al., 1984). McMahon & Kolstrup (1979) recorded high cure rates of 83%, 76% and 7I% against *S haematobium* at 1- month, 2-months and 6- months, respectively. In *S mansoni*, however, Rugemalila et al. (1984) observed lower cure rates of 35%, 56% and 63% at 1-month, 2- months and 6- months, respectively.

The unusually low cure rates reported by Stelma et al. (1995) in Senegal, while working in very intense focus of *S. mansoni*, has raised much concern about the emergency of resistance or tolerance of schistosomes to PZQ. So in order to circumvent this problem, several strategies to reduce the rate of schistosome species developing resistance against PZQ and to optimize the use of PZQ in schistosomiasis control has been suggested. In the present study, the suggested option to be explored is to increase the dose regimens of PZQ up to 60mg/kg body weight.

Stelma et al. (1997) increased the dose of PZQ from standard dosage of 40mg/kg to 60mg/kg and showed to have no significant improvement in efficacy. Van Lieshutt et al. (1994), however, showed that the opposite was true. This controversy requires to be urgently resolved by collecting data on the safety, efficacy and effectiveness of increased doses of PZQ in different epidemiological setting where schistosomiasis is highly endemic.

Children are especially vulnerable to schistosomiasis, and infected school-age children are often physically and intellectually compromised by concurrent anemia, attention deficits, learning disabilities, school absenteeism and higher dropout rates (Guyatt et al., 2001; WHO, 2002). Schistosomiasis Control Programs target school-aged children, aiming for clinical cure and morbidity reduction. Many previous trials were done with children and adolescents to test the efficacy of praziquantel alone or in combination with other drugs (Jaoko et al., 1996; Guisse et al., 1997; Olds et al., 1999; Urtzinger et al., 2000; de Clercq et al., 2002). Since praziquantel is a safe and well known drug (as described above) and the improvement of treatment should be achieved for those who need the most, children and adolescents will be the chosen study population for this trial.

2. OBJECTIVES

The main objective of this study is to determine if higher dose of praziquantel at 60 mg/kg will improve treatment efficacy with an adequate safety profile, in comparison with standard regimen of praziquantel at 40mg/kg.

Specifically:
2.1 To determine cure rate in increased dosage regimen of PZQ 60 mg/kg in comparison with a standard dosage of 40mg/kg of PZQ in the treatment of *S. mansoni*
2.2 To determine egg reduction rate in increased dosage regimen of PZQ 60 mg/kg in comparison with a standard dosage of 40mg/kg of PZQ in the treatment of *S. mansoni*
2.3 To determine adverse reactions (events) in increased dosage regimen of PZQ 60 mg/kg in comparison with a standard dosage of 40mg/kg of PZQ in the treatment of *S. mansoni*
2.4 To determine morbidity (haemoglobin, weight) in increased dosage regimen of PZQ 60 mg/kg in comparison with a standard dosage of 40mg/kg of PZQ in the treatment of *S. mansoni*

3. EXPERIMENTAL DESIGN AND METHODS

The trial described below will be conducted in compliance with the protocol, Good Clinical Practice and applicable regulatory requirements.

3.1. *Study site.* The study is to be done in Mwanza Region, Tanzania, where both *S. mansoni* and *S. haematobium* are highly endemic particularly in Magu and Misungwi districts. In a recent survey of schoolchildren by Lwambo, *et al* (1999) in the area the prevalence of urinary schistosomiasis was found to be 56.5% (range 13.6-87.7%) and 10.9 % (range 0.0-64.4%) for intestinal schistosomiasis. *S mansoni* is more prevalent along the Lake Victoria shore, while *S haematobium* is more prevalent in the hinterland, away from the lakeshore. In this area mixed *S haematobium/ S mansoni* infections have a low prevalence of 4.0 % (range 0.0 – 21.0 %). The study will be done in schools reported by Lwambo *et al*. (1999) to have high prevalence of *S mansoni*, i.e., Mayega 64.7%, Kigangama 53.3%, Lutale 49.5%, Kongolo 41.7%, Chumve 41.5%, Kabita 39.8%, Ihale 38.0% and Ilumwa 34.4%. Also schools located in the same area as the high prevalence schools may be considered for baseline screening. Lwambo (1988) have shown that peak transmission of *S haematobium* in the area occurs during the short dry period, sometime in February/March so that most of the infections in the community would be detected in April/May. Magendantz (1972) showed that the transmission of *S mansoni* occurs mainly in Lake Victoria throughout the year.

3.2. *Study design.* The proposed study is a double-blind, randomized controlled trial to determine if higher doses of praziquantel (PZQ 60mg/kg) will improve treatment efficacy of *S mansoni* with an adequate safety profile, in comparison with standard regimen of PZQ 40mg/kg. The trial is to be conducted with schoolchildren aged between 10 to 19 years old. This age group is chosen because it is

school age children who are heavily infected by schistosomes, therefore, it is the group in which the new increased dosage of PZQ is anticipated to be used. The potential beneficiary of increased efficacy of PZQ is school-age children and, therefore, it would be prudent to determine the safety profile of the new dosage regimen in the same age group.

The following are the study endpoints:

- Primary endpoint:
  Cure rate at 21 days after treatment for the PZQ 60mg/Kg and PZQ 40mg/Kg groups.
- Secondary endpoints:
  a) Egg reduction rate at 21 days after treatment for the PZQ 60mg/Kg and PZQ 40mg/Kg groups.
  b) Comparison of frequency and severity of adverse events during the 4 hours after treatment, 24 hours and 21 days, for the PZQ 60mg/Kg and PZQ 40mg/Kg groups.
  c) Comparison of the variation of hemoglobin (Hb) level at 21 days, 6 months and 12 months after treatment (as related to the baseline measurement), for the PZQ 60mg/Kg and PZQ 40mg/Kg groups.
  d) Comparison of the variation of weight at 6 months and 12 months after treatment (as related to the baseline measurement), for the PZQ 60mg/Kg and PZQ 40mg/Kg groups.

The flow-chart below illustrates the study design:

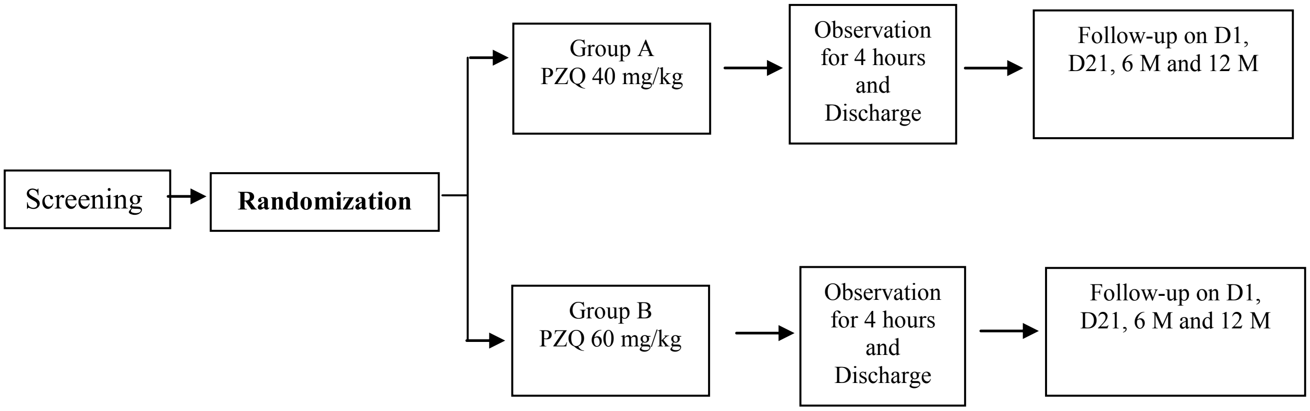

Rapport with government and community leaders at district, ward, village and school levels will be established in the study area. Informed Consent will be obtained for each participant. For children considered minors written Informed Consent will be obtained from their guardians. Schoolchildren meeting the screening criteria will be recruited continuously, subjects will be examined quantitatively for *S mansoni* ova and semi-quantitatively for *S haematobium.* Two stool samples (2 Kato Katz slides will be prepared from each sample) and one urine sample will be collected within 5 days. Those who fulfill the inclusion and exclusion criteria for enrolment in the trial will be randomized to receive treatment with PZQ within 7 days (+/-1 day).

See Appendix 1for the schedule of activities. It is estimated that 800 school children will be examined during the baseline study within one month. It is expected that about 240 children (30%) will fulfill the criteria for enrollment in the trial. Participants with positive *Schistosoma spp.* diagnosis who do not fulfill the inclusion and exclusion criteria will be treated with the standard treatment of a single dose of PZQ 40mg/Kg.

Schoolchildren in the treatment groups will be followed up for a total of 12 months post-treatment.

The following baseline screening and follow up visits will be done. As shown in Appendix 1 the baseline study is scheduled to last 23 days. Since enrollment is done continuously, activities of D0 will start on the 8th day after screening and activities of D1 on the 9th day and run concurrently with the screening.
Two teams will be required during the screening period, one to do the baseline screening to identify eligible children for the clinical trial. The other (composed of at least one physician) will examine children for inclusion and exclusion criteria, recruit and treat them with PZQ on D0 and assess them for adverse drug events 4 hours after treatment on D0, on D1 and D21. In addition, on D21 cure rate and egg reduction rate will be determined. At six months (6M) and 12 months (12M) after treatment, trial subjects in the two groups will be followed up for determination of cure rate, egg reduction rate, weight and Hb. Here is a detailed account of the activities to be undertaken at D0, D1, D21, 6M and 12M, their timings (with ranges) and the assessments to be made.

D0 (means 7 days after baseline study +/-1 day)
Clinical history of each schoolchild fulfilling screening criteria using questionnaire will be taken to see if the subject has acute or chronic severe disease, including hepato-splenic schistosomiasis. In addition, the questionnaire will seek to establish a history of adverse reactions associated with PZQ, a history of treatment with PZQ within 30 days and medication (such as antibiotics) currently being used which may affect the results of the present trial. This will be followed by a thorough physical examination by a physician for physical fitness to participate in the trial. Those showing signs and symptoms of clinical malaria will be referred for treatment and excluded from the study and referred for treatment. Childbearing age female subjects will further be tested for pregnancy using the Quickstick™ One step HCG Pregnancy Test. Those tested positive for pregnancy will be excluded from the study. Each girl will be given individual counseling from the study nurse. Those children with schistosomiasis who will be excluded from the study at this point will be treated with a single oral dose of PZQ at 40mg/kg.
For the children who fulfill all criteria for enrolment, body weight will be determined and a finger-prick blood sample will be collected for Hb measurement. Thereafter they will be treated with PZQ according to weight and dosage regimen assigned in the trial (see randomization and blinding section for treatment assignment). During the 4 hours post-treatment study subjects will be observed for occurrence of adverse events and by the end of this period they will be assessed for adverse events using a questionnaire.

D1 (means 24 hours after treatment with PZQ 24hs +/- 2 hours).
The physician in the research team will assess schoolchildren treated on D0 for adverse events using a questionnaire.

D21 (means 21 days after treatment +/- 2 days)
The physician in the research team will assess schoolchildren treated on D0 for adverse events using a questionnaire. In addition, Hb will be determined in all schoolchildren in the trial. Stool samples will be collected on two separate days and each day duplicate Kato Katz slides will be made for quantitative examination of *S mansoni* ova. This will allow cure rate and egg reduction rate at D21 to be calculated.

6M (means 180 days after treatment +/- 7 days)
At 6M follow up cure rate, egg reduction rate, weight and Hb will be determined in all study subjects in the trial. Therefore, stool samples will be collected on two separate days and each day duplicate Kato slides will be made for quantitative examination of *S mansoni* ova. This will allow cure rate and egg reduction rate at 6M to be calculated.

12M (means 365 days after treatment +/- 14 days)

At this follow up activities conducted at 6M will be repeated, that is, cure rate, egg reduction rate, weight and Hb will be determined in all study subjects in the trial. Therefore, stool samples will be collected on two separate days and each day duplicate Kato slides will be made for quantitative examination of *S mansoni* ova. This will allow cure rate and egg reduction rate at 12M to be calculated.

Table 1. A summary of study procedures are presented below for each visit:

	Screening	Day 0	Day1	Day 21	Month 6	Month 12
Clinical history	X	X				
Inclusion/Exclusion criteria*	X	X				
Cure rate				X	X	X
Egg Reduction Rate				X	X	X
Adverse events		X	X	X		
Weight		X			X	X
Hb		X		X	X	X

* Screening for exclusion criteria includes urine test for pregnancy (childbearing age female participants) and diagnosis of *S. haematobium*.

3.3. *Randomization:*

Schoolchildren with *S mansoni* and fulfilling both inclusion and exclusion criteria will be randomly assigned to two groups: one group will receive PZQ 40mg/kg and the other group PZQ 60mg/kg.

Randomization will be generated in blocks of 4, in a ratio of 1:1 for each drug regimen. Sealed and numbered envelopes containing the treatment regimen for each sequential participant to be enrolled will be provided from TDR. The envelopes containing the codes will be kept in a locked cabinet and one designated person of the team will be responsible for them. This same person will be responsible for opening the envelope and verifying the corresponding treatment once a new participant is enrolled in the study. Each participant will be assigned a unique Study I.D. number (ID#). This ID# will be assigned in sequential order and will be recorded in the Screening Log. The treatment will be provided to the participant according to the Praziquantel regimen assigned by the randomization code and the participant's weight (Appendix 6). An electronic, password-protected copy of the randomization codes will be kept at TDR, Geneva.

The opened envelopes will be signed, dated, resealed and kept in the locked cabinet.

3.4. *Blinding:*

Blinding of the study will be achieved by ensuring that the person who calculates the dosage of PZQ to be given and provides the medication to the participants is not the person who evaluates safety. In addition those doing the parasitological analysis will not know the treatment given to the individual children.

4. SELECTION AND WITHDRAWAL OF PARTICIPANTS:

The inclusion and exclusion criteria will be as follows:.

4.1. *Inclusion criteria*

- Age 10-19 years
- Infected subjects with an egg excretion of ≥ 100 eggs per gram of faeces for *S mansoni*.
- Those able and willing to be followed up
- Who have given written informed consent for stool, urine and blood samples. In case of adolescents (< 18 years), written informed consent from their parents and their verbal assent to be obtained.

Those who have satisfied the inclusion criteria will be further tested for the following exclusion criteria:

4.2. *Exclusion criteria*

- Known previous history of adverse reactions associated with PZQ
- Pregnancy (by testing) or lactating
- Clinical malaria
- History of treatment in the past 30 days with PZQ
- Currently or in the past week using other medication that may affect the results of the present trial, as antibiotic
- Acute or chronic severe disease, including hepato-splenic schistosomiasis.
- Mixed *S mansoni* and *S haematobium* infections

The study will be carried out in a school setting and only children fulfilling the inclusion/exclusion criteria will be recruited for the study. Depending on the prevalence of infection on a specific school, it is expected that many children (usually at least half of them) won't fulfill all the criteria. The main reason for excluding children will be: because they do not have schistosomiasis or they don't have the required infection intensity of ≥ 100 eggs/g of stools for *S. mansoni*. As for all information obtained for the trial, the data will be kept confidential, and the reason why a child was not included in the study will not be released to the community. Teachers will be asked to assist the study team to deal with the problem of possible differentiation between those participating and not participating in the trial. All those infected but not included in the study will still be treated.
The same will happen with pregnant girls. As stated before, they will be informed about their condition and counseled by the study nurse or physician, and referred to the Pre Natal assistance. However, this information will be kept confidential and only released to the person that it refers to.

4.3. *Subject withdrawal criteria*

The study subject will be withdrawn from the trial if he/she presents any illness or condition that makes further participation impossible, or if he/she decides not to participate. The participant will be discontinued to participate in the study if during the follow-up period he/she takes an anti-schistosomal medication.
Withdrawn participants will not be replaced. Individual children will be free to withdraw from the study for whatever reason they chose.

5. TREATMENT OF SUBJECTS:

Those schoolchildren who fulfilled inclusion criteria will be recruited for the trial. A total of 236 schoolchildren (see sample size calculation on section 10.2) with *S mansoni* who fulfilled the inclusion/exclusion criteria would be randomly assigned to two groups of 118 each. One group will be given a single dose of PZQ 40 mg/kg and the other a single dose of PZQ 60mg/kg body weight. Table 2 below illustrates the treatment groups.

Table 2. Study groups for and *S mansoni* treatment.

Dosage of PZQ/kg body weight	*S.mansoni* subjects (N)
40 (single dose) – Control	118
60 (single dose)	118

Praziquantel is provided in tablets of 600mg with 3 scores, which may be divided in 4 segments of 150 mg each. The amount of drug to be given can be adjusted to the patient's bodyweight by breaking the tablets in 4 segments. A reference table for the number of tablets to be given for each participant according to treatment regimen and body weight is presented in Appendix 2.

5.1. Compliance to treatment:

The medication will be given and directly observed by the person designated to provide treatment. If the participant vomits within 30 minutes after taking the medication, a new dose will be given.

5.2. Medications permitted and not permitted:

All medications except those with anti-schistosomal activity will be allowed during the trial. After the 21 days safety assessment, participants with diagnosis of intestinal helminths will be treated with mebendazole or albendazole. Those with anemia (as defined in section 6.2.4) will receive iron supplementation. If the participant has severe anemia he/she will be referred to appropriate health care.

5.3. *Accountability of the investigational product (PZQ)***:**

The PZQ for this trial will be received by the Principal Investigator and stored at room temperature (temperatures between 18ºC and 24ºC) in the Institute's drug store. The PI will keep a register of drugs received, dispensed and returned from the field.

6. CLINICAL AND LABORATORY PROCEDURES

6.1. *Clinical examination:*

6.1.1. Clinical malaria

Signs and Symptoms of clinical malaria will be determined (by one of the study physicians) on schoolchildren recruited in the study after satisfying inclusion criteria before treatment with PZQ. Those with fever (temperature ≥ 37°C) and/or showing one or more of the following signs and symptoms, i.e., body malaise, headache, vomiting or diarrhoea will be considered to have clinical malaria will referred for treatment at a nearby clinic and excluded from the study.

6.2. *Laboratory examination*

6.2.1. Pregnancy

Eligible female on childbearing age satisfying inclusion/exclusion criteria, after giving informed consent, will be examined for pregnancy using the Quickstick™ One step HCG Pregnancy Test. Each participant will provide a fresh urine specimen and tested within 24 hr of sample collection. The test device (dipstick) is dipped in the urine sample and results are read in 5 minutes. The presence of two colour bands (pink to purple) within the test region of the device indicates a positive result. The test is, however, sensitive 7 days after a female has conceived. Each participant with positive pregnancy test will be counselled by one of the study nurses.

6.2.2. Stool:
At baseline and during follow-up visits each child in schools within 5 km of the Lake Victoria shore recruited in the study will provide a stool specimen on two separate days at maximum interval of 5 days. Duplicate Kato Katz slides (2 slides) (Katz et al., 1972) will be prepared in the field (using the 41.7mg faecal template) from each stool sample. The prepared Kato slides in the field will undergo quantitative microscopic examination for *S mansoni* ova.
Classification as egg count ≥ 100 eggs per gram of faeces for *S mansoni* will be obtained by the mean egg count for the 4 slides collected at baseline.

6.2.3. Urine
At baseline, as outlined in Table 1, each child in schools recruited in the study will provide one urine specimen between 10am and 2pm. The urine sample will be semi-quantitatively examined for *S haematobium* infection by reagent strips (Hemastix® Bayer) reading for microhaematuria.
Participants positive for *S. haematobium* will be excluded from the study, but treated with the standard regimen of PZQ 40mg/Kg.

6.2.4. Blood
Finger prick blood will be obtained in a Hemocue cuvette from each school child at baseline and follow-up visits D21, Month 6 and Month 12 and examined for haemoglobin level (Hb) by a digital hemoglobinometer (Hemocue). Hb reading of ≤11g/L will be considered as anaemia and that of ≤ 7g/L as severe anaemia.

6.2.5. Weight
Weight of schoolchildren will be measured at baseline, at 6-months and 12 –months post treatment with PZQ. A digital, battery operated, electronic balance (Tanita digital Model THD - 305) will be used to weigh schoolchildren.

7. ASSESSMENT OF EFFICACY:

7.1. Parameter to be measured – egg count, as measured by the Kato-Katz method described above, at baseline, D21, 6M and 12M
The measurement of egg count for each time point will be estimated as the mean of the 4 slides collected (2 slides per sample, 2 samples within 5 days).

7.2. Methods for assessing efficacy:

- Cure rate will be estimated as the proportion of subjects with negative result at 21 days after treatment.

- Egg reduction rate at 21 days after treatment will be estimated through the following formula:

[1 - (epg2 / epg1) x 100] where epg1 and epg2 are the geometric mean of $\log_{10}$ transformed (x+1) numbers of eggs per gram of faeces at the screening survey and the 21st day post-treatment survey.

The same calculations of cure rate and egg reduction rate will be estimated for the 6M and 12M follow-up in order to estimate reinfection rates.

7.3. Analysis: The cure rate and egg reduction rate will be compared between the 2 treatment regimens, PZQ 40mg/Kg and 60mg/Kg.

8. ASSESSMENT OF SAFETY:

Adverse Events

8.1. *Parameters to be measured:*
Ocurrence and severity of Adverse Event at 4 hours, 24hs, and 21 days after treatment.

8.2. *Methods:* All AEs will be characterized for: severity grading, relationship to the treatment and seriousness.

Treatment with PZQ will be done in the morning of D0 and the research team will remain at the treatment centre until 18.00 hr. Children administered with PZQ will be observed for at least 4 hours before leaving the school compound.
All schoolchildren enrolled in the study will be administered with a questionnaire 4 hours after treatment for the following signs and symptoms: abdominal pain, nausea, vomiting, diarrhoea, anorexia, fever, headache, dizziness and allergic reaction. Each symptom will be graded as described in Table 1 of Appendix 3. Other symptoms not listed in Table 1 will be graded for severity according to classification in Table 2 of Appendix 3. Also, causal assessment for the adverse events in relation to the drug will be done as: not related, unlikely, possible, probable, most probable or insufficient data to assess (see definitions in Appendix 4). On the following day (Day 1), that is, 24 hours post-treatment, all schoolchildren who received PZQ on Day 0 will be interviewed using the same questionnaire to determine whether they experienced any adverse events. The questionnaire will be repeated on day 21-post treatment. In addition, on day 21 Hb will be measured by digital hemoglobinometer (hemocue) to determine whether increased dosage of PZQ has adverse effects on haemoglobin level.

All AEs will be evaluated for seriousness according to the definitions described in Appendix 5. Any immediate serious adverse events will be managed using a resuscitation kit comprising of intravenous N/saline, Adrenaline, Hydrocortisone and carefully monitored until the adverse event disappears completely. All AEs will be monitored and followed-up until resolution. An adverse event likely to be related to the administration of the investigation product which persists to the end of the trial or any serious adverse event occurring after the termination of the trial and likely to be related to the product, will be followed up by the study physicians until its complete disappearance. The causality of such a serious adverse event will be investigated and recorded. Within 24 hours of its occurrence the Principal Investigator will report such serious adverse event to the TDR Clinical Monitor and TDR Clinical Coordinator by a SAE alert form. A complete SAE report will be sent within 5 working days to the Clinical Monitor and Clinical Coordinator. During the forwarding of the information the anonymity of the subject will be maintained. All this will be done in compliance to applicable regulatory requirements related to the reporting of unexpected serious adverse drug reactions to the regulatory authority(ties) and the ICB/IRB.

8.3. *Analysis:* The frequency and severity of AEs will be compared between the 2 treatment regimens, PZQ 40mg/Kg and 60mg/Kg.

9. ASSESSMENT OF MORBIDITY:

9.1. *Parameters to be measured:*
Hb level and weight at baseline, 6M and 12M.

9.2. *Methods:* Hb level and weight will be measured as described on sections 6.2.4. and 6.2.5.

9.3. *Analysis:*

- Variation of Hb level (as described above) will be compared between the 2 treatment regimens, PZQ 40mg/Kg and 60mg/Kg.
- Variation of weight will be compared between the 2 treatment regimens, PZQ 40mg/Kg and 60mg/Kg.

10. DATA PROCESSING AND ANALYSIS

The data will be double entered into a computer using dBase programme by two different data entry clerks and the entered data will then be verified and cleaned before being subjected to analysis using STATA.

10.1. *Analysis of data:* Chi- square or Fisher's exact test will be used for comparison of proportions (ex. cure rate & egg reduction rate, frequency of AEs and severity of AEs) and Student's t test for comparison of means (geometric mean egg output, variation of Hb level and weight) in the two treatment groups (PZQ 40mg/kg and 60mg/kg).

10.2. *Sample size:*
Assuming the cure rate of a standard dosage (40mg) of Praziquantel is 60% and increasing the dosage to 60mg would improve cure rate to *at least* 80%, a sample size of 91 infected schoolchildren per study group will be needed. This sample size will give a study power of 80% at 5% significance level. Considering a drop out of about 30% due to loss to follow-up or medical grounds, 118 study subjects will be needed for each study group making a total of 236 schoolchildren.

10.3. *Quality Control:*
During laboratory examination of stool and urine specimens, a 10% sample of specimens will be re-examined by an independent technician (one who is not part of the research team) every day and results compared to see the degree of agreement.

10.3.1. For Kato-Katz examinations:
10% of the slides read by each microscopist will be randomly selected and re-examined by an experienced microscopist designated by the PI.

For cure rate (agreement between positive/negative slides) the maximum discordance rate acceptable will be 5%. Above 5% all slides will have to be read by the experienced microscopist.

For infection intensity, levels of infection will be defined as:

Number of eggs/slide	Approximate egg count/g of stools
1-4	~ 1-100
5-12	~ 100-300
13-25	~ 300-600
26-41	~ 600-1.000
>41	~ 1.000

Disagreement will be defined when the 2 readings result in different levels of infection, according to this classification. The maximum level of disagreement accepted will be 5%.

If the conditions for agreement are not met, all slides will be read a second time and the results of the second reading will be considered for the study.
If the conditions for agreement are met, the results of the first reading will be used.

11. ETHICAL CONSIDERATIONS FOR PROJECTS INVOLVING HUMAN SUBJECTS

Research clearance for the proposed study has been obtained from the Medical Research Co-ordinating Committee of the National Institute for Medical Research, which acts as the National Ethics Committee in Tanzania and is attached.

At the beginning of the study meetings will be organized with parents and teachers of the earmarked schools, whereby detailed information will be provided by the research team about the aims, procedures, benefits and potential risks of the study.
Written informed consent will be obtained from all participants. In case of minors, the oral assent of the child will be asked, besides the written consent of the guardian. If the children is between 10-<14, and according to the Investigator's perception, he/she is not able to understand the information given during the informed consent procedure, the oral assent is not necessary.

Participation in the study will be on voluntary terms and that an individual will be free to withdraw from the study at any time. Withdrawal from the study will not affect ones statutory rights in any way.

All records of study subjects will be kept confidential.

All schoolchildren participating in the study and found to have schistosomiasis will be treated free of charge. Participants with schistosomiasis who do not fulfil the criteria for enrolment will be treated with the standard regimen of single dose Praziquantel at 40 mg/Kg.

Only parasitological specimens (Kato-Katz slides) will be kept for the duration of the study to allow for quality control and cross-checking. They will then be discarded. No other specimens will be kept.

12. CRITICAL ASSESSMENT AND POSSIBLE LIMITATION OF APPROACH IN RELATION TO PROJECT OBJECTIVES

12.1. Missing of a single schistosome egg during microscopy in light infections may cause bias in determination of cure rate and egg reduction rate (efficacy) of the investigational product. To minimise bias only subjects with high egg count are included in the trial (see inclusion criteria)

12.2. Praziquantel does not affect immature stages of schistosomes. In order to avoid eggs from immature stages that are not affected by PZQ at the time of treatment, the end point in determination of efficacy is optimised at day 21.

APPENDIX 1

Baseline Screening, Treatment and Safety Assessment Schedule:

Calendar	Day	No. of subjects Screened	No. Kato Slides Made	Urine samples screened	Stool microscopy	Computer Data entry	D0	D1	D21
1	Mon	100	200	100					
2	Tue		200	100	200				
3	Wed	100	200	100	200	Xx			
4	Thur		200	100	200	Xx			
5	Fri	100	200	100	200	Xx			
6	Sat								
7	Sun								
8	Mon		200	100	200	xx	X1		
9	Tue	100	200	100	200	xx		X1	
10	Wed		200	100	200	xx	X3		
11	Thur	100	200	100	200	xx		X3	
12	Fri		200	100	200	xx	X5		
13	Sat							X5	
14	Sun								
15	Mon	100	200	100	200	xx	X9		
16	Tue		200	100	200	xx		X9	
17	Wed	100	200	100	200	xx	X11		
18	Thur		200	100	200	xx		X11	
19	Fri	100	200	100	200	xx			
20	Sat								
21	Sun								
22	Mon		200	100	200	xx	X15		
23	Tue				200	xx		X15	
24	Wed					xx	X17		
25	Thur							X17	
26	Fri								
27	Sat								
28	Sun								
29	Mon						X19		
30	Tue							X19	
31	Wed	30	X1			xx			X1
32	Thur		X1		X1	xx			
33	Fri	30	X3		X1	xx			X3
34	Sat		X3		X3	xx			
35	Sun								
36	Mon	30	X5		X3	xx			X5
37	Tue		X5		X5	xx			
38	Wed	30	X9		X5	xx			X9
39	Thur		X9		X9	xx			
40	Fri	30	X11		X9	xx			X11
41	Sat		X11		X11	xx			
42	Sun								

43	Mon	30	X15		X11	xx			X15
44	Tue		X15		X15	xx			
45	Wed	30	X17		X15	xx			X17
46	Thur		X17		X17	xx			
47	Fri				X17				
48	Sat								
49	Sun					xx			
50	Mon	30	X19			xx			X19
51	Tue		X19		X19	xx			
52	Wed				X19	xx			
53	Thur					xx			

Key: X1, X3 etc. refers to the day in which schoolchildren were first screened for schistosomiasis e.g., X1 screened on day 1 of the study calendar.

Appendix 2.

Table 1. Number of praziquantel tablets to be given to subjects under the 40mg/kg and 60mg/kg regimens.

40 mg/kg regimen		60 mg/kg regimen	
**Bodyweight (kg)**	**Number of tablets**	**Bodyweight (kg)**	**Number of tablets**
13-15	1	10	1
16-18	1 ¼	10 - 12.5	1 ¼
19 – 22	1 ½	12.6 - 15	1 ½
23 – 25	1 ¾	15 - 17.5	1 ¾
26 – 29	2	17.6 - 20	2
30 – 33	2 ¼	20 - 22.5	2 ¼
34 – 37	2 ½	22.6 - 25	2 ½
38 – 40	2 ¾	25 - 27.5	2 ¾
41 – 44	3	27.6 - 30	3
45 – 48	3 ¼	30 - 32.5	3 ¼
49 – 52	3 ½	32.6 - 35	3 ½
53 – 55	3 ¾	35 - 37.5	3 ¾
56 – 59	4	37.6 - 40	4
60 – 63	4 ¼	40 - 42.5	4 ¼
64 – 66	4 ½	42.6 - 45	4 ½
67 – 70	4 ¾	45 - 47.5	4 ¾
71 – 75	5	47.6 - 50	5
		50 - 52.5	5 ¼
		52.6 - 55	5 ½
		55 - 57.5	5 ¾
		57.6 - 60	6

In this table, 13-15 means 13.1 kg to 15.9 kg. The same applies to the other weight ranges

APPENDIX 3.

Table 1. Grading scale for determining the severity of adverse events:

Clinical sign/symptom	Grade 1	grade 2	grade 3	grade 4
Abdominal pain (2)	Mild	Moderate- no treatment needed	Moderate- treatment needed	Severe- hospitalization for treatment
Nausea (1)	Mild discomfort; maintains reasonable intake	Moderate discomfort; intake decreased significantly; some activity limited	Severe discomfort; no significant intake; activities limited	Minimal fluid intake
Vomiting (2)	1 episode/day	2-3 episodes/day	4-6 episodes/day	Greater than 6 episodes/day or intractable vomiting
Diarrhoea (2)	Slight change in consistency and/or frequency of stools	Liquid stools	Liquid stools greater than 4x the amount or number normal for this child	Liquid stools greater than 8x the amount or number normal for this child
Anorexia (2)	------------	Decreased appetite	Appetite very decreased, no solid food taken	No solid or liquid taken
Fever, oral (1)	37.7 – 38.5 C or 100.0 – 101.5 F	38.6 – 39.5 C or 101.6 – 102.9 F	39.6 – 40.5 C or 103 – 105 F	> 40.5 C or > 105 F
Headache (1)	Mild, no therapy required	Transient, moderate; therapy required	Severe; responds to initial narcotic therapy	Intractable; required repeated narcotic therapy
Dizziness (3)	Not interfering with function	Interfering with function, but not interfering with activities of daily living	Interfering with activities of daily living	Bedridden or disabling
Allergic reaction (1)	Pruritus without rash	Localized urticaria	Generalized urticaria; angioedema	Anaphylaxis

Adapted from (1) WHO Toxicity Grading Scale, (2) Division of Microbiology and Infectious Diseases (DMID/NIH) Pediatric Toxicity Tables, and (3) National Cancer Institute (NCI) Toxicity Grading Scale.

Table 2. Severity grading for other Adverse Events will be estimated as follows:

**Grade 1**	**Mild.** Transient or mild discomfort (< 48hs); no medical intervention/therapy required
**Grade 2**	**Moderate.** Mild to moderate limitation in activity – some assistance may be needed; no or minimal medical intervention/therapy required
**Grade 3**	**Severe.** Marked limitation in activity, some assistance usually required; medical intervention/therapy required, hospitalization possible
**Grade 4**	**Life-threatening.** Extreme limitation in activity, significant assistance required; significant medical intervention/therapy required, hospitalization or hospice care probable.

APPENDIX 4.

Classification according to Relationship to the treatment:

Not related: The event is clearly related to other factors such as the patient's clinical state, therapeutic intervention or concomitant therapy.

Unlikely: The event was most probably produced by other factors such as the patient's clinical state, therapeutic intervention or concomitant therapy and does not follow a known response pattern to the trial product

Possible: The event:

- follows a reasonable temporal sequence from the time of product administration; *and/or*
- follows a known response pattern to the trial product; *but*
- could have been produced by other factors such as the patient's clinical state, therapeutic intervention or concomitant therapy.

Probable: The event:

- follows a reasonable temporal sequence from the time of product administration, *and/or*
- follows a known response pattern to the trial product, *and*
- could have been produced by other factors such as the patient's clinical state, therapeutic intervention or concomitant therapy.

Most probable: The event:

- follows a reasonable temporal sequence from the time of product administration *and/or*
- follows a known response pattern to the trial product, *and*
- could have been produced by other factors such as the patient's clinical state, therapeutic intervention or concomitant therapy, *and*
- either occurs immediately following trial product administration, or improves on stopping the product or there is positive reaction at the application site.

Insufficient data to assess: There is not enough clinical and/or laboratory information to suggest the relationship of the event to the trial product.

APPENDIX 5.

Evaluation of seriousness:

Definition of Serious Adverse Event (SAE)

A serious adverse event is any untoward medical occurrence that, at any dose, has one or more of the following:

- ✓ *results in death,*
- ✓ *is life-threatening*,*
- ✓ *requires inpatient hospitalization or prolongation of existing hospitalization,*
- ✓ *results in persistent or significant disability/incapacity, or*
- ✓ *is a congenital anomaly/birth defect.*

**Note:* the term "life-threatening" in the definition of "serious" refers to an event in which the patient was at risk of death at the time of the event; it does not refer to an event which hypothetically might have caused death if it were more severe

Important medical events that may not be immediately life threatening or does not directly result in death or hospitalization but may jeopardise the patient or may require intervention to prevent on of the other outcomes listed above should also usually be considered as serious.

Reference

Katz, N., Chaves, A. & Pellegrino, J. (1972). A simple device for quantitative stool thick smear technique in schistosomiasis mansoni. *Revista do Instituto de Medicina Tropical de Sao Paulo*, 14; 397-400

Kumar v, Gryseels B. (1994). Use of Praziquantel against schistosomiasis: a review of current status. *Int J antimicrob. Agents* 4; 313-20.

Lwambo NJS (1988) Transmission of urinary schistosomiasis in Sukumaland, Tanzania. 1. Snail infection rates and incidence of infection in schoolchildren. *Journal of Helminthology*, 62; 213-217

Lwambo NJS, Siza JE, Brookers S, Bundy DAP and Guyatt H (1999). Pattern of concurrent hookworm and schistosomiasis in school children in Tanzania, *Trans, R . Soc. Trop. Med. Hyg.*, 93; 497-502.

Magendants M. (1972) The biology of *Biomphalaria choanomphala* and *B sudanica* in relation to their role in the transmission of *Schistosoma mansoni* in Lake Victoria at Mwanza, Tanzania. *Bulletin World Health Organization*, 47; 331-342

McMahon, JE & Kolstrup N (1979) Praziquantel: a new schistosomicide against *Schistosoma haematobium*. *British Medical Journal, 2;* 1396-1399

Rugemalila JB, Asila J, and Chimbe A. (1984) Randomized comparative trial of single doses of the newer antischistosomal drugs at Mwanza, Tanzania. 1. Praziquantel and Oxamniquine for the treatment of schistosomiasis mansoni. *Journal of Tropical Medicine & Hygiene, 87;* 231-235

Stelma FF, Talla I, Sow S, et al.(1995). Efficacy and side effects of Praziquantel in an epidemic focus in an epidemic focus of *Schistosoma mansoni*. *Am.J Trop Med Hyg,* 53;167-70.

Van Lieshout L, de Tonge N, el-Masry N, Mansour MM, Basilly S. eta al(1994). Monitoring the efficacy of different doses of Praziquantel by quantification of circulating antigens and urine of schistosomiasis patients. *Parasitology*, 108; 519-26

WHO. Prevention and control of schistosomiasis and soil-transmitted helminthiasis: Report of a WHO Expert Committee. WHO technical Report series' No 912. WHO: Geneva, 2002.
