# Supplementary material for: A Multicentre Randomized Controlled Trial of the Efficacy and Safety of Single-Dose Praziquantel at 40 mg/kg vs. 60 mg/kg for Treating Intestinal Schistosomiasis in the Philippines, Mauritania, Tanzania and Brazil
Source: PLoS Negl Trop Dis. 2011 Jun 14;5(6):e1165. doi: 10.1371/journal.pntd.0001165 (PMC3114749; doi:10.1371/journal.pntd.0001165)
Supplement: Protocol S2 — Protocol of the Philippines (PDF) [file pntd.0001165.s004.pdf]

Study Title: "Efficacy and safety of 40 mg/kg and 60 mg/kg single doses of praziquantel in the treatment of schistosomiasis japonicum"

4.1 Objectives and Rationale

1. To evaluate the efficacy and safety of praziquantel 60 mg/kg single dose in the treatment of schistosomiasis, as compared to the standard 40 mg/kg single dose regimen.
a. To compare the cure rates and egg reduction rates of PZQ 60 mg/kg single dose regimen versus the cure rates and egg reduction rates of PZQ 40 mg/kg single dose regimen
b. To compare the prevalence and intensity of PZQ-related side effects among participants who were given the 60 mg/kg regimen and participants given the 40 mg/kg regimen.
c. To describe the PZQ related side effects
2. To compare the reinfection rate at 6 months and 12 months after treatment between the two treatment regimens

Schistosomiasis is endemic in 24 provinces in the Philippines. Agusan del Sur is included in these, in which 13 out of the 14 municipalities of the province are endemic for schistosomiasis. Currently, chemotherapy with praziquantel (PZQ) remains as a major strategy for morbidity reduction from schistosomiasis. A single dose of PZQ 40 mg/kg is generally sufficient to give cure rates of 60 to 90% and egg reduction rates of 90 to 95% (WHO, 1998). This method of administration is preferable because of higher compliance rate. However, in areas of high schistosomiasis transmission, cure rates for the single dose have been lower than expected (Greyseels et al.). Therefore, for treatment of mixed infections of Schistosoma japonicum and S. mekongi, a dose of 60 mg/kg in two divided doses has been recommended (WHO, 1998). Lower compliance rates for this treatment regimen have been described. This is the reason why there is a need to test the efficacy and safety of praziquantel 60 mg/kg as a single dose regimen. Locally, the cure rate for praziquantel 60 mg/kg in two divided doses has been reported to be 80%, while the egg reduction rate was 96% (Santos et al, 1984). Results of this study will be important in the optimization of praziquantel use for more effective schistosomiasis control.

4.2 Experimental design and methods

Name and description of investigational products

Praziquantel is an isoquinoline-pyrazine derivative (2-cyclohexylcarbonyl-1,3,4,6,7,11b-hexahydro-2H-pyrazino (2,1-a)isoquinoline-4-one). It has a broad antiparasitic effect and first marketed as a veterinary taeniacide under the trade name Droncit (Bayer) MacMahon &Kolstrup (1979). Clinical studies in man have shown that it is effective in human tapeworm infection and all human schistosome infections. Praziquantel is supplied in 600 mg tablets for oral administration.

Summary of the potential risks and benefits, if any to human subjects

Praziquantel has been known to cause the following adverse effects commonly in patients with heavy worm loads: (1) abdominal discomfort, (2) nausea, (3) vomiting, (4) diarrhea, (5) anorexia, (6) fever, (7) headache, (8) dizziness and (9) allergic reaction (WHO model prescribing information: Drugs used in parasitic diseases, 1995).

No serious AEs have been reported related to the Public Health use of praziquantel over the past 30 years, that is now considered safe enough for use during pregnancy and lactation (Allen *et al*., 2002; WHO, 2002; Olds G.R., 2003).

Description and justification for the route of administration, dosage regimen and treatment periods

Praziquantel will be administered in a single dose at the standard 40 mg/kg or at 60 mg/kg. The WHO originally recommended the 60 mg/kg regimen to be given in 2 divided doses for safety reasons. However, compliance rate for this treatment regimen is poor, which can compromise public health interventions. This is the reason why there is a need to test the efficacy and safety of praziquantel 60 mg/kg as a single dose regimen.

Study Site

The study sites will be the municipalities of Bunawan and Trento (Table 1), which are located in the province of Agusan del Sur in Mindanao Island, Philippines. Agusan del Sur covers most of the Agusan river valley. Mountain ranges border its eastern and western sides. It had a total population of 559,294 in the year 2000. Fifty percent of the population is below 18 years, and 54.3% of the population is 15 to 64 years of age, which is the economically active age group. Individuals aged less than 15 years old composed 43% of the population. The working population is only slightly larger than the dependent population. In the year 2000, for every 100 working individuals there were 84 dependents. More than half or 55% of the population completed elementary education while only 5.5% were able to reach college.

Thirteen out of 14 municipalities of Agusan del Sur are endemic for schistosomiasis. Prevalence may be considered moderate, making the municipalities suited for a targeted mass treatment strategy. The peak of transmission for schistosomiasis in this area is during the rainy season from the months of June to September.

Bunawan (Table 2) had a total population of 26,533 in the year 2002, while Trento (Table 3) had a total population of 13,054 in the same year. Bunawan and Trento are predominantly agricultural municipalities, producing mostly rice. Rainfall occurs constantly throughout the year. In addition to the many clear fresh water streams and water seepage areas along mountains, the rich vegetation provided by the fertile valley soil, provide conducive environs for the proliferation of *Oncomelania hupensis quadrasi*, which is the intermediate host of *Schistosoma japonicum*. The two municipalities are also endemic for malaria.

The study will be based in elementary level schools (intermediate) from each of the chosen barangays (villages) as well as high schools where residents of Bunawan and Trento attend school. This school-based study will target residents of the said barangays ages 10 to 19 years old.

Study Group

The 10 to 19 years age group is the target population for this study because this group is at high risk for *S.japonicum* transmission. This age group partly includes the 5 to 14 years age group (Table 4), which has the highest prevalence and intensities of infection. The latter also presents high prevalence of pathology, and high intensities of infection, thus playing a major role in transmission.

Study population

Study participants will come from barangays in Bunawan and Trento. The total number of required subjects is 218 (see statistics section). One hundred nine participants will be randomly assigned to receive praziquantel 60 mg/kg single dose and another 109 will be assigned to receive praziquantel 40 mg/kg single dose. The sample size has been increased by 20% to give allowance for withdrawals.

Recruitment of participants will be done in the schools during school days. The project team will meet with the parents of the targeted study population during the Parent Teachers Association (PTA) meeting. During this meeting, the contents of the patient information sheet will be explained and group informed consent will be obtained. The strength of this system is that the participants i.e. pupils are a captive group, in the sense that they will be easy to follow-up and monitor. Stool samples from non-school based participants will still be examined but they will not be eligible for inclusion in the study.

The trial is a randomized, double blind, experimental study. Study participants will be randomly assigned to one of the two drug regimens being tested. The recommended randomization procedures will be followed in the study, in which participants will be divided in blocks of 4, in a ratio of one is to one for each drug regimen, praziquantel 40 mg/kg or praziquantel 60 mg/kg. Sealed and numbered envelopes will be provided by TDR. The envelopes containing the codes will be kept in a locked cabinet and one designated person in the site will be responsible for them. The latter will also be responsible for opening the envelope and verifying the corresponding treatment once a new patient is enrolled in the study. Each patient will be assigned a unique Study I.D. number. This I.D. number will be assigned in sequential order and will be recorded in the screening log. The opened envelopes will be signed, dated, resealed and kept in the locked cabinet. The person who will calculate the dosage and administer the drugs will not be tasked to evaluate safety. The person(s) responsible for clinical evaluation and safety assessment will also be blinded to the treatment assignment and to the treatment allocation to the participants. The person who will assess the cure rates and egg reduction rates will have no knowledge of the treatment regimen assigned to the subjects.

<u>The sequence and duration of all trial periods</u>

The study is expected to last for 14 months. It will start at the screening period when the stool samples will be collected and examined. Women in childbearing age will be asked to provide a urine sample to screen for possible pregnancy using a urine pregnancy test kit. Those tested positive will be excluded from the study. Demographic data such as age, sex and birth date will be collected. Trained research assistants will measure anthropometric data, specifically height and weight. Data about the medications received by the subjects will also be recorded. All individuals in the 10 to 19 age group will be eligible for screening. Assuming 22% prevalence of schistosomiasis, the minimum number of participants that should be screened is 991 to obtain the required sample size of 218. Randomized assignment of the treatment regimens will be done after all qualified candidates have been enrolled. The whole population of enrolled participants will be used as basis for randomization. Therefore all the individuals will have an equal chance of being assigned to the 60 mg/kg regimen and the 40 mg/kg regimen regardless of their barangay. The treatment is scheduled at D0, which is at most 1 week from the last screening day. Follow-up visits are scheduled 24 hr (D1), 21 days (D21), 6 months (D180), and 12 months (D360) post treatment. Safety assessment will be done on D0, D1 and D21. Assessment of efficacy, as measured by egg reduction rate and cure rate will also be done on D21. Stool examinations will be done on D180 and D360 to determine reinfection rates, likewise anthropometric measurements will be made. Description of assessments and activities to be undertaken for each visit is shown in Table 5, and a schematic diagram of the study procedures is shown on page 7h.
The participants that for any reason were excluded from the study and who had positive diagnosis of *S. japonicum* will receive standard treatment. Participants who had positive diagnosis of *S. japonicum* or soil transmitted helminth (STH) infection during follow-up visits 21 days (D21), 6 months (D180), and 12 months (D360) post treatment will also receive standard treatment.

<u>Description of the “stopping rules” or “discontinuation criteria”</u>

Individual withdrawal:

The patient will be withdrawn from the study if s/he: (1) presents any illness or condition that makes further participation impossible, (2) decides not to continue his/her participation and , **(3) has completely swallowed the drug and has vomiting within 30 minutes after praziquantel administration**

Participants who will be withdrawn will be properly accounted for in the analysis. Participants who exhibit serious adverse events (SAEs) will not be excluded in the analysis and will be followed up for the whole trial period. Although SAEs are not anticipated because PZQ is considered safe and approved at 60 mg/kg dose, special care will be provided in case this happens.

Lost to Follow-up

The patient is considered as lost to follow-up if s/he has not been able to submit a stool sample or has not been assessed for adverse events within the prescribed follow-up period.

Accountability procedures for the investigational products

Storage for the product will be the responsibility of the research staff. The investigators will inspect the place of storage to determine if it is adequate for the purpose. The rooms will be protected from temperature extremes by air conditioning and proper ventilation. The investigational products will be kept inside a cooler, which will be tightly sealed to protect the products from moisture, contamination and extremes in temperature.

A member of the research staff who will not be tasked to administer the drugs or to evaluate safety will be responsible to account for all used and unused study supplies. The research staff will register the amount of drugs received, dispensed, and returned. He/she will check these records immediately after the delivery of distribution of drugs and will also conduct weekly inventory of the drugs

Maintenance of trial treatment randomization codes and procedures for breaking codes

Recommended procedures in breaking codes will be followed. The randomization codes will only be broken mainly for medical reasons (e.g. in the event of SAEs). Individual randomization codes could be broken and revealed to the investigators or others managing the subject or patient when identification of the drug dosage is required in order to manage the subject/patient i.e. when a medical emergency or serious medical condition has arisen or when the physician in charge of the subject/patient feels that the patient cannot be adequately treated unless the dosage of the drug given is known or if the information is essential for further management of the other subject/patients already included or to be included in the study. The investigator must make every effort to contact the TDR clinical coordinator prior to breaking the code. If this is not possible, and the situation is an emergency, the investigator may break the code and contact the TDR clinical monitor as soon as possible thereafter. Premature unblinding will be reported immediately to the Clinical Monitor and will be documented in the investigators' file. The investigator will return all unbroken codes to the Clinical Monitor to prove blinding throughout.

The identification of any data to be recorded directly on the CRFs

The case record form (CRFs) of the patients will include demographic, anthropometric, and parasitologic data. It will also include the findings of the baseline assessment, efficacy assessment and safety assessment.

SELECTION AND WITHDRAWAL OF SUBJECTS

Inclusion criteria

Patients who will be included as subjects of the study are those who are: 1) aged 10 to 19 years, 2) confirmed cases of *S. japonicum* infection with $\geq$ 100 epg using Kato Katz technique, 3) those with written informed consent, and 4) able and willing to be examined on follow-up visits and provide stool samples.

Exclusion criteria

Those who will be excluded from the study are those who: 1) are pregnant or lactating, 2) have previous history of adverse reaction associated with praziquantel, 3) have acute or chronic severe disease including hepato-splenic schistosomiasis, 4) have used praziquantel within the

last 30 days, 5) smptomatic malaria. (Those with symptomatic malaria infection will be referred to a health center and will be given local standard malaria treatment.)

Withdrawal criteria

Participants will be withdrawn from the study as soon as they are found to meet any of the withdrawal criteria described earlier. In such case they will be requested to provide one more stool sample for examination. The results of the examination will be recorded for documentation and properly accounted for in the analysis. The reason(s) for withdrawal from the study will also be determined and recorded. Should they decide to leave the area of study, they will be issued health certificates to be presented to the health authorities in the area where they will migrate. This health certificate will contain the possible adverse effects of the treatment given to them. It will also contain instructions to the health authorities on how these adverse effects should be managed. They will also be advised to have medical check-ups on the scheduled safety assessment visits.

TREATMENT OF SUBJECTS

Praziquantel will be provided in tablets of 600 mg with 3 scores, which may be divided in 4 segments of 150 mg each. The amount of drug to be given can be adjusted to the patient's body weight by breaking the tablets in segments. The person who will calculate the dosage and administer the drugs will not be tasked to evaluate safety. The drugs will be individually given to the study participants inside the treatment room to minimize comparison of the number of tablets taken. It will be administered using the two treatment regimens, 40 mg/kg single dose and 60 mg/kg single dose. Tables containing the number of tablets to be given according to treatment regimen and patient's bodyweight will be used as reference (Table 8).

<u>Medication(s)/ treatment(s)/ permitted (including rescue medication)</u>

As stated in the exclusion criteria, individuals who used praziquantel in the previous 30 days, and individuals currently in antibiotics will be excluded from the study. Mebendazole 500 mg chewable tablets will be administered to participants found positive for helminthes infection after D21 of the study to avoid interference for safety assessment of praziquantel.

Procedures for monitoring subject compliance

Treatment will be administered under observation (Directly observed treatment).

ASSESSMENT OF EFFICACY

The efficacy parameters that will be used are cure rate and egg reduction rate measured at 21 days after treatment.

Methods and timing for assessing, recording, and analyzing efficacy parameters

The Kato Katz technique will be used for baseline quantitative assessment of the *S. japonicum* infection, egg reduction rate and cure rate as recommended by the WHO. The WHO Bench Aids for the Diagnosis of Intestinal Parasites will be used as reference for the proper conduct of this technique. In the baseline assessment, 2 stool samples will be collected in 2 different days, with no more than 5 days interval. For each sample, 2 slides (41.7 mg) will be done. Patients with an average of ≧ 100 eggs per gram of feces are considered confirmed cases of *S. japonicum* infection. Egg count reduction will be estimated by: [1 - (epg2 / epg1) x 100] where epg1 and epg2 are the geometric mean of log_{10} transformed (x+1) numbers of eggs per gram of faeces at the screening survey and the 21st day post-treatment survey. During D21, D180 and D360, a stool sample will be collected and two slides (41.7 mg) will be done. The average reading of the 2 replicates in eggs per gram of feces will be considered as the final reading.

ASSESSMENT OF SAFETY

Safety assessment will be gathered through questionnaire on D0, D1 and D21. The questionnaire will begin with an open question regarding any symptoms after the treatment with praziquantel. Patients will be observed on D0 for at least 4 hours after treatment based on the curve of praziquantel absorption. The half-life of the drug is about 0.8-1.5 hours and the plasma maximum concentration is reached at 1 to 3 hours after a single oral dose of praziquantel.

Safety parameters

Parameters for safety assessment will be the prevalence and intensity of the symptoms discussed earlier: (1) abdominal discomfort, (2) nausea, (3) vomiting, (4) diarrhea, (5) anorexia, (6) fever (using an oral thermometer), (7) headache, (8) dizziness and (9) allergic reaction. The severity of the symptoms will be categorized as “mild”, “moderate”, “severe” and “life-threatening” (Tables 6a and 6b). Symptom’s relationship to treatment will be categorized as “not related”, “unlikely”, “possible”, “probable” and “most probable” (Table 7).

Adverse drug reaction (ADR)

All noxious and unintended responses to a medicinal product related to any dose should be considered adverse drug reactions (the phrase ‘responses to a medicinal product’ means that a causal relationship between a medicinal product and an adverse event is at least a reasonable possibility, i.e. the relationship cannot be ruled out).

Regarding marketed medicinal products: a response to a drug which is noxious and unintended and which occurs at doses normally used in human subjects for prophylaxis, diagnosis, or therapy of diseases or for modification of physiological function (Karbwang and Pattou, 2001).

Adverse event (AE)

Any untoward medical occurrence in a patient or clinical investigation subject administered a pharmaceutical product which does not necessarily have a causal relationship with this treatment. An adverse event (AE) can therefore be any unfavourable and unintended sign (including an abnormal laboratory finding), symptom, or disease temporally associated with the use of a medicinal (investigational) product, whether or not related to the medicinal (investigational) product (Karbwang and Pattou, 2001).

Serious adverse event (SAE) or serious adverse drug reaction (serious ADR)

Any untoward medical occurrence that, at any dose, has one or more of the following attributes:

- Results in death.
- Is life-threatening.
- Requires in-patient hospitalization or prolongation of existing hospitalization.
- Results in persistent or significant disability/incapacity.
- Is a congenital anomaly/birth defect.
- Results in an important medical event that may not be immediately life-threatening or does not directly result in death or hospitalization, but which may jeopardize the patient or may require intervention to prevent the other outcomes listed above (Karbwang and Pattou, 2001).

The term “life-threatening” in the definition of “serious” refers to an event in which the patient was at risk of death at the time of the event; it does not refer to an event which hypothetically might have caused death if it were more severe.

Methods and timing for assessing, recording, and analyzing safety parameters

For safety assessment, the participants will be observed for 4 hours after taking the medication and all AEs will be recorded. At the end of the 4 hours observation, the participants will be interviewed. Then subjects will be interviewed 24 hr after receiving the treatment.

Additional safety assessment visit will be scheduled on D21, in conjunction with the efficacy assessment. History and physical examination by a trained project physician will be done during the visits. Recording of the adverse events shall include: (1) date and time of onset, (2) duration, (3) severity, (4) seriousness and (5) relationship to treatment. The report should include a probable explanation from the investigator as to the cause of the adverse event.

Procedures for eliciting, recording, and reporting of adverse event and intercurrent illnesses

All adverse events occurring during the safety assessment period will be reported. This will be included in the participant's CRF. These data will be analyzed at the end of the safety assessment period.

Adverse experiences already documented from previous visits will be reviewed and its duration noted to determine if it is a continuing symptom. Changes in frequency and severity of the symptoms observed in earlier safety visits should be properly noted in the CRF.

All SAEs will be reported immediately (within 24 hours) to the TDR Clinical Monitor and the TDR Clinical Coordinator by the investigator, even if the adverse event is not considered related to the treatment. Notification should be made by faxing the alert form for SAE and/ or by email or telephone communication. The investigator will send within 5 working days the SAE report form by fax, email or express mail, to the TDR Clinical Monitor and the TDR Clinical Coordinator.

The type and duration of the follow-up of subjects after adverse events

Adverse events that occur within the safety assessment period will be properly assessed, recorded, and managed accordingly. The participant concerned will be treated immediately even before investigation as to the real cause of the adverse events or adverse drug reactions are completed. The identification of the real cause of the adverse drug reaction or adverse event will only serve for proper documentation and correct assessment of the safety of the drug.

STATISTICAL ANALYSIS

Comparison of baseline characteristics between the participants given the two treatment regimens will be done to evaluate if randomization was properly done using t-test or Chi square test where appropriate.

Cure rates for the two regimens will be compared using Chi square tests. The geometric mean of $\log_{10}$ transformed (x +1) numbers of eggs per gram of feces at the screening survey and on the 21st day post treatment survey will be computed. Repeated measures analysis of variance (ANOVA) will be performed to determine if there is a difference in the mean log egg counts of praziquantel 40 mg/kg and 60 mg/kg single doses. Chi square test will be used to compare the reinfection rates at D180 and D360 for the two treatment regimens. Chi square test and Fisher's exact test will be used to determine if the prevalence of adverse reactions is significantly different for subjects administered with the 60 mg/kg regimen as compared to subjects administered with the 40 mg/kg regimen. Analysis of all data will be done at the end of the study when all the data have been collected.

The number of study participants planned to be enrolled

Considering the expected cure rate for PZQ 60 mg/kg regimen is 80% and 60% for PZQ 40 mg/kg and assuming 80% power and 95% confidence level, the required sample size computed is 91 individuals per treatment regimen. Considering 20% lost-to-follow-up, the sample size has been adjusted to 109 individuals per treatment regimen or a total of 218 participants. Randomization will be done based on the whole population of enrolled participants. Therefore, there would be participants receiving either PZQ 60 mg/kg or PZQ 40mg/kg in all the barangays.

Level of significance to be used

All the statistical analyses will be performed at 80% power and 5% level of significance.

Procedure for accounting for missing, unused, and spurious data

Patients with missing, unused, and spurious data results will not be accounted for in the analysis but will be recorded for documentation requirements.

Selection of subjects to be included in the analyses

All participants with complete demographic, anthropometric, efficacy assessment and safety assessment data will be included in the analysis.

Direct access to source data/documents

The sponsor is assured that the investigator(s) will permit trial-related monitoring, audits, IRB/IEC review, and regulatory inspection(s) providing direct access to source data documents.

Quality control and quality assurance

The investigators will follow the accepted procedure of having 10% of all KK slides reread by a reference microscopist. Highly unusual findings such as very high egg counts and presence of uncommon parasites will be reviewed by the reference microscopist. Data will be double entered by different personnel and will be further validated.

4.3 ETHICAL CONSIDERATIONS

The method of eliciting and documenting the informed consent forms will be free and without any form of coercion or undue influence from the investigators. The contents of the patient information sheet will be explained and informed consent will be obtained from the parents or legal guardians of the study participants during the Parent Teachers Association (PTA) meeting or prior to enrollment in the study. The project physician or the research staff will explain the terms of the consent form to the participants. Consent could be obtained from the study participant if he/she is ≥ 18 years old. Either, the parents or the legal guardian plus the study participant will be given the right to give consent if the study participants' age is < 18 years old. The recruitment of illiterate subjects should take place in the presence of a literate witness. The witness should be selected by the subject and he/she should have no connection to the research team. The witness should also sign the certificate of consent, confirming that the subject has been properly informed and voluntarily consents to participate in the study. Potential benefits to the patients will be explained together with the risks. Health education will also be provided to the patients. As incentive, participants will be given snacks and transportation allowance as necessary. The participants will be guaranteed with medical care if ever AEs happen during the course of the trial. In case of SAEs, there will be no waiver of participants' rights. The consent forms will contain the information prescribed by the guidelines on Good Clinical Practice.

The safety of the subjects will be assured through proper surveillance and procedures for handling adverse events. Likewise, screened subjects with symptomatic malaria will be referred to a health center to be given standard local malaria treatment. Appropriate treatment will also be provided to subjects who will experience adverse events. The investigators shall guarantee transparency by keeping the subjects informed about all the aspects of the study. They will also assure confidentiality of information on the subjects. The Ethics Review Board of the University of the Philippines Manila College of Medicine shall review all the procedures and measures used in this study. The stipulations of the Declaration of Helsinki shall also be followed in the conduct of this study.

Women with positive test for pregnancy will be counseled by the study nurse or physician and will be referred to the Pre Natal assistance. They will be excluded from the study.

Participants that for any reason were excluded from the trial and who had positive diagnosis of *S. japonicum* will receive standard treatment.

4.4 CRITICAL ASSESSMENT AND POSSIBLE LIMITATION OF APPROACH IN RELATION TO PROJECT ACTIVITIES

Loss to follow up of subjects is one of the foreseen limitations of the study. Biological differences between subjects may also affect the manifestation of the adverse effect that will be observed. It is possible that the sample distribution may lean more on the side of subjects more prone to develop side effects or more sensitive to praziquantel.

Annexes

Table 1. Schistosomiasis Positivity Rates By Kato Stool Examination
Bunawan and Trento, Agusan del Sur

Endemic Municipality	CY 1997	CY 1998	CY 2002	REMARKS (For CY 2002 Case Finding)
Bunawan	21	20	7.07	47.60% Endemic Population Covered
Trento	12	11	11.26	76.65% Endemic Population Covered

Table 2. Schistosomiasis Positivity Rates By Kato Stool Examination According To Barangay (village)
Bunawan, Agusan del Sur

Barangays	CY 1997	CY 1998	CY 2002	REMARKS (For CY 2002 Case Finding)
BUNAWAN	21	20	7.07	47.60% Endemic Population Covered
1. Pob. Bunawan	--	--	3.55	72.39% Endemic Population Covered
2. San Teodoro	--	--	4.78	53.75% Endemic Population Covered
3. Consuelo	50	--	4.20	89.24% Endemic Population Covered
4. San Andres	--	--	4.34	36.10% Endemic Population Covered
5. Libertad	19	20	14.84	34.62% Endemic Population Covered
6.Bunawan Brooks	--	--	0.54	20.67% Endemic Population Covered
7. Nueva Era	--	--	8.57	75.27% Endemic Population Covered
8. Mambalili	--	--	8.46	53.98% Endemic Population Covered
9. San Marcos	--	--	4.05	19.89% Endemic Population Covered

Table 3. Schistosomiasis Positivity Rates By Kato Stool Examination According To Barangay (village)
Trento, Agusan del Sur

Endemic Barangays	CY 1997	CY 1998	CY 2002	REMARKS (For CY 2002 Case Finding)
TRENTO	12	11	11.26	76.65% Endemic Population Covered
1. Basa	--	--	16.25	84.36% Endemic Population Covered
2. Manat	--	--	19.09	Established Prevalence Rate for 2002
3. Tudela	--	--	7.93	90.39% Endemic Population Covered
4. San Roque	--	--	6.35	90.14% Endemic Population Covered
5. Kapatongan	12	11	11.74	56.74% Endemic Population Covered
6. San Isidro	--	--	4.90	86.62% Endemic Population Covered

Table 4. Schistosomiasis Prevalence Rates By Kato Stool Examination In The 5-14 Years Old Age Group
Bunawan and Trento, Agusan del Sur

Municipality	Prevalence Rate		Age Group	Total Positive	PR (%)
	1997	1998	5-14 Y.O.		2002
Bunawan	21	20	78	368	7.07
Trento	12	11	257	595	11.26

Table 5. Description of the Assessments or Activities to be Undertaken for Each Scheduled Visit

Scheduled visits	Assessment to be undertaken
Screening	• Demographic data<br>• Anthropometric measurements<br>• Treatment history<br>• Stool examination for *Schistosoma* eggs and other helminthes (will be done twice with two samples taken no more than 5 days interval as prescribed for baseline assessment)
D0	• Treatment<br>• Observation of the subject 4 hr after the treatment for safety assessment
D1 (+/- 4 hours)	• Interview of the subject for safety assessment
D21 (+/- 3 days)	• Stool examination for the determination of efficacy using cure rate and egg reduction rate as indicators<br>• Safety assessment<br>• Treatment of *S. japonicum* and soil transmitted helminths (STH) infected participants
D180 (+/- 7 days)	• Stool examination to determine reinfection<br>• Anthropometric measurements<br>• Treatment of *S. japonicum* and soil transmitted helminths (STH) infected participants
D360 (+/- 14 days)	• Stool examination to determine reinfection<br>• Anthropometric measurements<br>• Treatment of *S. japonicum* and soil transmitted helminths (STH) infected participants

Schematic diagram of the procedures

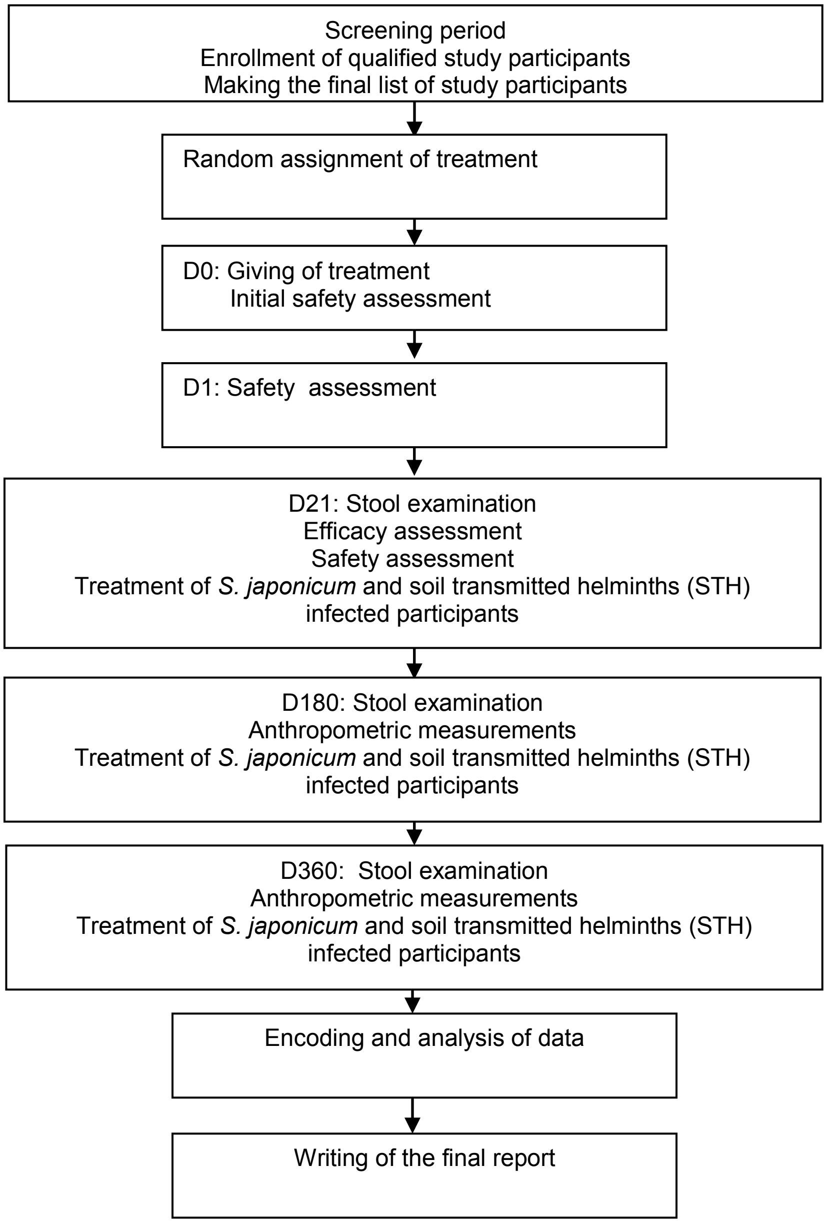

Table 6a. Classification Of Symptoms According To Severity

Classification	Impact to the life of the patient
1. Mild	• Causing easily-tolerated, minimal discomfort<br>• The patient is capable of performing common everyday functions<br>• No treatment required
2. Moderate	• Interferes with normal everyday functions<br>• Treatment may be required but not hospitalization
3. Severe	• Prevents normal everyday activities<br>• Treatment and/or hospitalization is required
4. Life-threatening	• Extreme limitation of activity, significant assistance required; significant medical intervention/therapy required, hospital or hospice care probable.

Table 6b. Grading scale for determining the severity of adverse events:

Clinical sign/symptom	grade 1	grade 2	grade 3	grade 4
Abdominal pain (2)	Mild	Moderate- no treatment needed	Moderate- treatment needed	Severe- hospitalization for treatment
Nausea (1)	Mild discomfort; maintains reasonable intake	Moderate discomfort; intake decreased significantly; some activity limited	Severe discomfort; no significant intake; activities limited	Minimal fluid intake
Vomiting (2)	1 episode/day	2-3 episodes/day	4-6 episodes/day	Greater than 6 episodes/day or intractable vomiting
Diarrhoea (2)	Slight change in consistency and/or frequency of stools	Liquid stools	Liquid stools greater than 4x the amount or number normal for this child	Liquid stools greater than 8x the amount or number normal for this child
Anorexia (2)	------------	Decreased appetite	Appetite very decreased, no solid food taken	No solid or liquid taken
Fever, oral (1)	37.7 – 38.5 C or<br>100.0 – 101.5 F	38.6 – 39.5 C or<br>101.6 – 102.9 F	39.6 – 40.5 C or<br>103 – 105 F	> 40.5 C or<br>> 105 F
Headache (1)	Mild, no therapy required	Transient, moderate; therapy required	Severe; responds to initial narcotic therapy	Intractable; required repeated narcotic therapy
Dizziness (3)	Not interfering with function	Interfering with function, but not interfering with activities of daily living	Interfering with activities of daily living	Bedridden or disabling
Allergic reaction (1)	Pruritus without rash	Localized urticaria	Generalized urticaria; angioedema	Anaphylaxis

Adapted from (1) WHO Toxicity Grading Scale, (2) Division of Microbiology and Infectious Diseases (DMID/NIH) Pediatric Toxicity Tables, and (3) National Cancer Institute (NCI) Toxicity Grading Scale.

Table 7 Classification of Symptoms According To Relationship to Treatment

Classification	Relationship to Treatment
1. Not related	• Event is clearly related to other factors such as the patient's clinical state, therapeutic intervention or concomitant therapy
2. Unlikely	• Event was probably produced by other factors such as the patient's clinical state, therapeutic intervention or concomitant therapy<br>• And does not follow a known response pattern to trial product
3. Possible	• Follows a reasonable temporal sequence from the time of product administration<br>• And/ or follows a known response pattern to the trial product<br>• But could have been produced by other factors such as the patient's clinical state, therapeutic intervention or concomitant therapy
4. Probable	• Follows a reasonable temporal sequence from the time of product administration<br>• And/ or follows a known response pattern to the trial product<br>• And could not have been produced by other factors such as the patient's clinical state, therapeutic intervention or concomitant therapy
5. Most probable	• Follows a reasonable temporal sequence from the time of product administration<br>• And/ or follows a known response pattern to the trial product<br>• And could not have been produced by other factors such as the patient's clinical state, therapeutic intervention or concomitant therapy<br>• And either occurs immediately following trial product administration, or improves on stopping the product
6. Insufficient data to assess	• There is not enough clinical and/or laboratory information to suggest the relationship of the event to the trial product

Adapted from: Karbwang and Pattou (2000). Standard operating procedures for clinical investigators

Table 8.1 Number of praziquantel tablets to be given to patients under 40 mg/kg regimen

Patient's Bodyweight	Number of tablets
13.1-15.9	1
16-18.9	1 ¼
19-22.9	1 ½
23-25.9	1 ¾
26-29.9	2
30-33.9	2 ¼
34-37.9	2 ½
38-40.0	2 ¾
41-44.9	3
45-48.9	3 ¼
49-52.9	3 ½
53-55.9	3 ¾
56-59.9	4
60-63.9	4 ¼
64-66.9	4 ½
67-70.9	4 ¾
71-75.9	5

Table 8.2 Number of praziquantel tablets to be given to patients under 60 mg/kg regimen

Patient's Bodyweight	Number of tablets
10	1
10-12.5	1 ¼
12.6-15	1 ½
15-17.5	1 ¾
17.6-20	2
20-22.5	2 ¼
22.6-25	2 ½
25-27.5	2 ¾
27.6-30	3
30-32.5	3 ¼
32.6-35	3 ½
35-37.5	3 ¾
37.6-40	4
40-42.5	4 ¼
42.6-45	4 ½
45-47.5	4 ¾
47.6-50	5
50-52.5	5 ¼
52.6-55	5 ½
55-57.5	5 ¾
57.6-60	6
