# Supplementary material for: A Multicentre Randomized Controlled Trial of the Efficacy and Safety of Single-Dose Praziquantel at 40 mg/kg vs. 60 mg/kg for Treating Intestinal Schistosomiasis in the Philippines, Mauritania, Tanzania and Brazil
Source: PLoS Negl Trop Dis. 2011 Jun 14;5(6):e1165. doi: 10.1371/journal.pntd.0001165 (PMC3114749; doi:10.1371/journal.pntd.0001165)
Supplement: Protocol S3 — Protocol of Mauritania. (PDF) [file pntd.0001165.s005.pdf]

PARTIE IV. DESCRIPTION DU PROJET

6-1: TITRE DU PROJET:

Comparaison de l'efficacité thérapeutique et la tolérance de 60mg /kg du Praziquantel en dose unique avec celle de 40mg/kg pour le traitement de la schistosomiase intestinale a *mansoni* dans un village de haute endémicité de la région du Trarza (Mauritanie)

6-2 : ETAT DES CONNAISSANCES ET MOTIVATION :

La schistosomiase demeure un véritable problème de santé publique dans plusieurs régions du monde notamment en Afrique, en Asie et en Amérique du sud.(OMS,93). En Mauritanie, La construction et la mise en service des barrages sur le fleuve Sénégal, a permis l'extension des aménagements hydro- agricoles qui visent à combler l'insuffisance alimentaire après les longues années de sécheresse. Des centaines de kilomètre de canaux d'irrigation et des milliers d'hectare de la riziculture ont été créés, constituant un habitat idéal pour les hôtes intermédiaires des maladies infectieuses, parasitaire notamment la schistosomiase. L'importance croissante des schistosomiases humaines, particulièrement la schistosomiase intestinale a *Schistosoma mansoni*, constitue un exemple de l'impact des barrages et aménagements hydro- agricoles sur la santé humaine. La prévalence globale de cette parasitose est passée de 9,7% en 1994 (Urbani *et al.*1997), a 23% en 1998 (Ouldabdallahi et *a, 1998l*). Alors qu'au niveau de certains villages (Breun) elle est hyperendemique avec une prévalence de 94% et une intensité de plus de 1 000 œufs/gr (Jacks et OuldAbdallahi, 2000). La schistosomiase urinaire était présente avant la construction des barrages, mais depuis leur mise en service sa prévalence est passé de 1,3% en 1981 (CEGET/OMS, 1987) à 24,7% en 1998 (Ouldabdallahi et *al* 1998) L-4-one ou le praziquantel (PZQ) est considéré comme médicament de choix pour traiter la schistosomiase durant les vingt dernières années dans la plupart des programmes de lutte contre la schistosomiase (DAVIS,1993). Cette préférence étant due essentiellement par ses effets indésirables minimes par rapport à son efficacité élevée contre la plupart des trématodes et les cestodes, ainsi qu à son coût raisonnable. Une dose unique de 40mg/kg suffit généralement pour donner des taux de guérison compris entre 60% et 90%, et une baisse de 90 à95% du nombre moyen d'oeufs excrétés (Katz.,1997, Gryseels.,1987 ;.Davis.,1993). En effet, les faibles taux de guérison qui ont été remarquées sur la rive gauche du fleuve Sénégal (stelma et al 1997), conduirait à une situation dangereuse, avec des très graves conséquences sur la morbidité ainsi que la propagation de la maladie dans cette zone. Selon plusieurs auteurs ce faible taux de guérison peuvent être attribué à la spécificité de la situation épidémiologique de la zone dont la transmission continu et intense, mes également de la forte charge parasitaire des patients, de leurs courte dure de réinfestation ainsi que la sous dosage du traitement même (Geerts & Gryseels2000).

Le projet que nous proposons a pour objectif de comparer l'efficacité thérapeutique et la tolérance de 60mg/kg du praziquantel en dose unique avec celle de 40mg/kg pour le traitement de la schistosomiase intestinale à *mansoni* dans un village de haute endémicité, situé sur la rive droite du Fleuve Sénégal (région du Trarza, Mauritanie).

6-3 : Objectifs de l'étude :

6-3-1-Objectif principal :

Comparer l'efficacité thérapeutique et la tolérance de 60mg/kg du praziquantel en dose unique par rapport à celle de 40mg/kg pour le traitement de la schistosomiase intestinale à *S. mansoni*

6-3-2 Objectifs secondaires :

- Déterminer les effets secondaires pouvant provenir de l'administration de 60mg/kg et de 40mg du praziquantel.
- Utiliser les informations pour l'élaboration des stratégies de traitement appropriées et l'amélioration de la prise en charge des cas.

6-4 : Méthodologie :

Site d'étude

Le village de Breun est situé à 14km à l'Ouest de Rosso chef lieu de la région du Trarza, sa population est d'environ 1600 habitants en majorité Wolof. Il dispose d'une école et d'un poste de santé. Les principales activités sont l'agriculture et la pêche. La prévalence de la schistosomiase à *S. mansoni* est de 94% et la saison de haute transmission s'étale d'avril en août (Stema, 1997).

Recrutement des sujets :

L'étude va concerner 218 sujets âgés de 10 à 19 ans vivants dans le village de Breun. Ce village a été suivi plusieurs fois par notre équipe de recherche et nous entretenons de bonnes relations avec les populations et les autorités sanitaires régionales. Après avoir expliqué nos objectifs aux populations et obtenu leur consentement pour participer à notre étude, les sujets subiront un examen physique et clinique par un médecin expérimenté pour détecter toutes éventuelles maladies ou critères d'inclusion et d'exclusion de l'étude. Les sujets non exclus après cette première étape, recevront des pots de prélèvement de selles portant leur nom avec un numéro d'identification. Ces pots seront recueillis le lendemain matin. Pour être acheminés dans le laboratoire de l'Hôpital

Régional de Rosso. Les examens parasitologiques seront effectués par la méthode de Kato-Katz (41.7mg de selle/lame) après avoir noté l'aspect macroscopique des selles (consistance, présence de mucus, ou du sang etc.). Les sujets inclus c'est-à-dire qui auront des densités parasitaires supérieures à 100 oeufs/g de selles feront l'objet d'un second prélèvement de selles dans les cinq jours suivant le premier prélèvement. Pour chaque prélèvement, deux lames seront préparées et la moyenne d'œufs par gramme de selles pour les quatre lames sera retenue comme valeur d'étude. Les sujets inclus seront classées au hasard en deux groupe puis recevront le traitement dans les 48 heures qui suivent le diagnostic parasitologique selon le protocole de l'étude. Cependant, les sujets positifs exclus recevront seulement un traitement au Praziquantel à la dose unique de 40mg/kg. Les sujets inclus avec d'autres parasitoses auront un traitement après le J21.Des formulaires contenants les variables suivantes : nom et prénom, âge, sexe, poids, adresse détaillée, date de prélèvement, date d'examen (labo), résultat clinique, résultat parasitologique, date d'inclusion, médicament administré, fournisseur (fabricant), date de l'administration, la dose, mode d'administration, date du prochain RV, éventuels effets indésirables apparus après l'administration du médicament.

Tableau récapitulatif des activités selon les visites.

Visite

Activité

J0

Traitement, détection des effets indésirables précoces du médicament (dans les 4 premières heures)

J1(24h+/-2h)

Examen physique et questionnaire sur les effets indésirables tardifs du médicament.

J21 (21j+/-2j)

Examen physique et questionnaire sur les effets indésirables du médicament, contrôle parasitologique pour évaluer l'efficacité du traitement (guérison ou taux de réduction des œufs).

6mois(180j+/-7j)

Contrôle parasitologique pour détecter la réinfection après traitement

12mois(365+/-14j)

Contrôle parasitologique pour détecter la réinfection après traitement

6-5 Sélection et élimination des individus

6-5-1 : Critères d'inclusions :

- Age de 10-19ans.
- Infection par *S. mansoni* >100 oeufs/g de selle
- Absence des maladies graves ou contre indication pour le traitement au praziquantel (hepato-splénomegalie ou allergie au PZQ)
- Consentement éclairé des patients ou de leurs parents. ainsi que l'acceptation de continuer jusqu'au fin d'étude

6-5-2 : Critères d'exclusion :

- Grossesse ou allaitement
- Traitements antérieurs au praziquantel ou autre antischistosomiase moins de 30 jours.

La prise d'un médicament pouvant interféré avec l'action du praziquantel (antibiotique).

6-5-3 : Critère d'éliminations :

- La prise d'un médicament antischistosomiase au cours de l'étude
- Quand le sujet décide de se retire de l'étude ou perdu de vue.
- Quand il/elle présente des maladies graves ou autres anomalies ne lui permettant pas de continuer. .

6-6 Traitement des sujets :

Le médicament testé est le Praziquantel (Shin Poong), dosé à 600 mg/comprimé en raison de 60mg/kg ou 40mg/kg de poids corporel.

Après avoir confirmé la positivité et l'intensité de l'infestation par la schistosomiase. Les sujets malades seront repartis au hasard en deux groupes bien identifiés :

1° groupe : les malades infestés par *Schistosoma mansoni* et qui seront traités par le praziquantel en raison 60mg/kg en dose unique

2° groupe : les malades infestés par *schistosoma mansoni* et qui seront traités par le praziquantel en raison

40mg/kg en dose unique.
Les sujets inclus positifs avec d'autres helminthiases seront traités par un antiparasitaire convenable après le 21 jours de leur traitement au praziquantel.

6-7 : évaluation de l'efficacité du traitement :
L'évaluation de l'efficacité du traitement sera faite par deux examens parasitologiques successive avec la méthode de Kato-Katz (41.7mg de selle/lame) à J21, et pour chaque prélèvement deux lames seront préparées et la moyenne d'œufs par gramme de selles pour les quatre lames sera retenue comme valeur d'étude.
1- La guérison chez les sujets qui on une absence d'œufs dans les selles à j21 et sur les 4 lames issu de 2 prélèvements successives.
2- Le taux de réduction des œufs chez les malades qui libèrent encore des oeufs après j21.Ce taux sera calculé par la formule: [1-nbre d'œufs après traitement/nbre d'œufs avant traitement] x 100
Les contrôles parasitologiques après 6 mois et 12 mois seront réservés pour évaluer le taux de réinfection chez les recrutés.

6-8 évaluations de la sécurité du traitement :
Rappelons que le Praziquantel a été administré au Brésil en raison de 60mg/kg en dose unique sans effets indésirables remarquables. Pour détecter toutes effets indésirables due a l'administration du praziquantel, la surveillance médicale sera assurée par un médecin expérimenté et tous les patients seront mis sous surveillance médicale pendant les 4 premières heures après l'administration du médicament afin de vérifier qu'il ne rejette pas le médicament et qu'il ne présente pas des effets secondaires, ainsi qu'à 24 heures après sur évaluation du questionnaire qui sera fait au malade. Les effets indésirables seront classés en effets indésirables précoces (survenus au cours des 4 premières heures) et tardifs quand ils surviennent entre 4 et 24 heures. En cas de persistance de manifestations sévères tel que, les signes allergiques, neurologiques, psychique ou cardiaques, les malades seront évacués immédiatement à l'hôpital régional de Rosso pour une meilleure prise en charge et pour également s'assurer que ces manifestation ne son pas des effets indésirables liées au traitement. Les effets secondaires légers et de courte durée notamment douleur abdominale, céphalée, prurit, anorexie, nausée, seront traiter par le médecin de l'étude sur place. Des médicaments d'urgence et des moyens de transport des malades seront disponibles sur place pour mieux sécuriser les patients. L'investigateur principal Coordonne toute les activités de l'étude il participe aux recrutements des patients, de leur suivi, de la gestion des données, etc. Il sera le responsable de la conformité de toutes les étapes de l'étude avec le respect strict du protocole et les instructions du TDR.
6-9 Statistique:
Les données seront traitées avec le logiciel Epi-info2002, et les tests statistiques par le Chi carré.
Le taux de réduction des oeufs sera calculé selon la formule :
[1-nbre d'œufs après traitement/nbre d'œufs avant traitement] x 100
Echantillonnages :
N= [P1 (100-P1) +P2 (100-P2)/ $(P1-P2)^2$] x7.85, On suppose que
*- Le traitement par 60mg/kg de praziquantel a une efficacité estimée de 80%
*- Le traitement au 40mg/kg du praziquantel a une efficacité estimée de 60%
Pour une efficacité de 80%, et un intervalle de confiance de 95%
N= 91 pour chaque groupe.
En considérant le taux de perte de vie a 20%, le total des patients recrutés sera 218 (109 pour chaque groupe)

6-11 : Contrôle de qualité :
10% des lames seront contrôlées par un laborantin expérimenté.

6-12 : Aspect éthique :
Ce projet a eu l'approbation des autorités concernés par le biais du Centre National d'Hygiène (CNH) agrées dans ce domaine ainsi que par le Programme National de Lutte Contre la Schistosomiase (voir documents attachés).
Les sujets seront recrutés volontairement et auront le droit de retirer leur engagement de participer dans l'étude a n'import quel moment. Les sujets examinés parasitologiquement positifs et qui ne remplissent pas les critères d'inclusions, seront traités par le praziquantel en raison de 40mg/kg ou par un autres médicament selon la cause de l'infestation.
Les sujets recrutés par l'étude seront pris en charge et bien suivis pendant la période de l'étude en cas de positivité avec d'autres helminthes les traitements seront administrés après le J21 du traitement antischistosomiase. Cependant, les sujets positifs exclus recevront seulement un traitement au Praziquantel à la dose unique de 40mg/kg.Des médicaments d'urgence et des moyens de transport des malades seront disponibles sur place pour mieux sécuriser les patients. Le consentement éclairé sera signé par les participants ou de leurs parent après avoir bien expliqué en langue locale toute la démarche de l'étude ainsi que les informations nécessaires tel que, la durée et l'objectif de l'étude qui vise a combattre une maladie dangereux dans la zone avec des conséquences sanitaires, socio économiques etc. Le consentement sera fini par la conclusion suivante:

j'ai bien lu touts les informations concernant le consentement, et j'ai obtenu les réponses sur toutes mes questions posées, et j'accepte de participer volontairement comme sujet dans cette étude, et j'ai compris que j'ai le droit de se retirer quand je veux sans que cela affect les prestations dont j'ai droit

Echéancier et chronogramme:
Le projet est prévu pour le mois d'octobre 2003, juste après l'hivernage suite à la période de haute transmission de la maladie et pour une durée de 15 mois.
Ould Abdallahi MOHAMED, Biologiste, CNH, tel. (222) 25 31 34, mobile (222) 642 02 99
Émail : ouldabdallahi@hotmail.com

Références bibliographiques

Davis A, 1993. Antischistosomal grugs and clinical practice. In human schistosomiasis, Jordan, P., Webbe, G.& Sturrock, R.F.(editors) wallingford : CAB Internatinal, pp. 367-404..

Guisse F, Polman K, Stelma FF, Mbaye A, Talla I, Niang M, Deelder A, A M, Ndir O& Gryseels B. 1997. therapeutical evaluation of two differnt dose regiments of praziquantel in a recent schistosoma mansoni focus in northern senegal. .. Am J Trop Med Hyg 56:511-514

Gryseels B, Nkulikyinka L.& Coosem M, H 1987. Field trils of praziquantel and oxamniquine for the treatement of schistosoma mansoni in burundi. Transactions of the royal society of tropical medecine and hygiene, 81,641-644.

Gryseels B, Mbaye.A, S.J.DeVlas, Stelma FF, F.Guisse, L.Van Lieshhout, D.Faye, M. Diop, A.Ly, L.A.Tuchuem-Tchuenté, D.Engels, and K.Polman. Are poor responses to praziquantel for the traitement of Schistosoma mansoni infections in Senegal due to resistance? An overview of the evidence. tropical medecine and international Health, V6, 11:864-873.

Gryseels B, Stelma FF, Talla I, Van Dam G, Polman K, Sow S, Diaw M, Sturrock RF, Doehring-Schwerdtfeger E, Kardorff R, Decem C, Niang M ; Deelder Am,1994. Epidemiolgy, immunology and chemotherapy of schistosoma mansoni infections in a recently exposed community in senegal. Trop Geogr Med 46 :209-219.

Jacks A., Ould Abdallahi M. - Possibilité de recherche sur les schistosomiases dans le Trarza ; enquête parasitologique parmi les écoliers de Breun, Rosso, Rapport de mission ; mai 2000, 5p.

Ould ABDALLAHI.M,WENE.XY, Rapport sur la prévalence de la bilharziose dans la ville de Rosso, année 1997, (Communication personnel, CNH)

Ould ABDALLAHI.M, Shu Jing. Rapport sur la prévalence des schistosomiases et le traitement de masse des écoliers dans la région du Trarza, année 1998,(Communication personnel, CNH)

Ould ABDALLAHI.M, Isselmou C, Chu Jing, Mohamed L. la prévalence des schistosomiases dans les régions du gorgol, l'assaba et guidimagha, Année 1998(document CNH).

Stelma FF, Tallal, Polman K,Niang M ; Sturrock Rf, Deelder Am, Gryseels B,1993. Epidemiology of schistosoma mansoni infection in a recentky exposed community in northern Senegal. Am J Trop Med Hyg 49 :701-706.
Stelma FF, Tallal, Verle P, ,Niang M ; Gryseels B,1994. Morbidity due to schistosoma mansoni infection in a recent focus in northern Senegal. Am J Trop Med Hyg 50:575-579

Stelma FF, Tallal, Sow S, Kongs A, Niang M, Polman K; Deelder AM, Gryseels B,1995. Efficacy and side effects of paziquantel in an epidemic focus of schistosoma mansoni.. Am J Trop Med Hyg 53:167-170.

Stelma FF, 1997. immuno-epidemiolgy, morbidity and chemotherapy in a community recently exposed to schistosoma mansoni infection- a stady in northern senegal. Phd thesis, Univercity of Leiden.

Urbani A., Toure A.,Hamad(Ouldabdallahi.M), Albonico M. et Coll. – Parasitoses intestinales et schistosomiases dans la vallée du fleuve Sénégal en République Islamique de Mauritanie. Med. Trop. 1997 ; 57 : 157 – 160.

WHO 1993. praziquantel in senegal schistosomiasis outbreak. TDR News, 42, 10-12.
