# Supplementary material for: A Multicentre Randomized Controlled Trial of the Efficacy and Safety of Single-Dose Praziquantel at 40 mg/kg vs. 60 mg/kg for Treating Intestinal Schistosomiasis in the Philippines, Mauritania, Tanzania and Brazil
Source: PLoS Negl Trop Dis. 2011 Jun 14;5(6):e1165. doi: 10.1371/journal.pntd.0001165 (PMC3114749; doi:10.1371/journal.pntd.0001165)
Supplement: Protocol S4 — Protocol of Brazil. (PDF) [file pntd.0001165.s006.pdf]

TRIAL PROTOCOL

6.1. General Information

Protocol title:

Evaluation of praziquantel dosage for treatment of schistosomiasis in highly endemic areas of Northeastern Brazil.

Principal Investigator:

Otávio Sarmento Pieri, Full Researcher, Dept. Biologia, Instituto Oswaldo Cruz, Fiocruz, Av. Brasil, 4365, Rio de Janeiro, CEP 21045-900, Brazil

Co-investigators:

- Constança Simões Barbosa - Senior Researcher, Oswaldo Cruz Foundation (Fiocruz): general supervision, in charge of training health agents from the target municipality (as part of the activities of the Service of Reference for Schistosomiasis Diagnosis of the CPqAM).
- Tereza Cristina Favre - Associate Researcher, Fiocruz: general supervision, in charge of the preliminary survey and subsequent stool surveys.
- Lilian Beck - Assistant Researcher, Fiocruz: field-work supervision, participation in the stool surveys.
- Frederico Guilherme Coutinho Abath, Senior Researcher, Fiocruz: in charge of the immunological study.
- Eridan Coutinho, Senior ResearcherFiocruz: in charge of the nutrition study.
  Silvia Montenegro, Senior Researcher, Fiocruz: in charge of the haematological and biochemical studies.
- Yara de Miranda Gomes, Senior Researcher, Fiocruz: participation in the haematological, biochemical and immunological studies.
- Ricardo Arraes de Alencar Ximenes, Associate Professor, (UFPE): epidemiological supervision.
- Ana Lúcia Coutinho, Associate Professor, Federal University of Pernambuco (UFPE): in charge of the clinical, ultrasonography and tolerability studies, treatment (medical expert). Address:Centro de Ciências da Saúde , Departamento de Medicina Clínica – UFPE, rua prof. Moraes Rêgo, s/n - Hospital das Clínicas, Cidade Universitária – Recife, CEP 50670-420, PE – Brazil, E-mail: mcoutinh@nlink.com.br, phones: (552181) 3221-0758 and (552181) 32718534.

Institution: Centro de Pesquisa Aggeu Magalhães (CPqAM), Fiocruz, Av. Moraes Rego, s/n, Cidade Universitária, CEP 50670-420, Recife, PE, Brazil. Rômulo Maciel Filho, Director.

Sponsor: UNDP/World Bank/WHO Special Programme for Research na Training in Tropical Diseases (TDR), Geneva, Switzerland.

6.2. Background Information

6.2.1. Name and description of the investigational product:

Praziquantel is provided as a tablet containing 600 mg of the active principle (2-Cyclohexilcarbonyl-1,3,4,6,7 11b hexahydropyrazino 2,1-a isoquinolin 4-one).

6.2.2. A summary of findings from clinical trials/previous studies that are relevant to the trial:

Our research group at Fiocruz provides the only information regarding the impact of yearly, population-based selective chemotherapy with praziquantel (single oral dose of 60 mg/kg) in Northeastern Brazil. Thus, data on replacing oxamniquine by praziquantel in a rural community from the sugar-cane zone of Pernambuco showed a

significant reduction in both prevalence and intensity of infection (Beck et al, 2001, Mem. Inst. Oswaldo Cuz, 96, Suppl.: 165-167). Complementary, unpublished data show impressive cure rates three months after treatment with praziquantel (74% in 1999, 71% in 2000, 100% in 2001 and 2002) as compared to oxamniquine (40% in 1997, 54% in 1998). As regards drug tolerability, no long-term side effects were noticed. However, compliance decreased with continuing treatment (from 97% in 1999 to 74% in 2000), what could be partially due to transient nausea and abdominal discomfort commonly reported after medication. These preliminary results indicate that praziquantel 60 mg/kg may be both efficacious and safe for use by control programmes in the highly endemic area of Northeastern Brazil. However, no field study has been done yet on the efficacy and safety of this dose as compared to the standard dose of 40 mg/kg, which may have low efficacy in some areas.

6.2.3. Summary of the known and potential risks and benefits, if any, human subjects:

TDR will provide data regarding the registration of praziquantel.

6.2.4. Description of and justification for the route of administration, dosage, dosage regimen and treatment period(s).

Praziquantel tablets will be administered orally in a single dose for the standard 40 mg/kg and 60 mg/kg regimens. Single dose is preferred because split dosage is operationally difficult to deal with in population-based control programmes.

6.2.5. Statement:

The trial will be conducted in compliance with the protocol, GCP and the applicable regulatory requirements.

6.2.6. Description of the population to be studied

An urban community named Sotave (municipality of Jaboatão, Recife Metropolitan Region, coastal zone of Pernambuco), with an estimated prevalence around 25% and no history of previous treatment with praziquantel was selected for the proposed study. In that zone, transmission is most likely to occur during the rainy season, which lasts from June to August; in contrast, transmission levels are minimal from September to March, as snail-host density at the water-contact sites is lowest (Pieri & Thomas, 1987, Mem. Inst. Oswaldo Cruz, 82, suppl IV: 197-201). Sotave has been regularly attended at the primary health care level through the Family Health Programme (FHP). Natural infection of *Biomphalaria glabrata*, the local snail host species, has reached 45% in some foci around houses according to a survey carried out in September 2002 by our research group. A stool survey by the municipal health authorities, carried out from December 2002 to January 2003, detected 480 (24%) positives out of 2,022 persons examined. In the 10-19 years group, 142 (35%) were positive among 410 examined. Of the 142 positives in this age group, 65 (46%) harboured at least 100 epg. The local FHP has estimated that approximately 6,500 persons currently live in Sotave, of which at least 1,230 are in the 10-19 yr age-range. This age group was chosen for the proposed study because it is particularly vulnerable to infection by *Schistosoma mansoni*. It is also the main age-group responsible for the contamination of the transmission foci.

6.2.7. References to literature and data that are relevant to the trial

The endemic area of schistosomiasis in Pernambuco State, Northeastern Brazil, comprises approximately 15,500 km^2. According to the 2000 census, more than 700,000 people live in rural communities in this area with practically no access to primary health care, sanitation or water supply. The National Health Foundation (Funasa) carried out successive control campaigns with oxamniquine in that area between 1979 and 1997. However, more than one fourth of the localities are still problematic, holding prevalence indices above 25% (Favre et al. 2001, Mem. Inst. Oswaldo Cruz, 97: 465-475.). Since 1998 schistosomiasis control has been under the Unified Health System (SUS), which involves both central and local authorities. The former are responsible for the technical guidelines and the latter, for their implementation. The technical guidelines by the Ministry of Health recommend that prevalence of infection in

the highly endemic, problematic localities should be reduced to less than 25% through treatment of the infected persons (identified by a diagnostic survey of the whole population) with an yearly single dose of praziquantel at the dosage of 60 mg per kg of body weight (mg/kg). Unfortunately, no municipality in the endemic area of Pernambuco has so far carried out sustained control measures against schistosomiasis under the SUS, due mainly to severe shortage of health workers and financial resources. Therefore, there is a dearth of information regarding the recently introduced use of praziquantel under real field conditions. Coastal communities in Pernambuco have been recently studied by our research group as they have become of increasing epidemiological importance due to poor living standards associated with ecologically favourable conditions for transmission (Barbosa et al, 1996 Rev. Saúde Publica, 30: 609-616; Barbosa et al, 1998, Mem. Inst. Oswaldo Cruz, 93, Suppl. 1: 265-266; Barbosa et al, 2000, Rev. Saúde Pública, 34: 337-341; Barbosa et al., 2001, Mem. Inst. Oswaldo Cruz, 96, Suppl.: 169-172; Barbosa et al, 2001, Cad. Saúde Pública, 17: 725-728; Favre TC et al, 2001, op.cit.).

6.3. Trial Objectives and Purpose

The primary objective of the proposed project is to evaluate the efficacy and tolerability of praziquantel 60mg/kg in the treatment of schistosomiasis, as compared with the standard praziquantel 40 mg/kg therapy in a representative community from the endemic area of schistosomiasis in Northeastern Brazil. The secondary objectives are: (i) to assess the tolerability/safety of each dose regimen through observed and reported side-effects; (ii) to assess the need for repeated treatment combined with other control measures through reinfection rates; (iii) to assess significant associations between the treatment outcome at 21 days and the ultrasonographic, anthropometric, haematological, biochemical and immunological features prior to treatment.

6.4. Trial Design

6.4.1. primary and secondary endpoints

The primary endpoint to be measured during the trial will be the cure rate at 21 days after treatment. The secondary endpoints will be (i) egg reduction rates at 21 days after treatment, (ii) observed side-effects for one hour after treatment (iii) reported side-effects at 24 h and 21 days after treatment, (iv) reinfection rates at six months and 12 months after treatment.

6.4.2. Type/design of trial

Timetable of procedures and stages

SEPTEMBER 2003	
1.	**Sketch map of the study area and snail survey** *(personnel: PI, trainee, two CS and two HA[1]).*
2.	**Application of questionnaire (1,400 households)** *(personnel: PI, trainee, two CS and four HA).*

[1] PI, principal investigator; CS, collaborating scientists; HA, health agents)

OCTOBER 2003
**3. Screening visit** *(personnel: PI, trainee, three CS, two HA and five survey teams[2])*. An estimated total of 1230 children aged 10-19 yrs will be interviewed at their homes. Up to 300 interviews and stool exams will be made per day, as follows:<br><br>**06/10/03**: screening of children # 1 to 300<br>**07/10/03**: screening of children # 301 to 600 / stool collection and processing of first sample from children # 1 to 300.<br>**08/10/03**: screening of children # 601 to 900 / stool collection and processing of first sample from children # 301 to 600.<br>**09/10/03**: screening of children # 901 to 1230 / stool collection and processing of first sample from children # 601 to 900.<br>**10/10/03**: stool collection and processing of first sample from children # 901 to 1230.<br>**11/10/03**: stool collection and processing of second sample from children # 1 to 300.<br>**12/10/03**: stool collection and processing of second sample from children # 301-600.<br>**13/10/03**: stool collection and processing of second sample from children # 601-900.<br>**14/10/03**: stool collection and processing of second sample from children # 901 to 1230.<br>**15/10/03**: stool collection and processing of second sample from children # 1001 to 1230.
**4. Enrolment visit** *(personnel: PI, medical expert, physician, trainee, three CS and four HA)*. An estimated total of 208 children with epg ≥ 100 will be invited for enrolment at the local Health Centre and interviewed for fulfilment of the inclusion/exclusion criteria. Those who fulfil the criteria for enrolment will proceed to blood collection, USG examination, praziquantel treatment as well as post-treatment observation for four hours. Up to 60 patients will be enrolled per day (at seven days after the screening visit), as follows:<br><br>**13/10/03**: enrolment, blood collection, USG, treatment and post-treatment observation of children screened in 06/10/03<br>**14/10/03**: enrolment, blood collection, USG, treatment and post-treatment observation of children screened in 07/10/03<br>**15/10/03**: enrolment, blood collection, USG, treatment and post-treatment observation of children screened in 08/10/03<br>**16/10/03**: enrolment, blood collection, USG, treatment and post-treatment observation of children screened in 09/10/03
**5. Day-1 vist** *(personnel: PI, medical expert, physician, trainee, three CS and twor HA)*. The patients will return to the local Health Centre at 24 ± 2 hours after treatment for a follow-up interview about adverse events, as follows:<br><br>**14/10/03**: follow-up interview of children enrolled and treated in 13/10/03<br>**15/10/03**: follow-up interview of children enrolled and treated in 14/10/03<br>**16/10/03**: follow-up interview of children enrolled and treated in 15/10/03<br>**17/10/03**: follow-up interview of children enrolled and treated in 16/10/03

NOVEMBER 2003
**6. Day-21 visit** *(personnel: PI, medical expert, physician, trainee, three CS, two HA and one survey team)*. The patients will be visited at their homes at 21 ± 2 days after treatment for a follow-up interview about adverse events.. Up to 60 interviews and stool exams will be made per day, as follows:<br><br>**01/11/03**: follow-up interview of children treated in 13/10/03<br>**02/11/03**: follow-up interview of children treated from 13/10/03 to 14/10/03 / stool collection and processing of first sample from children visited in 01/11/03.<br>**03/11/03**: follow-up interview of children treated from 13/10/03 to 15/10/03 / stool collection and processing of first sample from children visited in 02/11/03.<br>**04/11/03**: follow-up interview of children treated from 13/10/03 to 16/10/03 / stool collection and processing of first sample from children visited in 03/11/03.<br>**05/11/03**: follow-up interview of children treated from 13/10/03 to 16/10/03 / stool collection and processing of first sample from children visited in 04/11/03.<br>**06/11/03**: follow-up interview of children treated from 14/10/03 to 16/10/03 / stool collection and processing of first sample from children visited in 05/11/03 / stool collection and processing of second sample from children visited in 01/11/03.<br>**07/11/03**: follow-up interview of children treated from 15/10/03 to 16/10/03 / stool collection and processing of first sample from children visited in 06/11/03 / stool collection and processing of second sample from children visited in 02/11/03.<br>**08/11/03**: follow-up interview of children treated in 16/10/03 / stool collection and processing of first sample from children visited in 07/11/03 / stool collection and processing of second sample from children visited in 03/11/03.<br>**09/11/03**: stool collection and processing of first sample from children visited in 08/11/03 / stool collection and processing of second sample from children visited in 04/11/03.<br>**10/11/03**: stool collection and processing of second sample from children visited in 05/11/03.<br>**11/11/03**: stool collection and processing of second sample from children visited in 06/11/03.<br>**12/11/03**: stool collection and processing of second sample from children visited in 07/11/03.<br>**13/11/30**: stool collection and processing of second sample from children visited in 08/11/03.

APRIL 2004
**7. Month-6 visit** *(personnel: PI, trainee, three CS, two HA and one survey team)*. The patients will be visited at their homes at 180 ± 7 days after treatment. They will be asked to provide two stool samples in the interval of five days. The stool vials will be left for collection in the following day.

OCTOBER 2004
**8. Month-12 visit** *(personnel: PI, trainee, three CS, two HA and one survey team)*. The patients will be visited at their homes at 360 ± 14 days after treatment. They will be asked to provide two stool samples in the interval of five days. The stool vials will be left for collection in the following day.

6.4.3. Measures taken to minimize/avoid bias

(a) Randomization: Randomization will be generated in blocks of 4, in a ratio of 1:1 for each drug regimen, Praziquantel 40mg/Kg or Praziquantel 60 mg/Kg. Sealed and numbered envelopes containing the treatment regimen for each sequential patient to be enrolled will be provided to the Investigator. The envelopes containing the codes will be kept in a locked cabinet and one designated person in the site will be responsible for them. This same person will be responsible for opening the envelope and verifying the corresponding treatment once a new patient is enrolled in the study. Each patient will be assigned a unique Study I.D. number (ID#). This ID# will be assigned in sequential order and must be recorded in the Screening Log. The treatment will be provided to the patient according to the Praziquantel regimen assigned by the randomization code and the patient's weight (see Table for dosages below). An electronic, password-protected copy of the randomization codes will be kept at TDR, Geneva. The opened envelopes should be signed, dated, resealed and kept in the locket cabinet.

(b) Blinding: the person who calculate the dosage of praziquantel to be given and provide de medication to the patients will not be the person who will evaluate safety; persons involved in data collection of any kind will have no knowledge of the subject's group

6.4.4. Trial treatment and dosage of the investigational product

Praziquantel is provided in tablets of 600mg with 3 scores, which may be divided in 4 segments of 150 mg each. The amount of drug to be given can be adjusted to the patient's bodyweight by breaking the tablets segments. Table 1 below will be used as a reference for number of tablets to be given according to treatment regimen and patient's bodyweight.

Table 1. . Number of praziquantel tablets to be given to patients under the 40mg/kg and 60mg/kg regimens. In this table, 13-15 means 13.1 kg to 15.9 kg. The same applies to the other weight ranges

**40 mg/kg regimen**		**60 mg/kg regimen**	
**Bodyweight (kg)**	**Number of tablets**	**Bodyweight (kg)**	**Number of tablets**
13-15	1	10	1
16-18	1 ¼	10 - 12.5	1 ¼
19 - 22	1 ½	12.6 - 15	1 ½
23 - 25	1 ¾	15 - 17.5	1 ¾
26 - 29	2	17.6 - 20	2
30 - 33	2 ¼	20 - 22.5	2 ¼
34 - 37	2 ½	22.6 - 25	2 ½
38 - 40	2 ¾	25 - 27.5	2 ¾
41 - 44	3	27.6 - 30	3
45 - 48	3 ¼	30 - 32.5	3 ¼
49 - 52	3 ½	32.6 - 35	3 ½
53 - 55	3 ¾	35 - 37.5	3 ¾
56 - 59	4	37.6 - 40	4
60 - 63	4 ¼	40 - 42.5	4 ¼
64 - 66	4 ½	42.6 - 45	4 ½
67 - 70	4 ¾	45 - 47.5	4 ¾
71 - 75	5	47.6 - 50	5
		50 - 52.5	5 ¼
		52.6 - 55	5 ½
		55 - 57.5	5 ¾
		57.6 - 60	6

[2] Each survey team will be composed of one leader, two laboratory technicians and one auxiliary worker, trained to collect and process parasitological data from at least 60 subjects per day

6.4.5. Description and time frame of subject participation

Descripiton of subject participation

In September 2003, trained health agents will make a sketch map of the village (depicting the location of houses, main watercourses, snail habitats and water-contact sites) and apply a household questionnaire to obtain personal data from residents, as well as to record sanitary standards of the houses and water-contact reported behaviour as described by Pieri et al (1998, Mem. Inst. Oswaldo Cruz, 93, Suppl 1: 259-264).

In October 2003, all residents between 10 and 19 yrs of age will be interviewed at their homes regarding the fulfilment of the inclusion/exclusion criteria. Those who fulfil the criteria will be given stool vials and asked to provide stool samples (two samples within 5-day intervals, two slides each) that will be collected at their homes in the following day, stored in heat-proof containers and taken to the premises of the Service of Reference for Schistosomiasis Diagnosis of the CPqAM to be promptly prepared for examination by the Kato-Katz method (Katz et al, 1972, Rev. Inst. Med. Trop. Sao Paulo, 14: 397-400) aiming to identify positive cases and estimate individual egg load. Only those harbouring at least 100 epg who are able and willing to follow-up and provide a written informed consent will participate in the study. In the enrolment visit the consenting subjects will be examined by the physician, who will record individual information including the following aspects (i) anthropometry (height, weight) – this will enable to characterize the subjects according to the nutritional status (Coutinho et al, 1997, Mem. Inst. Oswaldo Cruz, 92: 707-715); (ii) ultrasonography – this will enable to identify and categorize subjects with periportal thickening or fibrosis (Richter et al, 2001, Mem. Inst. Oswaldo Cruz, 96, Suppl., 151-156). The subjects will also provide one venous blood sample of 5 ml for an evaluation of: (i) haematological; (ii) biochemical (serum bilirubin, alkaline phosphatase, urea, creatinin, serum asparte aminotransferase, serum alamine aminotransferase); (iii) immunological (cytokine and antibody responses) features.

Table 2. Normal range values for haematological and biochemical parameters. Male (m), female (f)

Lab test	Parameters	Normal range values
Haematology	Haemoglobin	13.0 to 17.8 g/dl (m)<br>12.0 to 16.0 g/dl (f)
	Haematocrit	39% to 53% (m)<br>35% to 47% (f)
	Erytrocyte count	$5.5x10^5$ to $6.1x10^5$/ mm^3 (m)<br>$4.0x10^5$ to $5.5x10^5$/ mm^3 (f)
	Leucocyte count	4,000 to 11,000/mm^3
	Neutrophile	2,000 to 7,000/mm^3
	Eosinophile	40-660/mm^3
	Basophile	0 to 110/mm^3
	Lymphcyte	800 to 4,400/mm^3
	Monocyte	120 to 1,100/mm^3
Biochemistry	Serum bilirubin: total<br>direct<br>indirect	0.2 to 0.8 mg%<br>0.1 to 0.4 mg%<br>0.2 to0.7 mg%
	Alkaline phosphatase	62 to 176 u/l (m)<br>56 to 155 u/l (f)
	Urea	10 to 45 mg/dl
	Serum creatinin	0.5 to 1.2 mg/dl
	Serum asparte aminotransferase	10 to 30 u/l
	Serum alamine aminotransferase	5 to 30 u/l

Soluble egg antigen (SEA) and soluble worm antigenic preparation (SWAP) will be prepared as described (Pearce et al., 1991) and will be used at a final concentrations of 20 and 50 ug/ml respectively. Miristate Phorbol Acetate (PMA, 50 ng/ml) and Ionomicyn (IONO, 1 ug/ml) will be used as positive controls.

The whole peripheral blood (5 ml) will be drawn with heparin (10 U/ml), 2 ml will be separated by centrifugation and the plasma will be frozen to be used for biochemical and IgG antibodies tests. The other 3 ml of whole blood will be diluted 1:3 in RPMI plus penicillin (100 U/ml) and streptomycin (100 ug/ml), cultures will be maintained in a

humidified atmosphere with 5% CO_2. The supernatants will be collected at 48 h and immediately frozen and stored at -70°C for subsequent determination of cytokine production.

The measurement of cytokines will be performed by ELISA using specific capture and detection mAbs following previously published protocols (Williams et al., 1994). Briefly, flat-bottom plates (Immulon 4; Dynatech, Chantilly, VA) will be coated with appropriate antibodies for the detection of IFN-γ (mouse anti-human #M700-A at 2 ug/ml, Endogen), and IL-4 (mouse anti-human IL-4 8D4 #18651D at 1 ug/ml, Endogen); anticorpo biotinilado anti IL-4 humana, #18502D. The plates will be blocked with 5% evaporated milk in PBS at 37°C for 2 h and washed four times in 0.05% Tween-20 in PBS. Test samples and serial dilutions of standards will be diluted in 1% BSA/0.05% Tween-20 in PBS (ELISA diluent), added to the plates, and incubated overnight at 4°C. Biotinylated second step antibodies (polyclonal rabbit anti-h IFN-g, Endogen, P-700, 1:1,000 Endogen; anti-human anti IL-4 #18502D, Endogen) will be diluted in ELISA diluent and added to the appropriate plates and incubated for 2 h at 37°C. The plates will be washed and streptavidin-horseradish peroxidase conjugate (1:750) will be added for the detection of IFN-γ and IL-4. After a final wash, the cytokines plates will be developed with the ABTS one-step reagent (Kirkegaard and Perrry, Gaithersburg, MD) for approximately 20 min. The reactions will be stopped with 10 ul of 10% SDS.

Production of IFN-g and IL-4 will be quantified as previously described (Montenegro et al, 1999, J. Infect. Dis., 179:1502-1514), in order to evaluate the antigen specific Th1 and Th2 responses, respectively. IgG antibodies to antigens of S.mansoni (SEA and SWAP) will be also tested to have a general idea of the humoral immune response at the time of treatment. IgG (total IgG) antibody response to SEA (soluble egg antigens) and SWAP (soluble worm antigenic preparation) will be evaluated by ELISA (Hagan et al. 1991). Microtiter plates (Nunc-Immuno Plates, MaxiSorp, 96 wells, Nalge Nunc International Corporation) will be coated with 5 μg/ml antigens (100 μl/well) diluted in 0.05 M Na_2CO_3 buffer, pH 9.6 and incubated overnight at 4°C. The plates will be blocked for 2 h with PBS-Tween 20 (0.05%) (PBS-Tw) containing 5% defatted milk (Nestlé), prior to incubation of 100 μl of sera diluted (1:100) in PBS-Tw (2 h, room temperature). The bound antibodies will be detected with peroxidase-conjugated goat anti-human IgG (whole molecule) (Sigma Chemical Co, St. Louis, USA). The immune complexes will be revealed by addition of orthophenyldiamine-OPD and H_2O_2. Optical density (OD) will be measured at 490nm. The cutoff will be established using the OD mean of the negative controls + 2 standard deviations (SD).

Subjects will be randomly allocated to either Group A (single 40 mg/kg dose) or Group B (single 60 mg/kg dose). Praziquantel tablets will be provided by TDR. For four hours after treatment the subjects will be observed for safety assessment, and one day and 21 days after treatment they will be interviewed about the following symptoms related to praziquantel side-effects (Guisse et al, 1997, Am. J. Trop. Med. Hyg., 56: 511-514, 1997): abdominal pain, diarrhea, vomiting, nausea, drowsiness, general malaise, edema, skin rash, urticaria, myalgia, hearthburn, fever, dizziness and headache. The follow-up visits will be at 21 days, six months and 12 months after treatment, when the subjects will be surveyed by the Kato-Katz method (two stool samples at five-day interval, two slides each.

Table 3. Time frame of subject participation

Visit	Time range	Month/yr	Activities
Screening	7 days before treatment	Oct/03	1. Screening interview<br>2. Stool collection (two samples at five-day interval)
Enrolment	Day 0	Oct/03	1. Enrolment interview<br>2. Blood collection<br>3. Ultrasonography<br>4. Praziquantel treatment<br>5. Observation of adverse events for four hours after treatment

Day 1	24± 2 h after treatment	Oct/03	1. Follow-up interview for adverse events
Day 21	21± 2 days after treatment	Nov/03	1. Follow-up interview for adverse events, recent illness and medication<br>2. Stool collection (two samples at five-day interval)<br>3. Mebendazole treatment for other helminths
Month 6	6 months ± 7 days after treatment	Apr/04	1. Stool collection (two samples at five-day interval)<br>2. Mebendazole treatment for other helminths
Month 12	12 months ± 14 days after treatment	Oct/04	1. Stool collection (two samples at five-day interval)<br>2. praziquantel and mebendazole treatment for *S. mansoni* and other helminths

6.4.6. Stopping rules and discontinuation criteria

(a) Individual withdrawal: the subject will be withdrawn from the study if he/she presents and Serious Adverse Event, or if he/she takes any other anti-schistosomal medication during the trial, or if/he/she presents any illness or condition that makes further participation impossible, or if he/she decides to not participate.

(b) Criteria for stopping the trial: Is it unlikely that an untoward event would occur, given that Funasa is already using 60 mg/kg praziquantel in control programmes, and no serious adverse events has been reported. However, the Data Safety Monitoring Board (DSMB) will determine, if and when serious adverse effects occur and have a probable causal relationship with the treatment, that the trial must be stopped.

6.4.7. Accountability procedures for the investigational product

The investigational product will be safely stored at the premises of the Service of Reference for Schistosomiasis Diagnosis of the CPqAM under laboratory temperature (25±2 °C). The principal invsetigatort wil be responsible to account for used and unused study supplies, register the amount of drug received, dispensed and returned medication.

6.4.8. Maintenance of codes and procedures for breaking codes

TDR will provide this information

6.4.9. Identification of data to be recorded on the CRFs

To be decided after TDR has the forms, source documents and CRF

6.5. Selection and Withdrawal of Subjects

6.5.1. Inclusion criteria

Persons with 10-19 years of age harbouring at least 100 epg who are able and willing to follow-up and provide a written informed consent will participate in the study

6.5.2. Exclusion criteria

The following cases will be excluded from the proposed study: pregnancy or lactation; acute or chronic severe diseases including hepato-splenic schistosomiais; use of praziquantel in the last 30 days; previous history of adverse reaction associated with praziquantel; current use of other medication that may affect the results of the present trial, as antibiotics and cortisteroids.

6.5.3. Withdrawal criteria

Same as individual withdrawal above

6.6. Treatment of Subjects

6.6.1. Treatment

Praziquantel will be administered at a single oral dose of either 40mg/kg (group A) or 60 mg/kg (group B). Subjects of both groups will be observed for four hour after medication and assessed for reported side-effects at the following day and at the 21st day. The period of follow-up will include a stool survey at six months and 12 months after treatment.

6.6.2. Medication permitted

Individuals who used praziquantel in the previous 30 days, and individuals currently in antibiotics or cortisteroids will be excluded from the study. Individuals who take any anti-schistosomal drug during the period of follow-up will be exclude from further analysis.

Patients diagnosed for other helminths by Kato-Katz examination will be treated with 100mg mebendazole administered twice a day over three consecutive days, provided after the Day-21 visit.

6.6.3. Procedures for monitoring subject compliance

Treatment will be administered under observation.

6.7. Assessment of Efficacy

6.7.1. Specification of the efficacy parameters

Cure rate and egg reduction rate at 21 days after treatment

6.7.2. Methods and timing for assessing, recording and analysing of efficacy parameters

Both cure rate and egg reduction rate will be assessed by the Kato-Katz method, comparing the egg counts from a screening stool survey (two samples within 5-day intervals, two slides each) of the subjects with the egg counts from a follow-up stool survey (also two samples within 5-day intervals, two slides each) at 21 days after treatment. Cure rate will be estimated as the proportion of subjects who become negative 21 days after treatment. . Egg count reduction will be estimated through the following formula: [1 - (epg2 / epg1) x 100] where epg1 and epg2 are the geometric mean of $\log_{10}$ transformed (x+1) numbers of eggs per gram of faeces at the screening survey and the 21st day post-treatment survey.

6.8. Assessment of Safety

6.8.1. Specification of safety parameters

Occurrence of the following symptoms following praziquantel administration: abdominal pain, diarrhea, vomiting, nausea, drowsiness, general malaise, edema, skin rash, urticaria, myalgia, hearthburn, fever, dizziness and headache.

6.8.2. Methods and timing for assessing, recording and analysing of safety parameters

The occurrence of symptoms associated with praziquantel side-effects will be assessed by observation for four hours after treatment, and by questionnaire at one day and 21 days after treatment, according to the CRF enclosed.

6.8.3. Procedures for eliciting reports of and for recording and reporting adverse event and intercurrent illness

Adverse events and intercurrent illness will be reported at the appropriate forms (see CRF enclosed) and expedited and sent to the Clinical Monitor or Sponsor within 24 hours of identification.

6.8.4. The type and duration of the follow-up after adverse events

The adverse events will be taken care of and followed-up until their complete disappearance.

6.9. Statistics

6.9.1. A description of the statistical methods to be employed

Contingency-table analysis will be used to evaluate significant differences in cure rates and in the proportion of reported side-effects symptoms between groups A (40 mg/kg) and B (60 mg/kg). Analysis of variance will be used to evaluate significant differences in the log-transformed values of egg counts between groups A (40 mg/kg) and B (60 mg/kg). Multivariate analysis will be used to evaluate associations between the data from the anthropometry, ultrasonography, haematology, biochemistry and immunology surveys and those from the parasitological surveys.

6.9.2. Number of subjects planned to be enrolled

Sample size was calculated by using EPI-Info Software Package for an 80% chance of detecting a difference between the two given proportions at the 0.05 level of significance (two tailed). For this calculation, the expected

efficacy referring to cure rate was considered as 60% for praziquantel 40 mg/kg and 80% for praziquantel 60 mg/kg. As a result, 91 observations by group would be needed in each sample, totaling 182 subjects. However, all the patients fulfilling the inclusion/exclusion criteria will be used in the study to account for a potential lost-to-follow-up of 20%.

6.9.3. Level of significance to be used

The level of significance will be 0.05 (two-tailed)

6.9.4. Criteria for the termination of the trial

Analysis will be performed after all data has been collected.

6.9.5. Procedure for accounting for missing, unused and spurious data

Missing values will be analysed through the method of regression substitution available in the Systat 10 (SPSS Inc.) statistical package, using multiple linear regression to impute estimates for missing values.

Outlier values will be identified by the Systat 10 statistical package, using automatic diagnostics to verify that the data meet the underlying assumptions for ANOVA, Linear Regression, and General Linear Models (GLM). One analysis with the actual values and other analysis reducing the outlier effect will be performed, and the differences between their results will be discussed.

6.9.6. Procedures for reporting deviations from the original statistical plan

No deviations from the original statistical plan are expected

6.9.7. Selection of subjects to be included in the analysis

All patients that fit to the inclusion/exclusion criteria will be used in the study to account for the potential lost-to-follow-up.

All patients who received the treatment and were followed until day 21 will be included in the safety analysis.

All patients who received the treatment and provided stool samples at day 21 will be included in the efficacy analysis.

All patients who received the treatment and provided stool samples at day 21 as well as at Month 6 and/or Month 12 will be included in the reinfection analysis.

All patients who received the treatment and provided stool samples at day 21 and/or thereafter will be included in the anthropometric analysis.

All patients who provided blood samples, received the treatment and provided stool samples at day 21 and/or thereafter will be included in the haematological, biochemical and immunological analyses.

All patients who underwent USG examination, received the treatment and provided stool samples at day 21 and/or thereafter will be included in the ultrasonography analysis.

6.10. Direct Access to Source Data/Documents

The principal investigator and the institution will permit trial-related monitoring, audits, IRB/IEC review and regulatory inspection, providing direct access to source data/documents.

6.11. Quality Control and Quality Assurance

For Kato-Katz diagnosis, 10% of the slides will be read by an experienced parasitologist to evaluate the results observed by the microscopist. The microscopists of the Service of Reference for Schistosomiasis Diagnosis of the CPqAM has achieved 98% of reliabilty under routine conditions.

Anthropometric measurements will be checked by a senior researcher and those from USG will be checked by the medical expert.

Haematological, biochemical and immunological measurements will follow the standard procedures for quality control.

For database, the data will be double entered by two different persons and validated before analysis.

Ethics

The proposed research follows the Guidelines and Regulations for Research Involving Humans Beings (resolution 196/1996 of the National Health Council), and complies with the principles of the Declaration of Helsinki, amended as stated in Section II, item 15.1. The potential subjects will be orally informed about the aims of the research, procedures, risks, possible discomfort, benefits, expected duration as well as their freedom to withdraw from the study at any time. A member of the project staff will ascertain that the potential subjects will understand this information and give their consent freely. Both the staff member and each individual subject (or guardian in case of subjects under 18 years of age) will sign a written term of consent, which is enclosed as a separate appendix.

6.12. Data Handling and Record Keeping

The Principal Investigator will keep all files and documents in a locked cabinet, in a secure area accessible only to the investigator and authorized study staff. The investigator file and associated source documents, as well as patients identification codes will be retained for 15 years after completion of the trial. Written approval from the Sponsor (TDR) will be obtained prior to destroying records.
