# Supplementary material for: A Multicentre Randomized Controlled Trial of the Efficacy and Safety of Single-Dose Praziquantel at 40 mg/kg vs. 60 mg/kg for Treating Intestinal Schistosomiasis in the Philippines, Mauritania, Tanzania and Brazil
Source: PLoS Negl Trop Dis. 2011 Jun 14;5(6):e1165. doi: 10.1371/journal.pntd.0001165 (PMC3114749; doi:10.1371/journal.pntd.0001165)
Supplement: Table S1 — Eggs per gram at Day 180 and Day 360. (DOC) [file pntd.0001165.s007.doc]

Table S1. Eggs per gram at Day 180 and Day 360.

|  |  |  | 40 mg/kg | | |  | 60mg/kg | | |  | | Repeated ANOVA | |  | | Repeated ANOVA | |  | |  |
| --- | --- | --- | --- | --- | --- | --- | --- | --- | --- | --- | --- | --- | --- | --- | --- | --- | --- | --- | --- | --- |
|  |  |  | N | Mean | ± SD |  | N | Mean | ± SD |  | | group/time*group | |  | | group/time*group | |  | |  |
| Day 180 | Philippines |  | 85 | 17.95 | ± 58.72 |  | 85 | 95.22 | ± 209.39 | |  | | 0.8305/0.9326 | |  | |  | |  | |
|  | Brazil |  | 90 | 5.80 | ± 15.38 |  | 87 | 1.65 | ± 8.32 |  | | 0.0776/0.0131 | |  | |  | |  | |  |
|  | Mauritania |  | 85 | 9.23 | ± 60.23 |  | 89 | 26.88 | ± 149.54 |  | | 0.3973/0.5028 | |  | |  | |  | |  |
|  | Tanzania |  | 119 | 26.35 | ± 78.07 |  | 112 | 18.73 | ± 59.55 |  | | 0.0240/0.0617 | |  | |  | |  | |  |
|  | ALL |  | 379 | 15.75 | ± 60.00 |  | 375 | 19.50 | ± 95.16 |  | | 0.1337/0.0.0353** | |  | |  | |  | |  |
|  |  |  |  |  |  |  |  |  |  |  | |  | |  | |  | |  | |  |
| Day 360 | Philippines |  | 85 | 30.78 | ± 106.51 |  | 87 | 51.95 | ± 148.33 |  | | 0.0822/0.0677 | |  | | 0.0671/0.1906 | |  | |  |
|  | Brazil |  | 90 | 19.46 | ± 57.17 |  | 87 | 23.10 | ± 83.56 |  | | 0.1002/0.0370 | |  | | 0.0113/0.0094 | |  | |  |
|  | Mauritania |  | 85 | 12.06 | ± 58.76 |  | 89 | 31.52 | ± 124.25 |  | | 0.1959/0.6155 | |  | | 0.3079/0.5832 | |  | |  |
|  | Tanzania |  | 119 | 61.66 | ± 147.00 |  | 112 | 65.25 | ± 144.78 |  | | 0.8297/0.7074 | |  | | 0.5919/3859 | |  | |  |
|  | ALL |  | 379 | 48.04 | ± 138.09 |  | 375 | 44.38 | ± 129.64 |  | | 0.1793/0.0419** | |  | | 0.5601/0.2267** | |  | |  |
| * Random Coefficient Model was used to account for the country | | | | | | | | | |  | | **country difference p=<0.0001 | | | | | | | |  |

Repeated Anova results are presented for the comparison of Baseline and Day 180, Baseline and Day 360 and longitudinally accounting of Baseline, Day 21, Day 180 and Day 360 values.
