# Supplementary material for: A Multicentre Randomized Controlled Trial of the Efficacy and Safety of Single-Dose Praziquantel at 40 mg/kg vs. 60 mg/kg for Treating Intestinal Schistosomiasis in the Philippines, Mauritania, Tanzania and Brazil
Source: PLoS Negl Trop Dis. 2011 Jun 14;5(6):e1165. doi: 10.1371/journal.pntd.0001165 (PMC3114749; doi:10.1371/journal.pntd.0001165)
Supplement: Table S2 — Post-day 21 reinfections. (DOC) [file pntd.0001165.s008.doc]

|  |  |  | Philippines |  | Brazil |  | Mauritania |  | Tanzania |  | ALL |  |
| --- | --- | --- | --- | --- | --- | --- | --- | --- | --- | --- | --- | --- |
| 40 mg/kg | Median^#^ | 95%CI | 344.00 | [343;347] | 343.00 | [338; -] | 393.00 | [373;393] | 342.00 | [169;343] | 361.00 | [348.366] |
|  | Day 360  Rate@ | Day 360 95%CI | 37.50 | [28.1.48.8] | 40.20 | [30.9;51.0] | 7.70 | [3.7;15.4] | 47.20 | [38.8;56.4] | 34.30 | [29.8;39.3] |
|  | Overall Survival  Day |  | 369 |  | 364 |  | 394 |  | 370 |  | 394 |  |
|  | Overall Survival  Rate@ | Overall Survival 95%CI | 91.35 | [75.7;98.4] | 57.60 | [46.0;69.7] | 40.07 | [10.1;91.47] | 91.42 | [79.0;97.9] | 71.75 | [48.5;91.0] |
|  |  |  |  |  |  |  |  |  |  |  |  |  |
| 60 mg/kg | Median^#^ | 95%CI | 348.00 | [343;348] | 366.00 | [345; -] | >377 | - | 342.00 | [341;343] | 345.00 | [343;348] |
|  | Day 360  Rate@ | Day 360 95%CI | 34.10 | [25.0;45.2] | 18.00 | [11.6;27.5] | 3.20 | [1.1;9.7] | 36.80 | [29.0;46.0] | 23.90 | [20.0;28.4] |
|  | Overall Survival  Day |  | 369 |  | 387 |  | 398 |  | 367 |  | 398 |  |
|  | Overall Survival  Rate@ | Overall Survival 95%CI | 69.74 | [51.9;85.8] | 80.34 | [45.0;98.8] | 49.36 | [18.3;45.3] | 90.33 | [77.7;97.4] | 75.52 | [52.4;74.2] |
|  |  |  |  |  |  |  |  |  |  |  |  |  |
|  | HR | 95%CI | 0.70 | [0.46;1.06] | 0.43 | [0.26;0.70] | 1.25 | [0.53;2.99] | 0.89 | [0.66;1.20] | 0.78* | [0.63;0.96] |
| * the country was accounted in the Cox Proportionnal Hazard Model | | | | | |  | | | | | | |
| Heterogeneity: Chi² = 3.72, df = 3 (P = 0.29); I² = 19% | | | | | | Test for overall effect: Z = 7.16 (P < 0.00001) | | | | |  |  |

Table S2. Post-day 21 reinfections.

Median infection-free survival (#), estimated rate (@), and hazard ratio (HR) between 40 and 60mg/kg
