# Supplementary material for: A Multicentre Randomized Controlled Trial of the Efficacy and Safety of Single-Dose Praziquantel at 40 mg/kg vs. 60 mg/kg for Treating Intestinal Schistosomiasis in the Philippines, Mauritania, Tanzania and Brazil
Source: PLoS Negl Trop Dis. 2011 Jun 14;5(6):e1165. doi: 10.1371/journal.pntd.0001165 (PMC3114749; doi:10.1371/journal.pntd.0001165)
Supplement: Table S3 — Intensity of infection at Day 180 and Day 360. (DOC) [file pntd.0001165.s009.doc]

Table S3. Intensity of infection at Day 180 and Day 360.

| Day 180 |  |  | 40 mg/kg | |  | 60mg/kg | |  |  |  |  |  |  | Day 360 | |  |  | 40 mg/kg | |  | 60mg/kg | |  |  |  |  |  |  |
| --- | --- | --- | --- | --- | --- | --- | --- | --- | --- | --- | --- | --- | --- | --- | --- | --- | --- | --- | --- | --- | --- | --- | --- | --- | --- | --- | --- | --- |
|  |  |  | N | % |  | N | % |  | OR | 95%CI | | p-value |  |  | |  |  | N | % |  | N | % |  | OR | 95%CI | | p-value |  |
| Philippines | 0 |  | 73 | (85.9%) |  | 75 | (86.2%) |  | 1.622 | (0.37; | 7.04) | 0.5181 |  | Philippines | | 0 |  | 47 | (55.3%) |  | 61 | (70.1%) |  | 1.059 | (0.40; | 2.84) | 0.9087 |  |
|  | **light** |  | 17 | (20.0%) |  | 12 | (13.8%) |  | 2.361 | (0.47; | 11.82) | 0.2959 |  |  | | **light** |  | 22 | (25.9%) |  | 13 | (14.9%) |  | 2.327 | (0.74; | 7.28) | 0.1465 |  |
|  | **moderate** |  | 3 | (3.5%) |  | 5 | (5.7%) |  | 1 |  |  |  |  |  | | **moderate** |  | 8 | (9.4%) |  | 11 | (12.6%) |  | 1 |  |  |  |  |
|  | **heavy** |  | 2 | (2.4%) |  | 2 | (2.3%) |  | 1.667 | (0.15; | 18.87) | 0.68 |  |  | | **heavy** |  | 8 | (9.4%) |  | 2 | (2.3%) |  | 5.496 | (0.91; | 33.14) | 0.0631 |  |
|  |  |  |  |  |  |  |  |  |  |  |  |  |  |  | |  |  |  |  |  |  |  |  |  |  |  |  |  |
| Brazil | 0 |  | 71 | (78.9%) |  | 83 | (92.2%) |  | 0.311 | (0.13; | 0.74) | 0.0084 |  | Brazil | | 0 |  | 54 | (60.0%) |  | 72 | (80.0%) |  | 1.25 | (0.29; | 5.46) | 0.7667 |  |
|  | **light** |  | 22 | (24.4%) |  | 8 | (8.9%) |  | 1 |  |  |  |  |  | | **light** |  | 32 | (35.6%) |  | 12 | (13.3%) |  | 4.444 | (0.92; | 21.53) | 0.0639 |  |
|  | **moderate** |  | 0 | (0.0%) |  | 0 | (0.0%) |  | ** |  |  |  |  |  | | **moderate** |  | 3 | (3.3%) |  | 5 | (5.6%) |  | 1 |  |  |  |  |
|  | **heavy** |  | 0 | (0.0%) |  | 0 | (0.0%) |  | ** |  |  |  |  |  | | **heavy** |  | 1 | (1.1%) |  | 1 | (1.1%) |  | 1.667 | (0.07; | 37.73) | 0.7483 |  |
|  |  |  |  |  |  |  |  |  |  |  |  |  |  |  | |  |  |  |  |  |  |  |  |  |  |  |  |  |
| Mauritania | 0 |  | 85 | (96.6%) |  | 89 | (98.9%) |  | ** |  |  |  |  | Mauritania | | 0 |  | 81 | (92.0%) |  | 79 | (87.8%) |  | 1.538 | (0.25; | 9.45) | 0.6422 |  |
|  | **light** |  | 0 | (0.0%) |  | 0 | (0.0%) |  | ** |  |  |  |  |  | | **light** |  | 4 | (4.5%) |  | 5 | (5.6%) |  | 1.2 | (0.13; | 11.05) | 0.8721 |  |
|  | **moderate** |  | 2 | (2.3%) |  | 0 | (0.0%) |  |  |  |  |  |  |  | | **moderate** |  | 2 | (2.3%) |  | 3 | (3.3%) |  | 1 |  |  |  |  |
|  | **heavy** |  | 1 | (1.1%) |  | 3 | (3.3%) |  | ** |  |  |  |  |  | | **heavy** |  | 1 | (1.1%) |  | 3 | (3.3%) |  | 0.5 | (0.03; | 8.95) | 0.6378 |  |
|  |  |  |  |  |  |  |  |  |  |  |  |  |  |  | |  |  |  |  |  |  |  |  |  |  |  |  |  |
| Tanzania | 0 |  | 86 | (76.1%) |  | 68 | (57.1%) |  | 0.692 | (0.24; | 2.00) | 0.497 |  | Tanzania | | 0 |  | 42 | (37.2%) |  | 43 | (36.1%) |  | 0.542 | (0.22; | 1.35) | 0.1886 |  |
|  | **light** |  | 28 | (24.8%) |  | 48 | (40.3%) |  | 1.5 | (0.49; | 4.58) | 0.4765 |  |  | | **light** |  | 57 | (50.4%) |  | 56 | (47.1%) |  | 0.52 | (0.21; | 1.26) | 0.1491 |  |
|  | **moderate** |  | 7 | (6.2%) |  | 8 | (6.7%) |  | 1 |  |  |  |  |  | | **moderate** |  | 9 | (8.0%) |  | 17 | (14.3%) |  | 1 |  |  |  |  |
|  | **heavy** |  | 1 | (0.9%) |  | 2 | (1.7%) |  | 1.75 | 0.129 | 23.703 | 0.6738 |  |  | | **heavy** |  | 5 | (4.4%) |  | 3 | (2.5%) |  | 0.318 | 0.061 | 1.644 | 0.1715 |  |
|  |  |  |  |  |  |  |  |  |  |  |  |  |  |  | |  |  |  |  |  |  |  |  |  |  |  |  |  |
| ALL | 0 |  | 297 | (77.7%) |  | 333 | (87.6%) |  | 0.823 | (0.37; | 1.83) | 0.6338 |  | ALL | | 0 |  | 225 | (58.9%) |  | 254 | (66.8%) |  | 0.781 | (0.34; | 1.82) | 0.5675 |  |
|  | **light** |  | 87 | (22.8%) |  | 48 | (12.6%) |  | 1.673 | (0.71; | 3.95) | 0.2409 |  |  | | **light** |  | 114 | (29.8%) |  | 87 | (22.9%) |  | 0.495 | (0.18; | 1.36) | 0.1717 |  |
|  | **moderate** |  | 13 | (3.4%) |  | 12 | (3.2%) |  | 1 |  |  |  |  |  | | **moderate** |  | 30 | (7.9%) |  | 28 | (7.4%) |  | 1 |  |  |  |  |
|  | **heavy** |  | 5 | (1.3%) |  | 6 | (1.6%) |  | 0.769 | (0.19; | 3.19) | 0.7178 |  |  | | **heavy** |  | 13 | (3.4%) |  | 11 | (2.9%) |  | 0.397 | (0.10; | 1.55) | 0.1833 |  |
| * General linear mixed models were used to account for the country  ** Not estimable* | | | | | | | | | | | | | | | * General linear lixed models were used to account for the country | | | | | | | | | | | | | |
