# Supplementary material for: A Multicentre Randomized Controlled Trial of the Efficacy and Safety of Single-Dose Praziquantel at 40 mg/kg vs. 60 mg/kg for Treating Intestinal Schistosomiasis in the Philippines, Mauritania, Tanzania and Brazil
Source: PLoS Negl Trop Dis. 2011 Jun 14;5(6):e1165. doi: 10.1371/journal.pntd.0001165 (PMC3114749; doi:10.1371/journal.pntd.0001165)
Supplement: Table S4 — Summary of safety findings at Day 0, 4 hours post-dosing. (DOC) [file pntd.0001165.s010.doc]

Table S4. Summary of safety findings at Day 0, 4 hours post-dosing

|  |  |  | **40 mg/kg** |  | **60 mg/kg** |  | **All** |  |  |  |  |
| --- | --- | --- | --- | --- | --- | --- | --- | --- | --- | --- | --- |
|  |  | **N** | **%** | **N** | **%** | **N** | **%** | **OR** | **95%CI** | | **p** |
| Philippines | **No AE** | 46 | (45.10%) | 14 | (13.86%) | 60 | (29.56%) | 1.00 |  |  |  |
|  | **>1 AE** | 56 | (54.90%) | 77 | (76.24%) | 133 | (65.52%) | 0.38 | (0.21; | 0.69) | 0.002 |
|  |  | 102 |  | 91 |  | 193 |  |  |  |  |  |
| Brazil | **No AE** | 34 | (35.05%) | 24 | (24.24%) | 58 | (29.59%) | 1.00 |  |  |  |
|  | **>1 AE** | 63 | (64.95%) | 75 | (75.76%) | 138 | (70.41%) | 0.59 | (0.00; | 1.10) | 0.097 |
|  |  | 97 |  | 99 |  | 196 |  |  |  |  |  |
| Mauritania | **No AE** | 25 | (26.88%) | 14 | (15.05%) | 39 | (20.97%) | 1.00 |  |  |  |
|  | **>1 AE** | 68 | (73.12%) | 79 | (84.95%) | 147 | (79.03%) | 0.48 | (0.00; | 1.00) | 0.048 |
|  |  | 93 |  | 93 |  | 186 |  |  |  |  |  |
| Tanzania | **No AE** | 12 | (8.89%) | 11 | (8.09%) | 23 | (8.49%) | 1.00 |  |  |  |
|  | **>1 AE** | 123 | (91.11%) | 125 | (91.91%) | 248 | (91.51%) | 1.11 | (0.00; | 2.61) | 0.813 |
|  |  | 135 |  | 136 |  | 271 |  |  |  |  |  |
| ALL | **No AE** | 117 | (27.40%) | 73 | (17.02%) | 190 | (22.20%) | 1.00 |  |  |  |
|  | **>1 AE** | 310 | (72.60%) | 356 | (82.98%) | 666 | (77.80%) | 0.54 | (0.39; | 0.76) | 0.000 |
|  |  | 427 |  | 429 |  | 856 |  |  |  |  |  |
